# Altered Monocyte Phenotype in HIV-1 Infection Tends to Normalize with Integrase-Inhibitor-Based Antiretroviral Therapy

**DOI:** 10.1371/journal.pone.0139474

**Published:** 2015-10-02

**Authors:** Marie R. McCausland, Steven M. Juchnowski, David A. Zidar, Daniel R. Kuritzkes, Adriana Andrade, Scott F. Sieg, Michael M. Lederman, Nicholas T. Funderburg

**Affiliations:** 1 Division of Infectious Disease, Case Western Reserve University School of Medicine, Cleveland, Ohio, United States of America; 2 Harrington Heart & Vascular Institute, University Hospitals Case Medical Center, Cleveland, Ohio, United States of America; 3 Division of Infectious Diseases, Brigham and Women’s Hospital, and Harvard Medical School, Boston, Massachusetts, United States of America; 4 Department of Medicine, Division of Infectious Diseases, John Hopkins University, Baltimore, Maryland, United States of America; 5 School of Health and Rehabilitation Sciences, Division of Medical Laboratory Science, Ohio State University, Columbus, Ohio, United States of America; Centre d'Immunologie et des Maladies Infectieuses, INSERM, FRANCE

## Abstract

**Background:**

Monocytes are increasingly implicated in the inflammatory consequences of HIV-1 disease, yet their phenotype following antiretroviral therapy (ART) initiation is incompletely defined. Here, we define more completely monocyte phenotype both prior to ART initiation and during 48 weeks of ART.

**Methods:**

Cryopreserved peripheral blood mononuclear cells (PBMCs) were obtained at baseline (prior to ART initiation) and at weeks 12, 24, and 48 of treatment from 29 patients participating in ACTG clinical trial A5248, an open label study of raltegravir/emtricitibine/tenofovir administration. For comparison, cryopreserved PBMCs were obtained from 15 HIV-1 uninfected donors, each of whom had at least two cardiovascular risk factors. Thawed samples were stained for monocyte subset markers (CD14 and CD16), HLA-DR, CCR2, CX3CR1, CD86, CD83, CD40, CD38, CD36, CD13, and CD163 and examined using flow cytometry.

**Results:**

In untreated HIV-1 infection there were perturbations in monocyte subset phenotypes, chiefly a higher frequency and density (mean fluorescence intensity–MFI) of HLA-DR (%-p = 0.004, MFI-p = .0005) and CD86 (%-p = 0.012, MFI-p = 0.005) expression and lower frequency of CCR2 (p = 0.0002) expression on all monocytes, lower CCR2 density on inflammatory monocytes (p = 0.045) when compared to the expression and density of these markers in controls’ monocytes. We also report lower expression of CX3CR1 (p = 0.014) on patrolling monocytes at baseline, compared to levels seen in controls. After ART, these perturbations tended to improve, with decreasing expression and density of HLA-DR and CD86, increasing CCR2 density on inflammatory monocytes, and increasing expression and density of CX3CR1 on patrolling monocytes.

**Conclusions:**

In HIV-1 infected patients, ART appears to attenuate the high levels of activation (HLA-DR, CD86) and to increase expression of the chemokine receptors CCR2 and CX3CR1 on monocyte populations. Circulating monocyte phenotypes are altered in untreated infection and tend to normalize with ART; the role of these cells in the inflammatory environment of HIV-1 infection warrants further study.

## Introduction

Monocytes are increasingly recognized as contributors to inflammation and coagulation in HIV-1 infection [1–3]. These antigen-presenting cells can be segregated into three functionally distinct populations based on CD14 and CD16 expression [4, 5]. “Traditional” monocytes express high levels of CD14, are lacking CD16 (CD14+CD16-), and produce pro-inflammatory cytokines in response to microbial elements, though to a lesser degree than do “inflammatory” monocytes (CD14+CD16+) [6]. “Patrolling” monocytes (CD14dimCD16+) produce IL6 and IL8 in response to viral elements, and patrol the vascular endothelium [6]. Increased proportions of both the inflammatory and patrolling monocytes have been reported previously in untreated HIV-1 infected patients when compared to the proportions in a healthy control population[1]. This nomenclature describes the function of these monocytes; others have characterized these cells as classical, intermediate and non-classical monocyte subsets respectively[5].

Since monocyte phenotype perturbations in HIV-1 infection and changes in monocyte phenotype with antiretroviral therapy (ART) are incompletely defined, we implemented a flow cytometry panel for cryopreserved cells that explored the expression and density of: activation and maturation markers, HLA-DR, CD38, CD13, and CD83; the co-stimulatory molecules CD40 and CD86; chemokine receptors CCR2 and CX3CR1; and the scavenger receptors CD36 and CD163. Using this monocyte phenotyping panel we found that in untreated HIV-1 infection there is lower density of CCR2 on inflammatory monocytes and lower expression of CX3CR1 on patrolling monocytes. We also found that untreated HIV-1-infected individuals had higher expression of HLA-DR and CD86 on total blood monocytes, and on most subsets, reflective of increased activation. Finally, we reported that many, but not all indices, normalized after ART.

## Methods

### Ethics Statement

This study was approved by institutional review boards at all participating sites: Brigham and Women's Hospital Clinical Research Site (CRS), Johns Hopkins Adult AIDS CRS, UCSD, AVRC CRS, University of Rochester ACTG CRS, AIDS Care CRS, Washington University CRS, The Ohio State University AIDS CRS, MetroHealth CRS, Northwestern University CRS, The Miriam Hospital ACTG CRS, Vanderbilt Therapeutics CRS, IHV Baltimore Treatment CRS, University of Colorado Hospital CRS, Houston AIDS Research Team CRS, and the Harlem ACTG CRS. Participants provided their written consent to participate in this study. This trial is registered with Clinicaltrials.gov # NCT00660972.

### Study Design

The study design has been more completely described in [7, 8], but briefly, A5248 was a prospective, open-label, multicenter, pilot study performed in the United States of America. Recruitment began June 2008 and ended January 2009; follow-up ended April 2010. A5248 was a single arm study of raltegravir (RAL, 400 mg twice daily) and emtricitabine/tenofovir disproxil fumarate (FTC/TDF, 200mg/300mg once daily) in HIV-1 infected ART-naïve patients who were enrolled if plasma HIV-1 RNA levels were >10,000 and <300,000 copies/mL. Patients were excluded from this analysis if they experienced virologic failure (plasma HIV-1 RNA level ≥ 1000 copies/mL between week 16 and week 24, or ≥ 200 copies/mL at or after 24 weeks) or clinical rebound (plasma HIV-1 RNA >0.3 log_10_ c/mL above the previous measurement).

### Sample Collection

Blood samples collected pre-entry and at study entry, week 12, week 24, and week 48 after ART initiation were utilized for analysis. Peripheral blood mononuclear cell (PBMC) samples were prepared by Ficoll density sedimentation and cryopreserved in 90% Fetal Bovine Serum (FBS) and 10% dimethyl sulfoxide (DMSO) until analyzed in batch. Cryopreserved samples available from patients who experienced virologic response to therapy, as described above, were used for these analyses. Baseline samples were pre-entry or entry samples according to availability. Patient characteristics are described in Table 1. Healthy control cryopreserved samples were obtained from a cohort of HIV uninfected persons without cardiovascular disease, but who had at least two cardiovascular risks including age > 50 years old, male gender, cigarette smoking, high blood pressure, high blood cholesterol, low HDL, Type II diabetes, or a history of cardiovascular disease in a first degree relative.

**Table 1:** Patient characteristics.

		HIV(+)	HIV(-)
		n = 29	n = 15
Gender	Female (%)	14	20
	Male (%)	86	80
Age (years)	Median	46	48
	Range	25–58	36–65
Demographics	White Non-Hispanic (%)	48	67
	Black Non-Hispanic (%)	24	16
	Hispanic (%)	24	0
	Asian or Pacific Islander (%)	0	7
	Other (%)	0	13
	Not Reported (%)	1	0
HIV Status	HIV-1 RNA	Median: 34469	——
	(copies/mL)	Range: 6644–264210	——
	CD4+ T cell Count	Median: 283	——
	(cells/uL)	Range: 10.5–547.5	——

HIV-1-infected patients are from the A5248 cohort and controls were taken from a cohort of uninfected persons with at least two cardiovascular disease risk factors.

### Flow Cytometry

Cryopreserved PBMCs were thawed and immediately stained for viability (LIVE/DEAD fixable Yellow Dead Cell Stain-Life Technologies, Grand Island, NY). All samples from the same patient were thawed and examined on the same day, and all patient and control samples were examined during a two month period. PBMCs were then washed twice with complete medium (RPMI- 10% FBS, 1% Pen/Strep, 1% Hepes, 1% L-glutamine) and stained for 30 minutes using a panel that excluded T cells, B cells, NK cells, and neutrophils (anti-CD3-Phycoerythrin-Cy7 (PE-Cy7) (557581-Becton Dickinson (BD), San Jose, CA), anti-CD15-PE-Cy7 (323030-Biolegend, San Diego, CA), anti-CD19-PE-Cy7 (302216-Biolegend), anti-CD56-PE-Cy7 (557747-BD)), and stained for monocyte markers using anti-CD14-Pacific Blue (558121-BD) and anti-CD16-Phycoerythrin (PE) (555407-BD), as well as for anti-HLA-DR-Peridinin chlorophyll protein (PerCP) (307628-Biolegend), anti-CD13- Allophycocyanin-Cy7 (APC-Cy7) (301710-Biolegend), anti-CD163-PE-CF594(562670-BD), anti-CD38-AF700 (560676-BD), anti-CCR2- PerCP-Cy5.5 (357203-Biolegend), anti-CX3CR1-APC (341610 Biolegend), anti-CD86-PerCP-Cy5.5 (305420-BD), anti-CD36-APC (550956-BD), CD40- AF700 (561208-BD). Cells were then fixed with 4% paraformaldehyde and examined using an LSRII flow cytometer (BD), which is calibrated daily by a dedicated technician using standard CS&T set up beads (BD Biosciences) to assure consistency of fluorescence detection.

Gates to identify positive expression of surface markers were determined using isotype control antibodies: MsIgG2a-Pacific Blue (558118-BD), MsIgG1- PE (555749-BD), MsIgG2a-PerCP (400250-Biolegend), MsIgG1-APC-Cy7 (400128-Biolegend), MsIgG1-PE-CF594 (562292-BD), MsIgG1-AF700 (557882-BD), MsIgG2a-PerCP-Cy5.5 (400252-Biolegend), Rat IgG2b-APC (400612-Biolegend), MsIgG2b-PerCP-Cy5.5 (400338-Biolegend), MsIgM-APC (555585-BD).

Data were analyzed using FACSDiva software (Version 6.2 BD Bioscience, San Diego CA). Monocytes were identified based on singlets, exclusion of viability dye, forward and side scatter characteristics, exclusion of cells with a high density of CD3, CD15, CD19, CD56, and were divided into the three subsets based on CD14 and CD16 expression. The CD14 and CD16 monocyte subset gates were based on the staining of their respective isotypes[1]. A “total monocyte” gate was also drawn around all three monocyte subsets. Total monocytes and monocyte subsets were characterized further by expression of HLA-DR, CD13, CD163, CD38, CCR2, CX3CR1, CD86, CD36, and CD40. Between 100,000 and 300,000 events were collected in the Forward/Side Scatter gate.

### Statistics

Comparisons between baseline findings in patients and among controls were performed using two-tailed Mann Whitney U tests. Comparisons among treatment time points were performed using both the generalized estimating equation (GEE) and two-tailed signed rank tests. Whereas the signed rank tests considered each pre- and post-ART comparison separately, the GEE allowed all the data to be considered together, and more fully considered repeated measures for each individual. Specifically, expression and density on monocytes prior to beginning ART was compared to the levels seen after ART initiation at week 12, week 24, and week 48 (Tables 2 and 3). GEE is best suited for studies with longitudinal data with correlated results [9]. Rather than using a repeated measures ANOVA, which requires a full dataset, we used GEE which does not require this approach. While our total HIV (+) patient population is 29, our final dataset includes 17 patients at baseline, 21 patients at week 12, 18 patients at week 24, and 23 patients at week 48, necessitating the GEE approach. Significance was defined as p ≤ 0.05. All graphs and analyses were performed using RStudio [10, 11] or Graphpad Prism Software (Version 5.04). Specifically, GEE was performed using the GEE function in RStudio[12].

**Table 2:** Alterations in frequencies of surface marker expression on total monocytes and monocyte subsets of HIV-1-infected patients before and after initiation of ART.

		GEE			GEE			Signed Rank test		
Proportion		Robust z			Robust P			p value		
		0–12	0–24	0–48	0–12	0–24	0–48	0–12	0–24	0–48
Total	*HLADR*	0.74	0.99	-0.92	0.459	0.322	0.358	0.328	0.839	0.255
	**CCR2**	0.48	0.48	1	0.631	0.631	0.317	*0*.*000*	*0*.*001*	*0*.*004*
	CX3CR1	1.8	-0.62	0.56	0.072	0.535	0.575	0.194	0.831	1.000
	CD38	-0.15	0.61	-0.04	0.881	0.542	0.968	0.572	0.831	**0.003**
	**CD13**	0.76	0.51	0.16	0.447	0.610	0.873	0.414	0.898	*0*.*021*
	CD40	-0.53	-1.95	-0.59	0.596	0.051	0.555	0.296	**0.014**	**0.040**
	*CD86*	-0.65	-1.93	-0.69	0.516	0.054	0.490	0.952	0.148	0.636
	CD36	-0.11	-0.57	-0.85	0.912	0.569	0.395	0.610	0.520	0.109
	CD163	0.18	0.48	0.53	0.857	0.631	0.596	0.903	0.898	0.701
	CD83	1.22	1.78	1.95	0.222	0.075	0.051	1.000	0.174	0.100
Traditional	*HLADR*	1.23	1.08	-1.26	0.219	0.280	0.208	0.117	0.824	0.272
	CCR2	*2*.*11*	1.45	*2*.*37*	*0*.*035*	0.147	*0*.*018*	*0*.*012*	0.102	*0*.*039*
	CX3CR1	*3*.*21*	0.46	0.92	*0*.*001*	0.646	0.358	*0*.*009*	0.123	0.784
	CD38	-0.27	0.05	-0.27	0.787	0.960	0.787	0.294	0.359	0.080
	**CD13**	1.06	1.01	0.99	0.289	0.312	0.322	0.217	0.898	0.305
	CD40	0.12	-0.98	0.25	0.904	0.327	0.803	0.730	0.148	*0*.*045*
	*CD86*	-0.31	**-2.06**	-0.8	0.757	**0.039**	0.424	0.761	0.123	0.636
	**CD36**	-0.19	-0.7	-0.75	0.849	0.484	0.453	0.754	0.765	0.327
	CD163	0.47	0.15	0.36	0.638	0.881	0.719	0.808	0.859	0.497
	CD83	1.44	1.73	1.85	0.150	0.084	0.064	0.423	1.000	0.181
Inflammatory	*HLADR*	-0.65	-1.82	**-2.7**	0.516	0.069	**0.007**	0.826	**0.042**	**0.036**
	CCR2	*2*.*38*	*2*.*59*	*2*.*14*	*0*.*017*	*0*.*010*	*0*.*032*	*0*.*049*	0.067	0.340
	CX3CR1	*3*.*21*	0.03	0.85	*0*.*001*	0.976	0.395	*0*.*013*	0.859	0.497
	CD38	-0.45	-0.28	-0.64	0.653	0.779	0.522	0.808	0.240	**0.004**
	CD13	0.31	0.48	-0.2	0.757	0.631	0.841	0.780	0.756	0.110
	CD40	-0.22	-1.62	-0.55	0.826	0.105	0.582	0.834	0.083	0.094
	CD86	-0.46	**-2.13**	-0.86	0.646	**0.033**	0.390	0.952	0.067	0.588
	**CD36**	0.4	-0.15	-0.61	0.689	0.881	0.542	0.706	0.520	0.224
	CD163	0.21	-0.03	0.15	0.834	0.976	0.881	0.583	0.700	0.946
	CD83	1.38	*2*.*02*	*2*.*34*	0.168	*0*.*043*	*0*.*019*	0.162	0.363	0.154
Patrolling	*HLADR*	-0.18	-0.13	**-2.13**	0.857	0.897	**0.033**	0.761	0.610	0.127
	CCR2	0.64	0.6	1.64	0.522	0.549	0.101	0.855	0.520	0.839
	**CX3CR1**	*3*.*34*	1.7	*2*.*3*	0.001	0.089	*0*.*021*	*0*.*001*	0.067	0.152
	CD38	-1.87	**-2.43**	**-2.53**	0.061	**0.015**	**0.011**	0.191	**0.010**	**0.001**
	CD13	0.11	0.45	-0.41	0.912	0.653	0.682	0.414	0.831	0.255
	CD40	-1.5	**-3.18**	**-2.96**	0.134	**0.001**	**0.003**	0.153	**0.002**	**0.000**
	CD86	0.25	-1.77	-0.99	0.803	0.077	0.322	0.761	0.413	0.455
	CD36	-1.81	**-2.71**	**-2.71**	0.070	**0.007**	**0.007**	**0.025**	**0.024**	**0.008**
	CD163	-1.02	-0.21	-0.51	0.308	0.834	0.610	0.391	0.240	0.787
	CD83	1.31	*2*.*02*	1.89	0.190	*0*.*043*	0.059	0.208	0.083	0.168

Baseline proportions are italicized if significantly greater than among uninfected controls and bolded if significantly lower by Mann Whitney U tests. Comparisons were made between week 12 and baseline (0–12), week 24 and baseline (0–24), and week 48 and baseline (0–48) using both Wilcoxon signed rank test and the generalized estimating equation. Significant increases (p<0.05) are italicized and significant decreases are bolded. Robust Z score for the GEE plot are included to aid in the understanding of the direction of the change; negative Z score indicates decreased values after ART initiation and positive Z scores indicate increased values after ART initiation.

**Table 3:** Alterations in surface marker density on total monocytes and monocyte subsets of HIV-1-infected patients before and after initiation of ART.

		GEE			GEE			Signed Rank test		
MFI		Robust z			Robust P			p value		
		0–12	0–24	0–48	0–12	0–24	0–48	0–12	0–24	0–48
Total	*HLADR*	-1.710	**-2.740**	**-5.730**	0.087	**0.006**	**0.000**	0.194	0.175	**0.001**
	CCR2	*5*.*060*	*4*.*070*	*4*.*770*	*0*.*000*	*0*.*000*	*0*.*000*	*0*.*000*	*0*.*003*	*0*.*003*
	CX3CR1	0.950	-0.610	0.570	0.342	0.542	0.569	0.727	1.000	1.000
	CD38	-0.410	-1.350	**-2.450**	0.682	0.177	**0.014**	0.715	0.831	**0.011**
	CD13	-1.220	-1.610	**-3.610**	0.222	0.107	**0.000**	0.593	0.278	**0.000**
	CD40	**-2.540**	**-2.660**	-1.130	**0.011**	**0.008**	0.258	**0.035**	**0.024**	0.056
	*CD86*	-1.940	**-2.400**	-0.680	0.052	**0.016**	0.497	0.068	**0.024**	**0.040**
	CD36	0.680	-0.190	**-2.580**	0.497	0.849	**0.010**	0.675	1.000	0.080
Traditional	*HLADR*	-0.350	-1.490	**-3.470**	0.726	0.136	**0.001**	0.855	0.700	**0.013**
	CCR2	*3*.*290*	*1*.*970*	*2*.*940*	*0*.*001*	*0*.*049*	*0*.*003*	*0*.*001*	*0*.*032*	0.080
	CX3CR1	1.440	-0.010	0.880	0.150	0.992	0.379	0.224	0.154	1.000
	*CD38*	-1.150	**-2.440**	**-3.290**	0.250	**0.015**	**0.001**	1.000	0.278	**0.006**
	CD13	-0.460	-1.040	**-2.840**	0.646	0.298	**0.005**	0.952	0.638	**0.000**
	**CD40**	-0.590	-1.160	0.120	0.555	0.246	0.904	0.802	0.123	0.083
	*CD86*	-1.430	-1.840	-0.160	0.153	0.066	0.873	0.135	**0.042**	0.057
	CD36	0.240	-0.640	**-2.620**	0.810	0.522	**0.009**	0.442	0.839	0.069
Inflammatory	*HLADR*	-1.530	-1.880	**-4.050**	0.126	0.060	**0.000**	0.626	0.320	**0.002**
	**CCR2**	*3*.*360*	*3*.*310*	*3*.*500*	*0*.*001*	*0*.*001*	*0*.*000*	*0*.*033*	*0*.*005*	0.127
	CX3CR1	*3*.*230*	0.570	1.610	*0*.*001*	0.569	0.107	*0*.*012*	0.221	0.266
	CD38	0.170	-0.580	**-2.440**	0.865	0.562	**0.015**	0.808	0.966	**0.021**
	CD13	**-2.510**	**-2.690**	**-4.610**	**0.012**	**0.007**	**0.000**	0.119	**0.024**	**0.000**
	CD40	-0.720	-1.510	-1.340	0.472	0.131	0.180	0.808	0.230	0.055
	*CD86*	**-2.290**	**-2.980**	-1.390	**0.022**	**0.003**	0.165	**0.049**	**0.007**	0.110
	CD36	1.110	0.160	**-2.410**	0.267	0.873	**0.016**	1.000	0.959	0.110
Patrolling	HLADR	**-2.290**	-1.830	**-4.070**	0.022	0.067	**0.000**	**0.013**	0.123	**0.001**
	CCR2	-0.430	0.600	0.900	0.667	0.549	0.368	1.000	1.000	1.000
	CX3CR1	*5*.*160*	*2*.*590*	1.920	*0*.*000*	*0*.*010*	0.055	*0*.*001*	*0*.*042*	0.147
	CD38	**-3.750**	**-3.850**	**-2.680**	**0.000**	**0.000**	**0.007**	**0.001**	**0.004**	**0.021**
	CD13	**-3.380**	**-3.540**	**-4.900**	**0.001**	**0.000**	**0.000**	**0.013**	**0.032**	**0.001**
	CD40	**-2.360**	**-3.450**	**-4.600**	**0.018**	**0.001**	**0.000**	**0.049**	**0.014**	**0.003**
	CD86	-1.770	**-2.810**	**-3.560**	0.077	**0.005**	**0.000**	0.078	0.083	**0.021**
	CD36	-0.510	-1.570	**-3.370**	0.610	0.116	**0.001**	0.119	0.206	0.057

Baseline MFI values are italicized if significantly greater than among uninfected controls and boldedif significantly lower by Mann Whitney U tests. Comparisons were made between week 12 and baseline (0–12), week 24 and baseline (0–24), and week 48 and baseline (0–48) using both Wilcoxon signed rank test and the generalized estimating equation. Significant increases (p<0.05) are italicized and significant decreases are bolded. Robust Z score for the GEE plot are included to aid in the understanding of the direction of the change; negative Z score indicates decreased values after ART initiation and positive Z scores indicate increased values after ART initiation

## Results

### Patient and Control Samples

HIV-1 infected patients were 86% male, with a median age of 46, and fewer than half were White non-Hispanic (Table 1). Before ART initiation, the median plasma HIV-1 RNA and CD4+ T cell count were 34,469 copies/mL and 283 cells/uL respectively. The control population was 80% male, 67% White non-Hispanic, with a median age of 48, and one-third of the subjects were cigarette smokers. Smoking data were not available for the HIV-1 infected patients.

### Monocyte Phenotypes in Fresh and Cryopreserved PBMCS

Multicenter clinical trials designed to monitor immune cell subsets typically utilize cryopreserved samples since multi-parameter flow cytometry using fresh blood samples has not yet been standardized to support on-site performance at multiple centers. While most T cell phenotypes are stable with cryopreservation [13], the stability of monocyte phenotypes after cryopreservation is not adequately described, and certain monocyte functions have been found to be diminished after cryopreservation [14, 15]. We therefore needed to determine which monocyte surface markers were relatively unaltered with cryopreservation.

We obtained peripheral blood mononuclear cell (PBMC) samples prepared by Ficoll density sedimentation of EDTA-anti-coagulated whole blood from four healthy controls and four HIV-1-infected subjects and stained a portion of each sample with our monocyte phenotyping panel. The remaining cells were cryopreserved and later thawed and stained using the same phenotyping panel.

**Fig 1:**
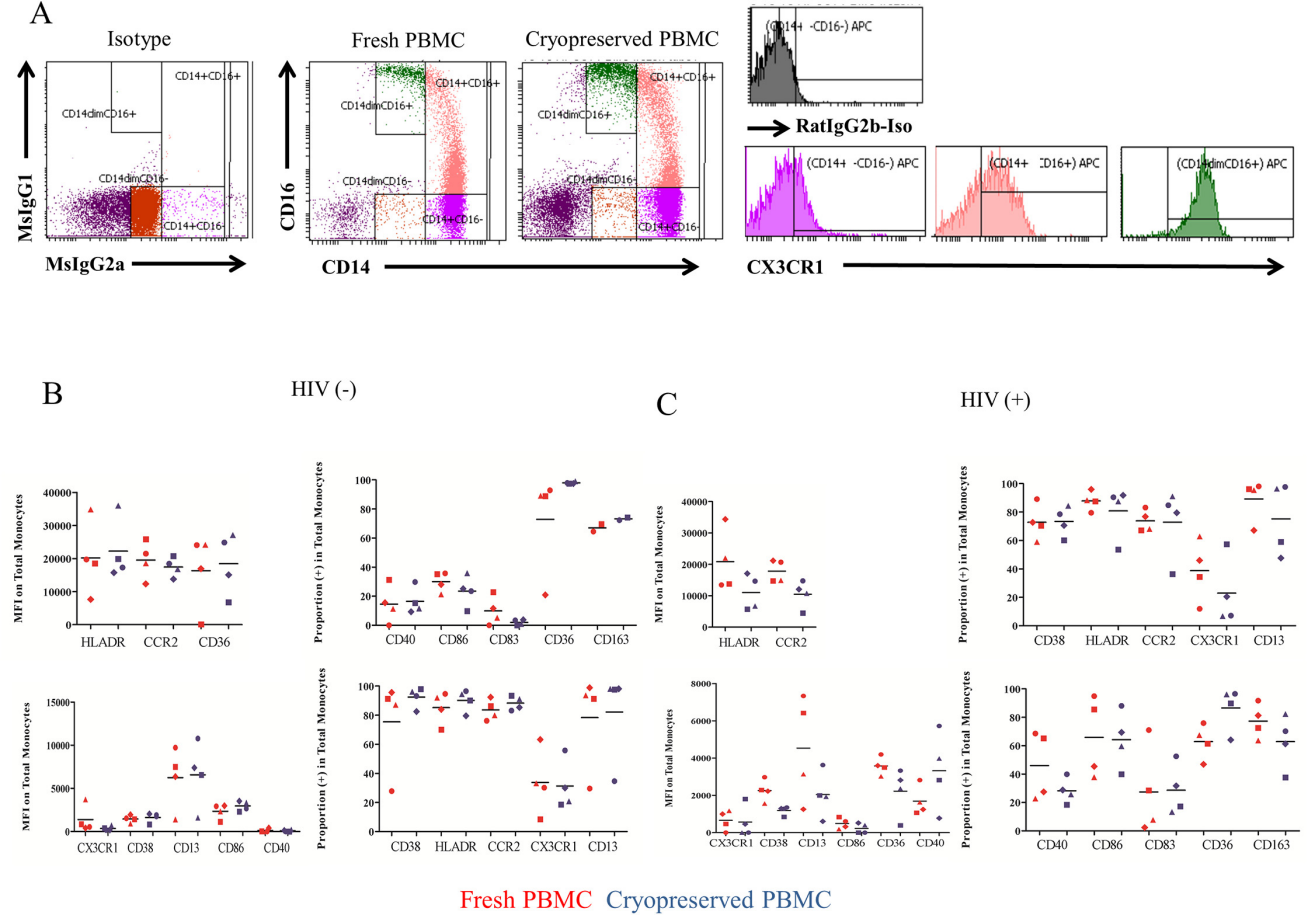
Gating strategy for flow cytometry and comparison between fresh and cryopreserved monocyte surface marker expression. (A) Shown are isotype control dot plots and CD14 and CD16 expression in freshly obtained and cryopreserved PBMC from the same healthy volunteer and the expression of CX3CR1 on each monocyte subset in the cryopreserved sample. Monocyte subsets were gated using the isotype staining as a guide, as seen in the farthest left panel. Traditional monocytes are in purple, inflammatory monocytes are in pink, patrolling monocytes are in green. Gates are drawn based on negative isotype staining using Rat IgG2b, in the case of CX3CR1. (B) Summary surface marker expression, both proportion and MFI, in fresh and cryopreserved PBMCs on total monocytes of healthy control subjects, with medians, are shown. Each shape (triangle, square, diamond, and circle) represents an individual healthy control subject. (C) Summary surface marker expression, both proportion and MFI, in fresh and cryopreserved PBMCs on total monocytes of virologically suppressed HIV-infected subjects, with medians, are shown. Each shape (triangle, square, diamond, and circle) represents an individual HIV-infected subject.

Monocyte subset distributions in fresh and cryopreserved monocytes are similar (Fig 1A). Expression of CD40, CD163, CD86, CD38, HLA-DR, CCR2, CX3CR1, and CD13 on monocytes was consistent in fresh and cryopreserved PBMCs of healthy (Fig 1B) and virologically suppressed HIV-1-infected subjects (Fig 1C), though the HIV-1-infected subjects appear to have higher variability in staining. The density (MFI) of CD36 appeared to be greater in the cryopreserved PBMCs of the healthy subjects, though this was driven by relatively low expression of CD36 in the fresh preparation from one healthy subject. Although the expression of CD83 was relatively diminished in cryopreserved PBMCs of the controls, we elected to retain this marker in the panel as CD83 expression is a marker of monocyte maturation that increases in response to viral products[16]. In contrast, the expression of LOX1, PDL1 and PDL2 was diminished so dramatically after cryopreservation that these markers were excluded from the panel (data not shown).

### Altered Proportions of Monocyte Subsets in Untreated HIV-1 Infection

**Fig 2:**
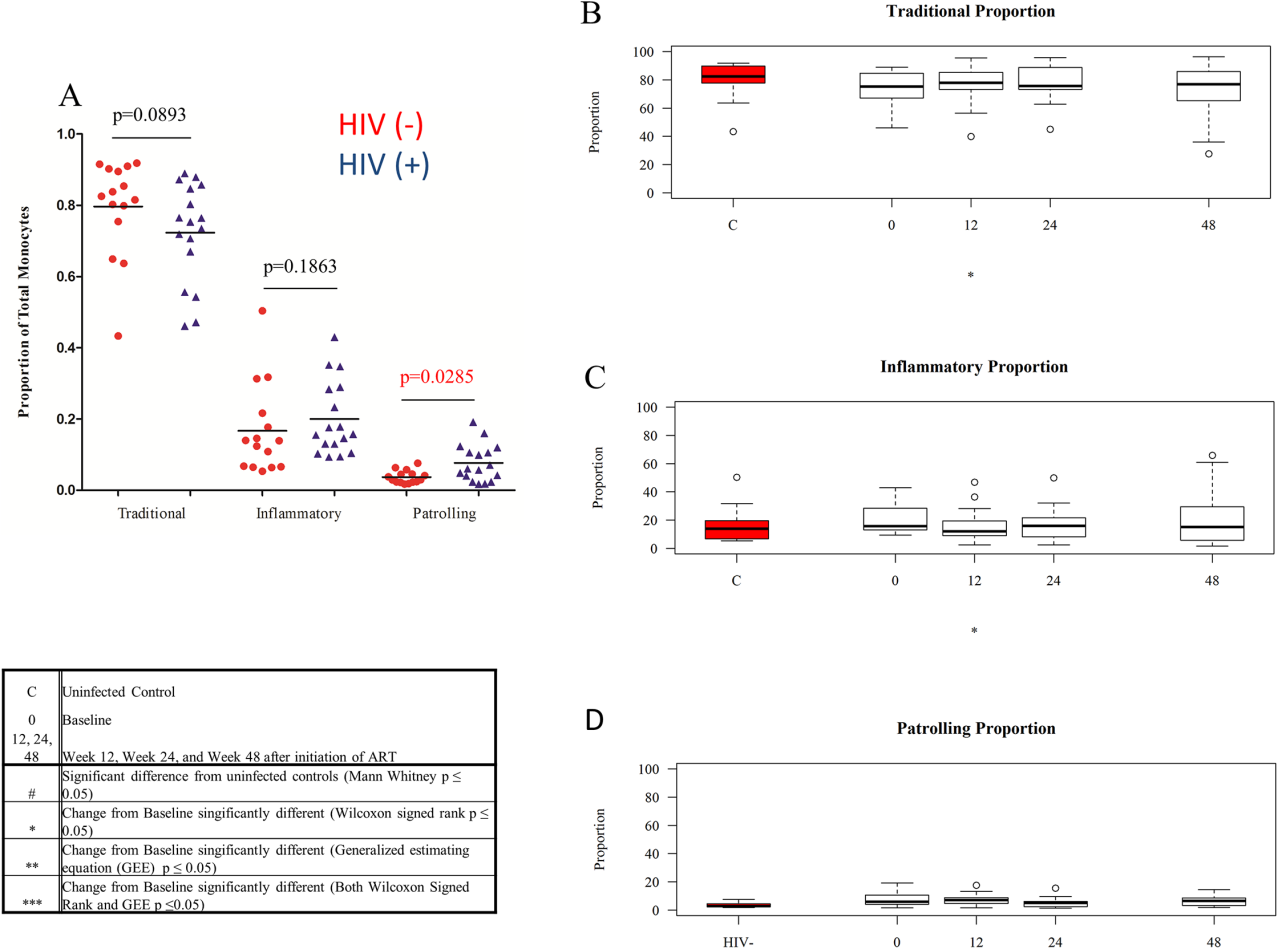
Monocyte subset proportions at baseline and after ART initiation compared to proportions among controls. (A) Jitterplot comparing the subset proportions in HIV-1-infected individuals prior to ART initiation and subset proportions in controls. Medians are shown, and p values were determined using Mann Whitney U tests. Figs B-D display Tukey boxplots of medians and interquartile ranges. Outliers are shown as open circles. Tukey boxplots show the proportions of traditional monocytes (B), inflammatory monocytes (C) and patrolling monocytes (D) in controls (red) and in HIV-1-infected subjects at baseline and over the course of 48 weeks of ART.

In earlier work using fresh whole blood samples, we found lower proportions of traditional monocytes, and increased proportions of inflammatory and patrolling monocytes in HIV-1 infected patients with uncontrolled viremia [1]. Our new data evaluating a smaller number of cryopreserved samples from different patient and control populations are similar. The proportion of traditional (CD14+CD16-) monocytes tended to be lower in HIV-1-infectedpatients (median-76.6%) compared to the proportion of traditional monocytes among control subjects (median-82.5%), though not significantly lower (p = .089). The proportion of inflammatory (CD14+CD16+) monocytes also tended to be higher in the setting of HIV-1 infection (median-14.8%) than among controls (median-13.9%), though not significantly (p = 0.19). The proportion of patrolling (CD14dimCD16+) monocytes was significantly higher in HIV-1 infection (median-6.0%) than among controls (median-2.9%, p = .029, Fig 2A). These subset proportions did not change with ART (Fig 2B–2D).

### Monocyte Phenotypes are Altered in Untreated HIV-1 Infection and Change with ART

**Fig 3:**
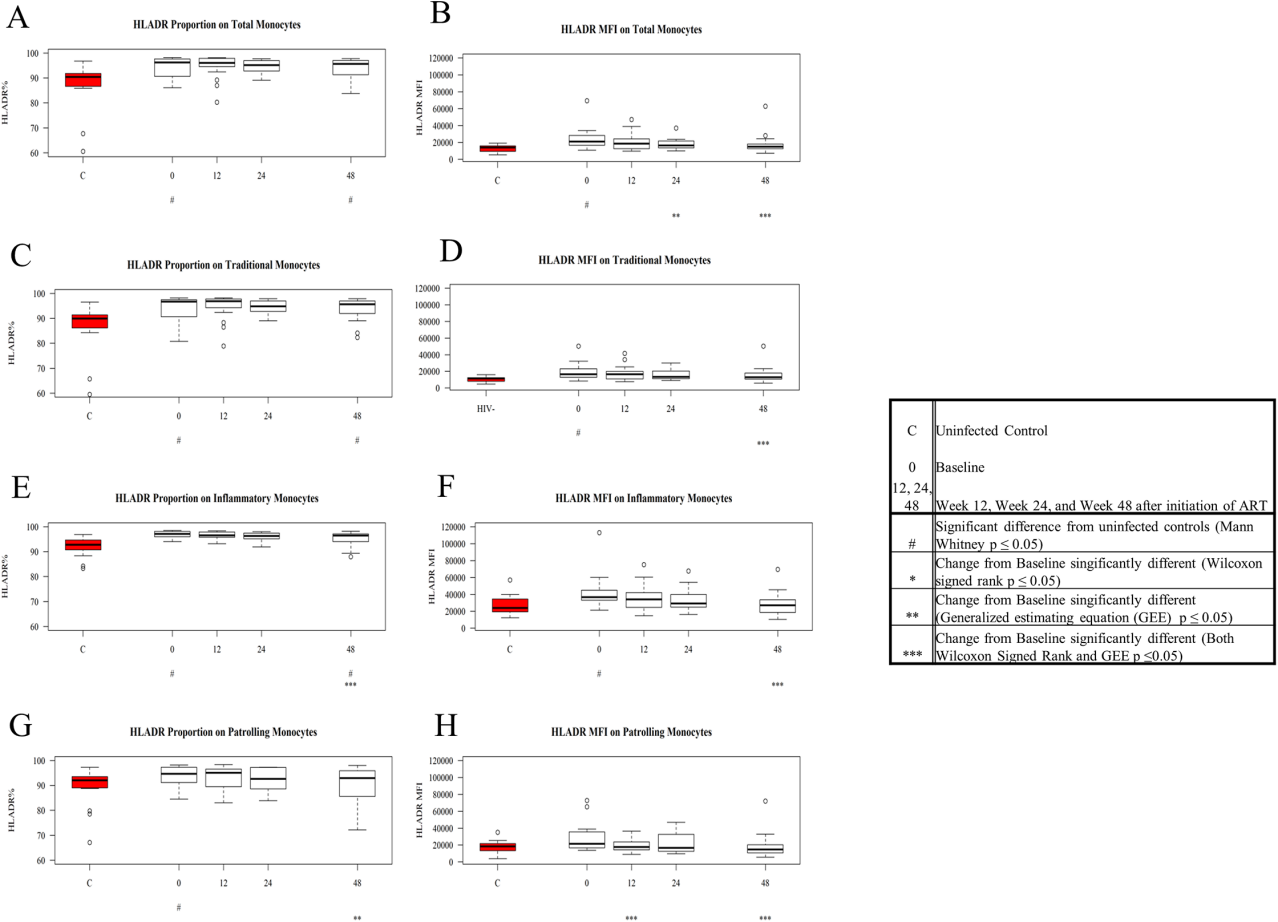
Expression and density (MFI) of HLA-DR on patient monocytes at baseline and after ART initiation compared to values among controls. Values for frequency and density of HLA-DR on monocyte subsets in untreated HIV-1 infection were compared to levels among controls (in red) using Mann Whitney U tests, and baseline values among patients were compared to values on ART using GEE and Signed Rank test (see boxed legend). Figures show Tukey boxplots of medians and interquartile ranges, outliers are shown as open circles. HLA-DR densities and proportions were increased at baseline when compared to the levels seen in the controls (A-G). Tukey boxplots show the proportions of HLA-DR+ total monocytes (A), traditional monocytes (C), inflammatory monocytes (E), and patrolling monocytes (G) in controls’ samples (red) and in patient samples at baseline and after ART initiation. Tukey boxplots show the density of HLA-DR on total monocytes (B), traditional monocytes (D), inflammatory monocytes (F), and patrolling monocytes (H) in controls’ samples (red) and in patient samples at baseline and after ART initiation.

**Fig 4:**
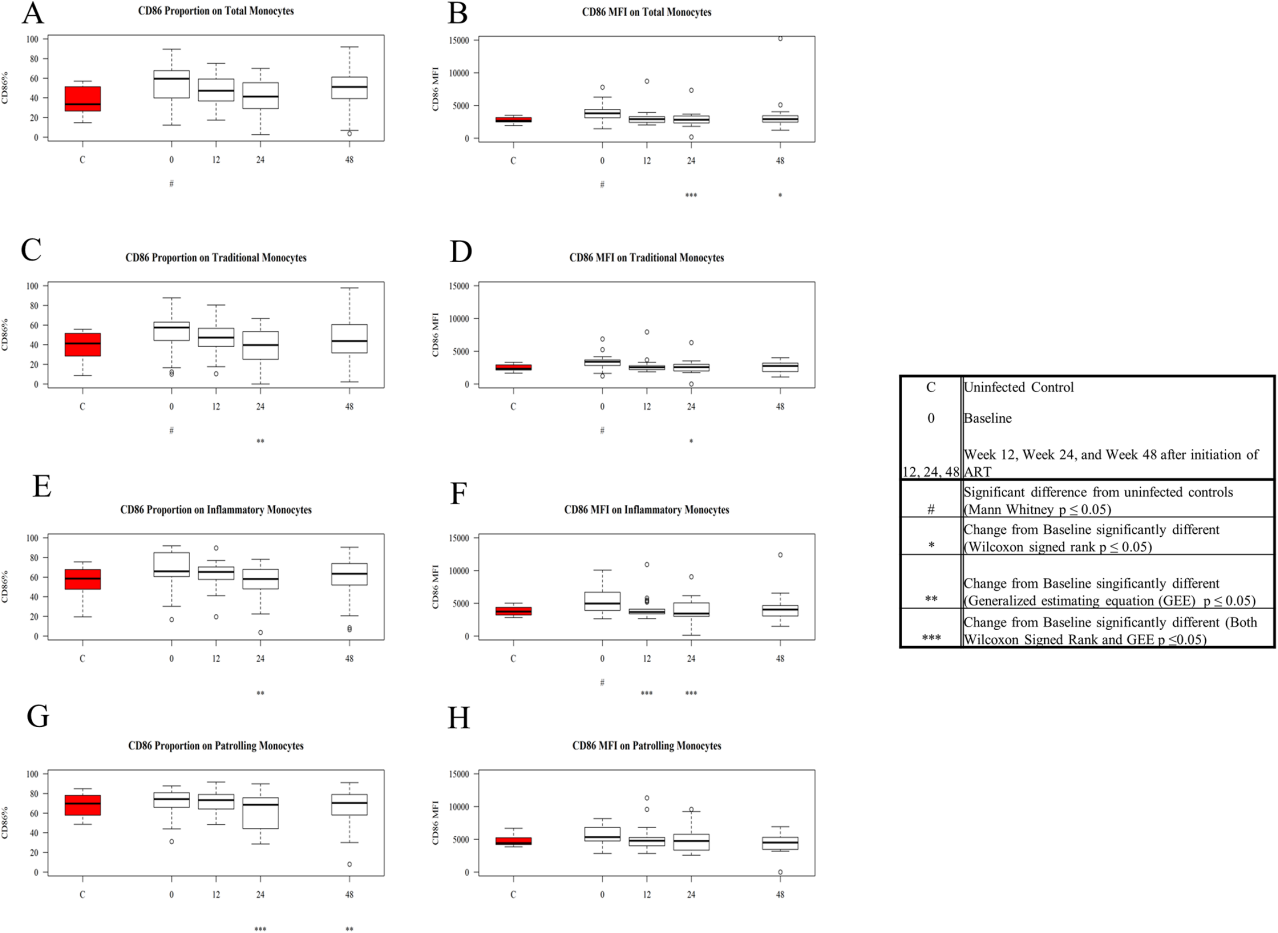
Expression and density (MFI) of CD86 on patient monocytes at baseline and after ART initiation compared to values among controls. Values for frequency and density of CD86 on monocyte subsets in untreated HIV-1 infection were compared to levels among controls (in red) using Mann Whitney U tests, and baseline values among patients were compared to values on ART using GEE and Signed Rank test (see boxed legend). Figures show Tukey boxplots of medians and interquartile ranges; outliers are shown as open circles. Tukey boxplots show the proportion of CD86+ total monocytes (A), traditional monocytes (C), inflammatory monocytes (E), and patrolling monocytes (G) in control samples (red) and in patient samples at baseline and after ART initiation. Tukey boxplots show the density of CD86 on total monocytes (B), traditional monocytes (D), inflammatory monocytes (F), and patrolling monocytes (H) in control samples (red) and in patient samples at baseline and after ART initiation.

**Fig 5:**
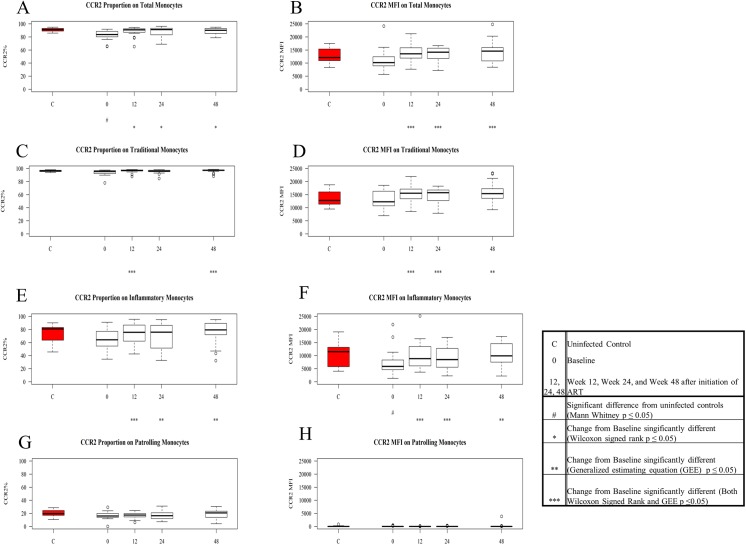
Expression and density (MFI) of CCR2 on patient monocytes at baseline and after ART initiation compared to values among controls. Values for frequency and density of CCR2 on monocyte subsets in untreated HIV-1 infection were compared to levels among controls using Mann Whitney U tests, and baseline values among patients were compared to values after ART initiation using GEE and Signed Rank test (see boxed legend). Figures show boxplots of medians and interquartile ranges; outliers are shown as open circles. Tukey boxplots show the proportion of CCR2+ total monocytes (A), traditional monocytes (C), inflammatory monocytes (E), and patrolling monocytes (G) in control samples (red) and in patient samples at baseline and after ART initiation. Tukey boxplots show the density of CCR2 on total monocytes (B), traditional monocytes (D), inflammatory monocytes (F), and patrolling monocytes (H) in control samples (red) and in patient samples at baseline and after ART initiation.

**Fig 6:**
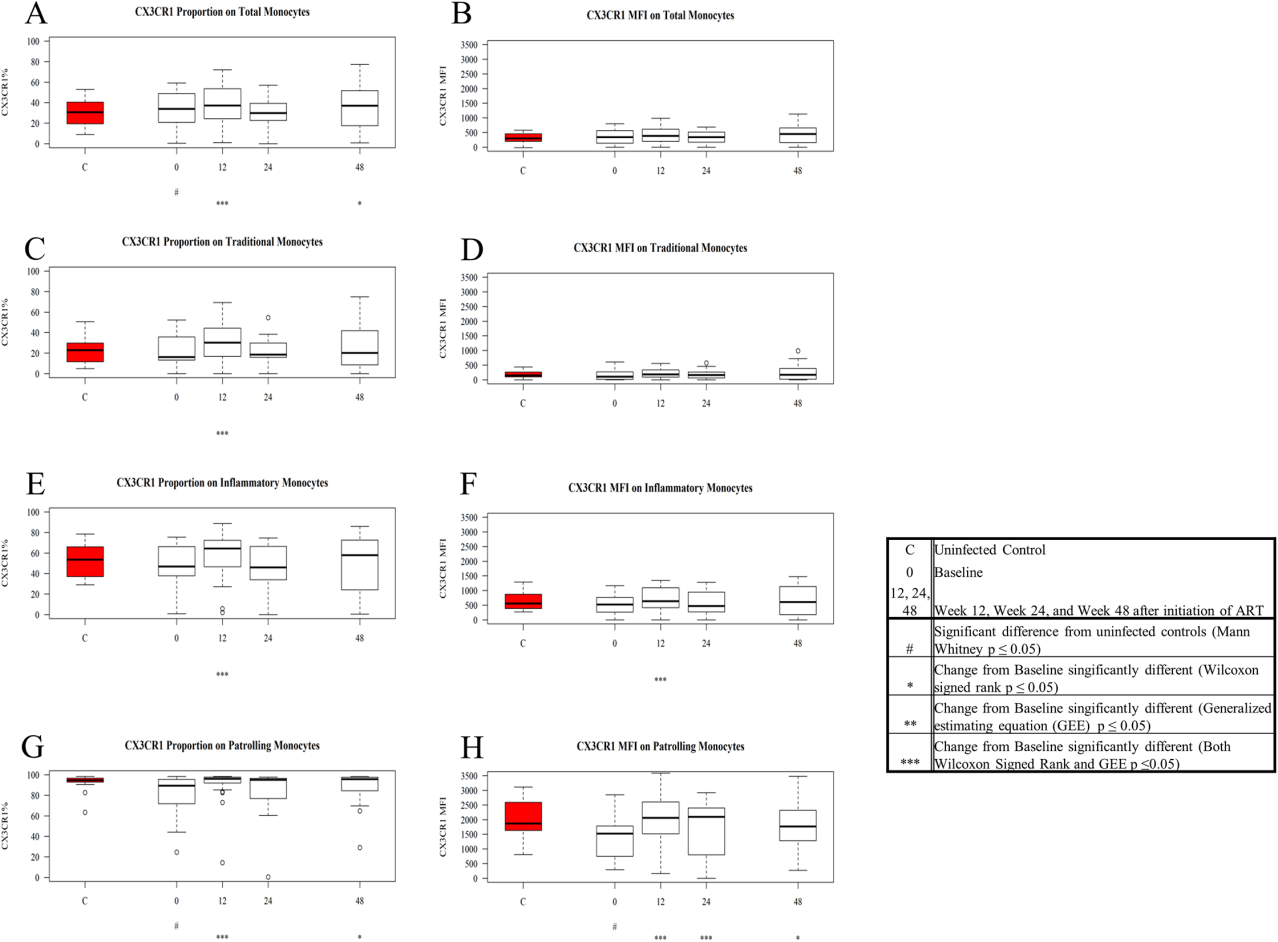
Expression and density (MFI) of CX3CR1 on patient monocytes at baseline and after ART initiation compared to values among controls. Values for frequency and density of CX3CR1 on monocytes in untreated HIV-1 infection were compared to levels among controls using the Mann Whitney U test, and baseline values were compared to values after ART initiation using GEE and Signed Rank test. Figures show boxplots of medians and interquartile ranges; outliers are shown as open circles. Tukey boxplots show the proportion of CX3CR1+ total monocytes (A), traditional monocytes (C), inflammatory monocytes (E), and patrolling monocytes (G) in control samples (red) and in patient samples at baseline and after ART initiation. Tukey boxplots show the density of CX3CR1 on total monocytes (B), traditional monocytes (D), inflammatory monocytes (F), and patrolling monocytes (H) in control samples (red) and in patient samples at baseline and after ART initiation.

Shown on Tables 2 and 3 are summaries of the baseline and on treatment data sets presented in this manuscript. Proportions and mean fluorescent intensities that differ significantly at baseline from controls’ values are shown in red (greater in patients) and green (lower in patients). Likewise, significant changes from baseline in these values during ART are shown in red if they rise and in green if they diminish. Selected graphic distributions of these phenotypes are also shown in Figs 3–6. The means and standard errors of the means are shown in S1 and S2 Tables.

### Greater HLA-DR Expression and Density in Untreated HIV-1 Infection, Tend to Decrease with ART

HLA-DR is utilized for presentation of peptide antigen to CD4+ T cells [17, 18] and HLA-DR expression on monocytes typically increases in the setting of activation [19]. We found that both frequency of HLA-DR expression and the HLA-DR mean fluorescence intensity (MFI) on total monocytes were significantly greater in patient samples (p = 0.004, 0.0005 respectively) than they were among controls’ samples (Fig 3A and 3B). With initiation of ART, the proportions of HLA-DR+ monocytes did not decrease significantly and remained elevated when compared to proportions among healthy controls. HLA-DR density (MFI) decreased significantly on total monocytes at weeks 24 and 48. At baseline, the frequency of HLA-DR expression was higher on each monocyte subset in the setting of HIV-1 infection (traditional-p = 0.002; inflammatory-p<0.0001; patrolling-p = 0.039) compared to the frequency of HLA-DR expression on monocyte subsets from controls. When these monocyte subpopulations were examined individually, both the proportions and density of HLA-DR decreased significantly on both inflammatory and patrolling monocytes at week 48; the frequencies of HLA-DR expression on these subsets was no longer different from these frequencies in subsets from controls’ samples (Fig 3E and 3G). At baseline, HLA-DR density on traditional (p = 0.003) and inflammatory (p = 0.003) monocytes was significantly higher on samples from HIV-1-infected subjects compared to HLA-DR density on samples from controls (Fig 3D and 3F). Density of HLA-DR tended to be higher on the patrolling monocytes of patients when compared to the density on controls’ samples, but these differences were not significant (p = 0.0893) (Fig 3H). Density of HLA-DR decreased on all monocyte subsets after 48 weeks of ART (Fig 3D and 3F).

### Greater CD86 Expression and Density on Monocytes in Untreated HIV-1 Infection decrease with ART

CD86 is a surface receptor that binds T cell CD28 and aids in co-stimulation [20, 21]. The proportion of total monocytes expressing CD86 was significantly greater in the untreated HIV-1-infected patients (p = 0. 012), and CD86 density was significantly higher (p = 0.005) among patients’ cells than among controls’ cells (Fig 4A and 4B). The proportion of CD86+ monocytes did not change with treatment while CD86 density decreased significantly (by GEE and signed rank test) at week 24 and by signed rank test at week 48, achieving levels not different from levels seen in controls.

Both the proportion of CD86 expressing traditional monocytes and the density of CD86 on traditional monocytes were significantly higher in untreated HIV-1 infection compared to the levels seen in the healthy control population (p = 0.008, p = 0.009 respectively) (Fig 4C and 4D). After 48 weeks of ART, both the density and expression of CD86 on traditional monocytes fell to levels no longer different from those seen among controls and the changes from baseline were significant at week 24.

The proportion of inflammatory monocytes expressing CD86 tended to be greater in untreated HIV-1 infection (p = 0.050) and the MFI of CD86 on these cells was greater in patients than among controls (p = 0.004) (Fig 4E and 4F). The proportions of CD86+ patrolling monocytes decreased from baseline at weeks 24 and 48, and at these times neither their frequencies nor MFIs were different from those seen among controls’ cells (Fig 4G and 4H).

### Lower CD40 Density on Traditional Monocytes in Untreated HIV-1 Infection

In monocytes, the ligation of CD40 can result in the production of several pro-inflammatory cytokines, such as IL-6 and IL-1β, and also can result in greater co-stimulatory molecule expression through interaction with CD40 ligand on CD4 T cells [22]. The proportions of monocytes expressing CD40 were not significantly different from the proportions in controls (total-p = 0.75, traditional-p = 0.33; inflammatory-p = 0.97; patrolling-p = 0.92), however, the density of CD40 was marginally lower on traditional monocytes in HIV-1 infection (total-p = 0.71; traditional-p = 0.048; inflammatory-p = 0.38; patrolling-p = 0.89) and the density of CD40 on total monocytes and on patrolling monocytes decreased significantly from baseline after 48 weeks of ART (Table 3).

### Chemokine Receptor (CCR2 and CX3CR1) Expression on Inflammatory and Patrolling Monocyte Subsets is Lower in Untreated HIV-1 Infection and Normalizes with ART

We next examined the distribution of the chemokine receptors CCR2 and CX3CR1 on monocyte subsets (Figs 5 and 6). As expected, traditional monocytes had the highest expression of CCR2 and the lowest expression of CX3CR1, and, conversely, patrolling monocytes had the highest expression of CX3CR1 and the lowest expression of CCR2, with expression of these molecules on inflammatory monocytes falling in between[23].

At baseline, the proportion of total monocytes expressing CCR2 was lower in patients (p = 0.0002) when compared to the proportion of CCR2+ monocytes in controls (Fig 5A) and there was a trend towards lower CCR2 density (MFI, p = 0.053) on patient monocytes (Fig 5B). At baseline, inflammatory monocytes from patients tended to less frequently express CCR2 (p = 0.057) and to have lower CCR2 MFIs (p = 0.045) compared to inflammatory monocytes from controls (Fig 5E and 5F). There were no significant differences in CCR2 expression at baseline among traditional (MFI-p = 0.55; %- p = 0.14) (Fig 5C and 5D) or patrolling subsets (MFI-p = 0.075; %- p = 0.097) (Fig 5G and 5H) compared to expression levels and frequencies on controls’ monocytes. Both the proportion of CCR2+ monocytes and the CCR2 densities increased from baseline by week 12 of ART on total monocytes and on both traditional and inflammatory populations, and became comparable to levels seen among controls.

The frequencies and densities of the fractalkine receptor (CX3CR1) on total monocytes, traditional monocytes, and inflammatory monocytes, were comparable in samples from controls and patients at baseline (Fig 6A–6F). Though they were unaltered at baseline, the proportions of CX3CR1+ traditional and inflammatory monocytes, and the density of this receptor, rose from baseline at 12 weeks (Fig 6C and 6E). Among patrolling monocytes from patient samples, both the proportion of CX3CR1+ cells and CX3CR1 density were lower than among controls at baseline (p = 0.0453, p = 0.0235 respectively) and rose by week 12 of ART to be no longer different from these values among controls’ cells (Fig 6G and 6H).

CD13 is a homotypic cell adhesion molecule which is expressed on both monocytes and endothelial cells, and is thought to play a role in monocyte adhesion and migration, and is used as a marker of monocyte maturation [24]. The proportion of CD13+ monocytes was lower (p = 0.013) in untreated HIV-1 infection and this was related to low frequencies of CD13 expression in a subset of patients. The proportion of total monocytes expressing CD13 was decreased further after 48 weeks on treatment (Table 2). At baseline, the proportion of CD13+ traditional monocytes was significantly lower in samples from patients than among samples from controls (p = 0.019), but this was not seen among inflammatory (p = 0.37) or patrolling subsets (p = 0.48).

## Discussion

Our study examined the phenotype of monocytes and their subsets in untreated HIV-1 infection, after ART initiation, and in a healthy control population also at risk for coronary artery disease. We were able to confirm previous reports demonstrating an increased proportion of patrolling monocytes in HIV-1 infection [8] and we also provide new insights into the kinetics of ART effects on monocyte phenotype in HIV-1 disease.

We report here the phenotypes of total circulating monocytes, as well as the phenotypes of circulating monocyte subpopulations as defined by CD14 and CD16 expression[1]. While there are a number of ways to distinguish the monocyte subsets [25, 26], we selected the approach used by Cros et al [6] and by us in our earlier works [1, 27], recognizing that there is not yet a consensus as to how best to phenotype circulating monocytes. In untreated HIV-1 infection, circulating monocytes have greater expression of HLA-DR and CD86 that may reflect in vivo activation and might affect their ability to co-stimulate T cells. Higher expression and density of HLA-DR on monocytes has been reported previously [28]. Interestingly, we found lower CD40 density on traditional monocytes in HIV-1 infection. Earlier work by our group has found diminished induction of CD40L expression on activated (CD38+) CD4 T cells after T cell receptor stimulation [29, 30]. Concurrent lower level CD40 expression on antigen presenting cells may contribute to an impaired CD4 T cell response to antigen in vivo; a recognized complication of HIV-1 infection[31].

We confirmed our previous finding that patrolling monocyte proportions are increased in untreated HIV disease when compared to the patrolling monocyte proportions found in a control population [1]. An increased proportion of CD16+ (inflammatory and patrolling) monocytes in HIV disease has been reported by several groups [32–34], and was recently found to be predictive of greater coronary artery calcium progression in HIV-1-infected patients[34]. We also confirmed previous reports of unaltered CD163 expression on CD14+CD16+ monocytes after administration of ART[35].

Lower CCR2 expression and higher CX3CR1 expression on cryopreserved monocytes from elite controllers and patients on ART with viral suppression has been reported previously [36] while CX3CR1 expression on classical monocytes has been shown previously to be higher in patients with uncontrolled viremia [1]. The expression of CCR2 on monocytes was previously shown to be unaltered in treated HIV disease, similar to what we found after 48 weeks of therapy [27]. We extend these findings by reporting for the first time, significantly lower CCR2 expression on total circulating monocytes, lower CCR2 density on inflammatory monocytes, and lower CX3CR1 expression and density on patrolling monocytes in patients with untreated HIV-1 disease. Lower proportions of CCR2- or CX3CR1-expressing cells may reflect an increased systemic exposure to their ligands, CCL2 or fractalkine respectively, causing impaired monocyte migration. The lowered chemokine receptor expression could also indicate that patient monocytes have already egressed into sites of inflammation and are no longer represented in the blood, leaving behind those monocytes with lower chemokine receptor expression. Importantly, these alterations appear to be abrogated with ART, as shown by the increase in both CCR2 and CX3CR1 expression after ART initiation.

Limitations of this study include a relatively modest sample size and the complexity of dealing with missing samples, due in part to the inconsistent quality and numbers of monocytes present in samples thawed after cryopreservation. We therefore needed to apply statistical methods to deal with missing data (GEE). Also, patients in this study were treated with an integrase-inhibitor based ART regimen. Additionally, as plasma inflammatory profiles have different trajectories after initiation of integrase-based versus non-nucleoside based therapy [37, 38] it is not certain that the findings here will be superimposable after initiation of different ART-based regimens.

It should be noted that our healthy control population is comprised of subjects with defined cardiovascular risk, and may differ from healthy controls utilized in other studies. As our HIV-1-infected patients are also at greater risk for cardiovascular disease [1, 34, 39–42], these controls gave us the opportunity to link the perturbations we observed in circulating monocyte phenotypes to HIV-1 infection itself. On the other hand, as we do not have access to smoking histories of our patients, this remains a limitation of our study. Also, by including controls with defined cardiovascular risk, we may have limited our ability to identify some monocyte phenotypic changes associated with HIV-1 infection and the cardiovascular risks that are its consequence; for example CCR2 expression that did not differ between patients and controls at baseline, but rose with ART.

In summary, we have identified distinct perturbations in circulating monocyte phenotypes in patients with untreated HIV-1 infection: elevated expression of HLA-DR and CD86 that normalized with ART while expression of the chemokine receptors CX3CR1 and CCR2 rose with ART. In contrast, we found lower expression of CD40 in untreated HIV-1 infection which decreased with ART, rather than normalizing. These data demonstrate that cryopreserved monocytes can be used to examine monocyte phenotypes and in HIV-1 infection, and perturbations of circulating monocyte phenotypes tend to improve with administration of suppressive antiretroviral therapy. The role of circulating monocytes in the sustained inflammatory environment of HIV-1 infection warrants further study.

## Supporting Information

S1 Fig CONSORT Flowchart. (TIF)

S1 File CONSORT Checklist. (DOC)

S2 File Study Protocol. (DOC)

S1 Table Mean and Standard Error for frequencies of surface marker expression on total monocytes and monocyte subsets of HIV-1-infected patients before and after initiation of ART. (TIF)

S2 Table Mean and Standard Error for density of surface marker expression on total monocytes and monocyte subsets of HIV-1-infected patients before and after initiation of ART. (TIF)
