# Supplementary figures and images for: Altered Monocyte Phenotype in HIV-1 Infection Tends to Normalize with Integrase-Inhibitor-Based Antiretroviral Therapy
Source: PLoS One. 2015 Oct 2;10(10):e0139474. doi: 10.1371/journal.pone.0139474 (PMC4591977; doi:10.1371/journal.pone.0139474)

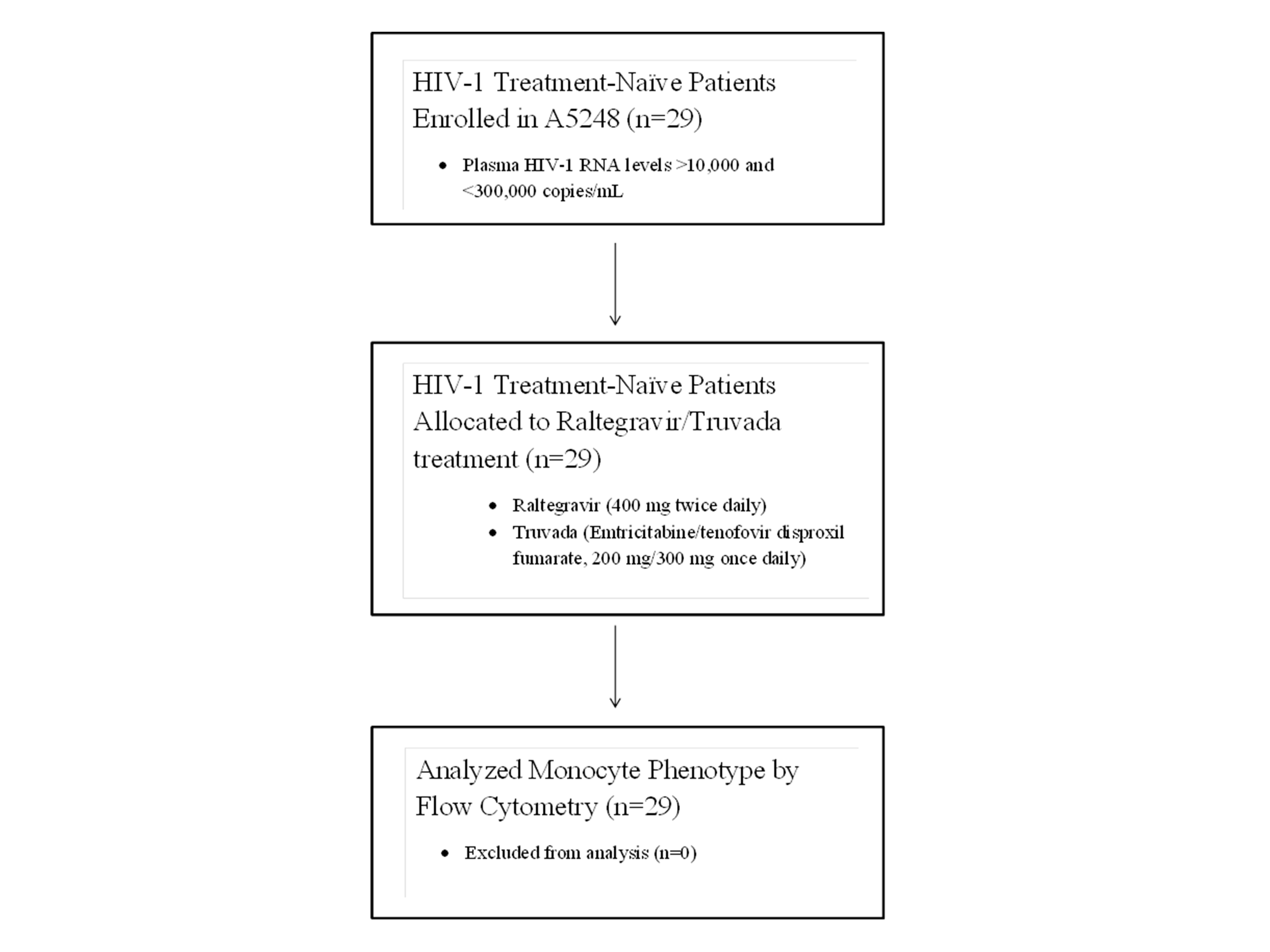

Supplement: S1 Fig — (TIF) [file pone.0139474.s001.tif]

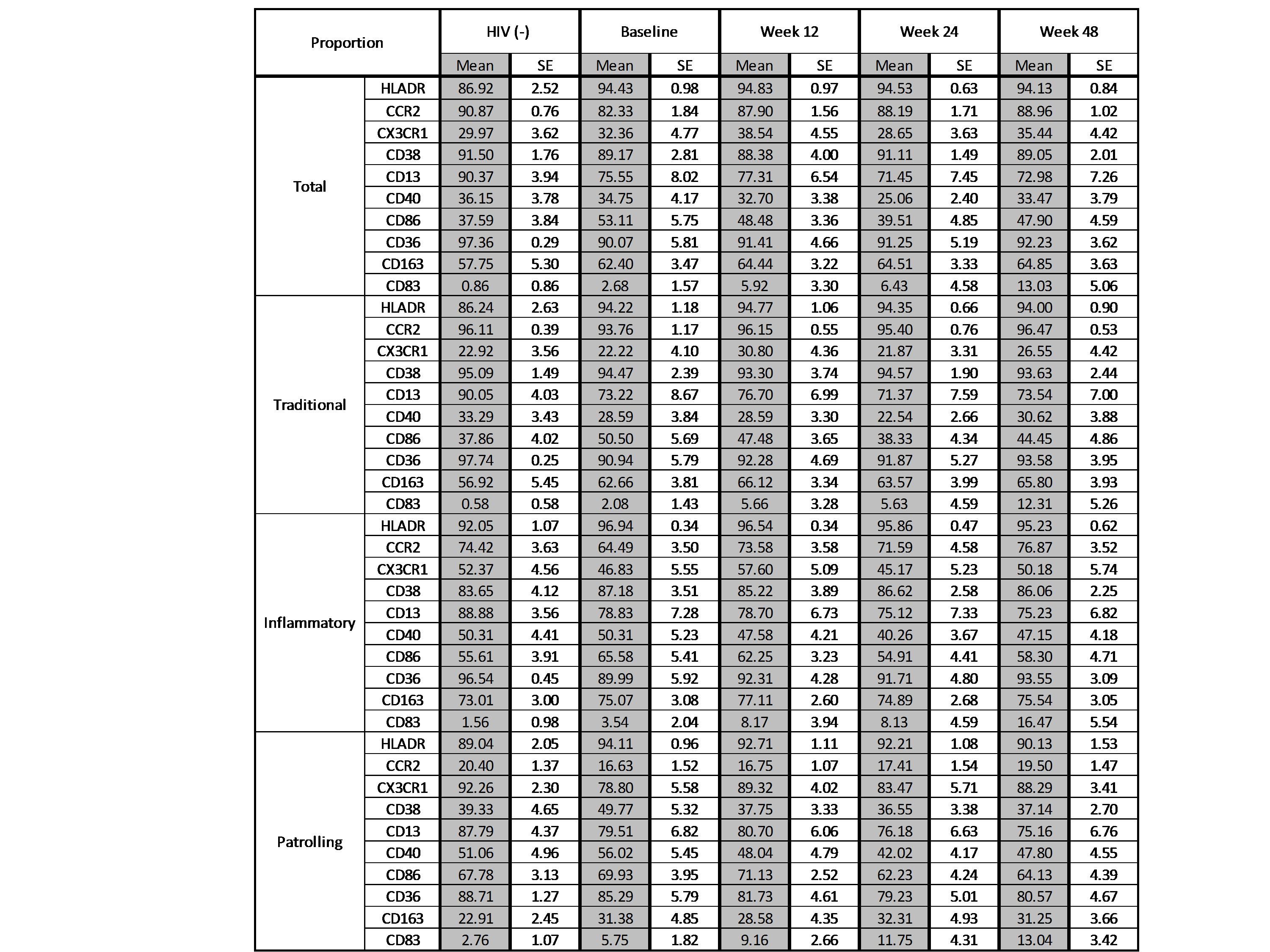

Supplement: S1 Table — (TIF) [file pone.0139474.s004.TIF]

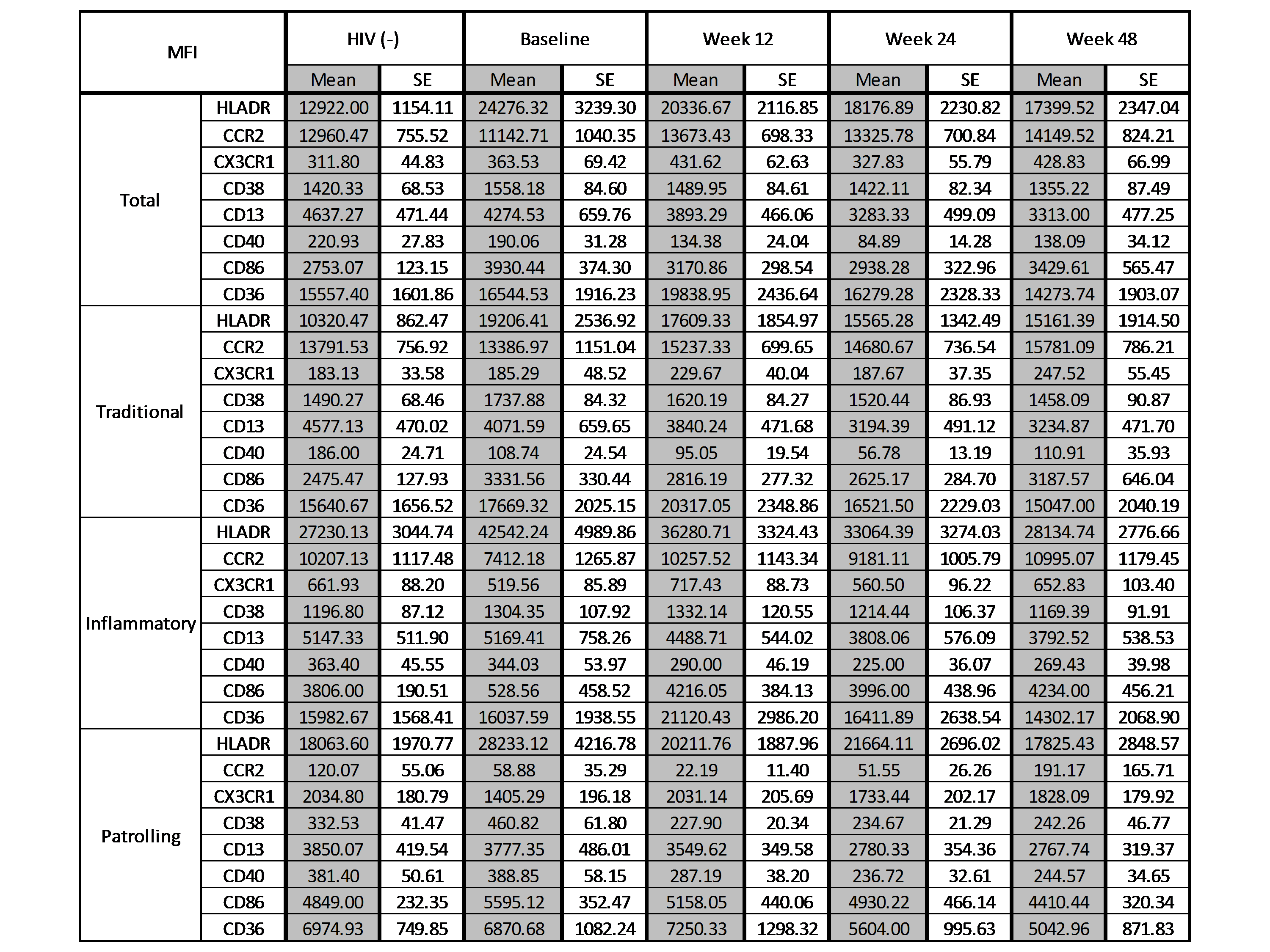

Supplement: S2 Table — (TIF) [file pone.0139474.s005.TIF]
