# Supplementary material for: Altered Monocyte Phenotype in HIV-1 Infection Tends to Normalize with Integrase-Inhibitor-Based Antiretroviral Therapy
Source: PLoS One. 2015 Oct 2;10(10):e0139474. doi: 10.1371/journal.pone.0139474 (PMC4591977; doi:10.1371/journal.pone.0139474)
Supplement: S2 File — (DOC) [file pone.0139474.s003.doc]

**A5248**

**First-Phase Viral Decay Rates in Treatment-Naïve Subjects Initiating Treatment with Raltegravir (MK-0518) and Emtricitabine (FTC)/Tenofovir Disoproxil Fumarate (TDF): A Pilot Study**

**A Multicenter Trial of the AIDS Clinical Trials Group (ACTG)**

**Sponsored by:**

**The National Institute of Allergy**

**and Infectious Diseases**

**Pharmaceutical Support Provided by:**

**Merck and Company**

**IND #**

**The ACTG Translational Research and Drug**

**Development (TRADD) Committee: Cara Wilson, M.D., Chair**

**Protocol Chair: Adriana Andrade, M.D., M.P.H.**

**Protocol Vice Chair: Daniel Kuritzkes, M.D.**

**DAIDS Medical Officer: Sarah Read, M.D.**

**Clinical Trials Specialists: Joelle Touw, B.S.**

**Beatrice Kallungal, B.S.**

**DRAFT Version 0.13**

**November 30, 2007**

CONTENTS

Page

SITES PARTICIPATING IN THE MAIN STUDY [5](#__RefHeading___Toc176316804)

PROTOCOL TEAM ROSTER [6](#__RefHeading___Toc176316805)

STUDY MANAGEMENT [9](#__RefHeading___Toc176316806)

ACRONYMS [11](#__RefHeading___Toc176316807)

SCHEMA [13](#__RefHeading___Toc176316808)

1.0 HYPOTHESIS AND STUDY OBJECTIVES [14](#__RefHeading___Toc176316809)

1.1 Hypothesis [14](#__RefHeading___Toc176316810)

1.2 Primary Objective [14](#__RefHeading___Toc176316811)

1.3 Secondary Objectives [14](#__RefHeading___Toc176316812)

2.0 INTRODUCTION [15](#__RefHeading___Toc176316813)

2.1 Background and Rationale [15](#__RefHeading___Toc176316814)

3.0 STUDY DESIGN [22](#__RefHeading___Toc176316816)

4.0 SELECTION AND ENROLLMENT OF SUBJECTS [24](#__RefHeading___Toc176316817)

4.1 Inclusion Criteria [24](#__RefHeading___Toc176316818)

4.2 Exclusion Criteria [25](#__RefHeading___Toc176316819)

4.3 Study Enrollment Procedures [27](#__RefHeading___Toc176316820)

4.4 Coenrollment Guidelines [27](#__RefHeading___Toc176316821)

5.0 STUDY TREATMENT [27](#__RefHeading___Toc176316822)

5.1 Regimens, Administration, and Duration [28](#__RefHeading___Toc176316823)

5.2 Study Product Formulation and Preparation [28](#__RefHeading___Toc176316824)

5.3 Pharmacy: Product Supply, Distribution, and Accountability [28](#__RefHeading___Toc176316825)

5.4 Concomitant Medications [29](#__RefHeading___Toc176316826)

5.5 Adherence Assessment [29](#__RefHeading___Toc176316827)

6.0 CLINICAL AND LABORATORY EVALUATIONS [31](#__RefHeading___Toc176316828)

6.1 Schedule of Events [31](#__RefHeading___Toc176316829)

6.2 Timing of Evaluations [34](#__RefHeading___Toc176316830)

6.3 Instructions for Evaluations [36](#__RefHeading___Toc176316831)

7.0 CLINICAL MANAGEMENT ISSUES [42](#__RefHeading___Toc176316832)

7.1 Grade 1 or 2 Toxicity [42](#__RefHeading___Toc176316833)

7.2 Grade 3 Toxicity [42](#__RefHeading___Toc176316834)

7.3 Grade 4 Toxicity [43](#__RefHeading___Toc176316835)

7.4 Rash [43](#__RefHeading___Toc176316836)

7.5 Nausea/Vomiting [44](#__RefHeading___Toc176316837)

7.6 Diarrhea [44](#__RefHeading___Toc176316838)

7.7 AST/ALT Elevations [44](#__RefHeading___Toc176316839)

7.8 Lactic Acidosis/Symptomatic Hyperlactatemia [45](#__RefHeading___Toc176316840)

7.9 Decreased Creatinine Clearance [45](#__RefHeading___Toc176316841)

7.10 Skin Hyperpigmentation [46](#__RefHeading___Toc176316842)

7.11 Headache [46](#__RefHeading___Toc176316843)

8.0 CRITERIA FOR DISCONTINUATION [46](#__RefHeading___Toc176316844)

8.1 Permanent Treatment Discontinuation [46](#__RefHeading___Toc176316845)

8.2 Premature Study Discontinuation [46](#__RefHeading___Toc176316846)

9.0 STATISTICAL CONSIDERATIONS [47](#__RefHeading___Toc176316847)

9.1 General Design Issues [47](#__RefHeading___Toc176316848)

9.2 Endpoints [47](#__RefHeading___Toc176316849)

9.3 Randomization [49](#__RefHeading___Toc176316850)

9.4 Sample Size and Accrual [49](#__RefHeading___Toc176316851)

9.5 Monitoring [52](#__RefHeading___Toc176316852)

9.6 Analyses [53](#__RefHeading___Toc176316853)

10.0 PHARMACOLOGY PLAN [57](#__RefHeading___Toc176316854)

10.1 Pharmacology Objectives [57](#__RefHeading___Toc176316855)

10.2 Study Design [57](#__RefHeading___Toc176316856)

10.3 Primary and Secondary Data, Modeling, and Data Analysis [57](#__RefHeading___Toc176316857)

10.4 Anticipated Outcomes [58](#__RefHeading___Toc176316858)

11.0 DATA COLLECTION AND MONITORING AND ADVERSE EVENT REPORTING [58](#__RefHeading___Toc176316859)

11.1 Records to Be Kept [58](#__RefHeading___Toc176316860)

11.2 Role of Data Management [58](#__RefHeading___Toc176316861)

11.3 Clinical Site Monitoring and Record Availability [58](#__RefHeading___Toc176316862)

11.4 Expedited Adverse Event Reporting to DAIDS [59](#__RefHeading___Toc176316863)

12.0 HUMAN SUBJECTS [60](#__RefHeading___Toc176316864)

12.1 IRB Review and Informed Consent [60](#__RefHeading___Toc176316865)

12.2 Subject Confidentiality [60](#__RefHeading___Toc176316866)

12.3 Study Discontinuation [60](#__RefHeading___Toc176316867)

13.0 PUBLICATION OF RESEARCH FINDINGS [60](#__RefHeading___Toc176316868)

14.0 BIOHAZARD CONTAINMENT [61](#__RefHeading___Toc176316869)

15.0 REFERENCES [62](#__RefHeading___Toc176316870)

APPENDIX I: Substudy A5249’s INTENSIVE VIRAL DYNAMICS SUBSTUDY OF A5248

APPENDIX II: A5248 SPECIMEN COLLECTION, PROCESSING, AND SHIPMENT

APPENDIX III: A5249s SPECIMEN COLLECTION, PROCESSING, AND SHIPMENT

APPENDIX IV: A5248 SAMPLE INFORMED CONSENT

APPENDIX V: A5249s SAMPLE INFORMED CONSENT

###### SITES PARTICIPATING IN THE MAIN STUDY

A5248 is open to all U.S. clinical trial units (CTUs) and their clinical research sites (CRSs).

###### PROTOCOL TEAM ROSTER

Chair

Adriana Andrade, M.D., M.P.H.

Division of Infectious Diseases

Johns Hopkins University

1830 East Monument Street, Suite 8074

Baltimore, MD 21205

Phone: (410) 614-4036

FAX: (410) 614-9978

E-Mail: [aandrade@jhmi.edu](../../../../../jtouw/Local%20Settings/JTouw/Desktop/aandrade@jhmi.edu)

Vice Chair

Daniel Kuritzkes, M.D.

Brigham and Women’s Hospital

Harvard Medical School

65 Landsdowne Street, Room 447

Cambridge, MA 02139

Phone: (617) 768-8371

FAX: (617) 768-8738

E-Mail: [dkuritzkes@partners.org](mailto:dkuritzkes@partners.org)

DAIDS Medical Officer

Sarah Read, M.D.

HIV Research Branch

TRP, DAIDS, NIAID, NIH

Room 5218

6700-B Rockledge Drive

Bethesda, MD 20892-7624

Phone: (301) 451-2757

FAX: (301) 435-9282

E-Mail: [readsa@niaid.nih.gov](../../../../Local%20Settings/Temporary%20Internet%20Files/Content.IE5/YM9Z6DMK/readsa@niaid.nih.gov)

Clinical Trials Specialists

Joelle Touw, B.S.

ACTG Operations Center

8757 Georgia Avenue, 12th Floor

Silver Spring, MD 20910-3714

Phone: (301) 628-3000

FAX: (301) 628-3302

E-Mail: [jtouw@s-3.com](../../../../../jtouw/Local%20Settings/JTouw/Desktop/jtouw@s-3.com)

Clinical Trials Specialists (cont.)

Beatrice Kallungal, B.S.

ACTG Operations Center

8757 Georgia Avenue, 12th Floor

Silver Spring, MD 20910-3714

Phone: (301) 628-3377

FAX: (301) 628-3302

E-Mail: [bkallungal@s-3.com](mailto:bkallungal@s-3.com)

Statisticians

Susan Rosenkranz, Ph.D.

Statistical and Data Analysis Center

Harvard School of Public Health

900 Commonwealth Avenue, 2nd Floor

Boston, MA 02215

Phone: (617) 632-5915

FAX: (617) 632-2001

E-Mail: [sue@sdac.harvard.edu](mailto:sue@sdac.harvard.edu)

Song Yu, M.S.

Statistical and Data Analysis Center

Harvard School of Public Health

900 Commonwealth Avenue, 2nd Floor

Boston, MA 02215

Phone: (617) 632-2010

FAX: (617) 632-2001

E-Mail: [syu@sdac.harvard.edu](../../../../../jtouw/Local%20Settings/BKallungal/Local%20Settings/Temporary%20Internet%20Files/OLK6F/syu@sdac.harvard.edu)

Data Manager

Barbara Schock, M.S.

Frontier Science & Technology Research Foundation, Inc.

4033 Maple Road

Amherst, NY 14226-1056

Phone: (716) 834-0900x7270

FAX: (716) 834-8432

E-Mail: [schock.barbara@fstrf.org](mailto:schock.barbara@fstrf.org)

DAIDS Pharmacist

Debra Meres, Pharm.D.

Pharmaceutical Affairs Branch

Henry M. Jackson Foundation for the Advancement of Military Medicine

6700-B Rockledge Dr.

Suite 4222

Bethesda, MD 20817

Phone: (301) 451-2775

FAX: (301) 402-1506

E-Mail: [depayne@niaid.nih.gov](../../../../Local%20Settings/Temporary%20Internet%20Files/Content.IE5/YM9Z6DMK/depayne@niaid.nih.gov)

Immunologist

Michael Lederman, M.D.

Case Western Reserve University

University Hospitals of Cleveland

The Foley Building, Room 401-A

2061 Cornell Road

Cleveland, OH 44106-5083

Phone: (216) 844-8786

FAX: (216) 844-5523

E-Mail: [MXL6@case.edu](../../../../Local%20Settings/Temporary%20Internet%20Files/Content.IE5/YM9Z6DMK/MXL6@case.edu)

Virologist

John Mellors, M.D.

Division of Infectious Diseases

University of Pittsburgh Medical Center

Scaife Hall, Suite 818

3550 Terrace Street

Pittsburgh, PA 15261

Phone: (412) 624-8512

FAX: (412) 383-7982

E-Mail: [mellors@dom.pitt.edu](mailto:mellors@dom.pitt.edu)

Pharmacologist

Edward Acosta, Pharm.D.

Department of Pharmacology and Toxicology

Division of Clinical Pharmacology

University of Alabama at Birmingham

1530 3rd Avenue South, VH 116

Birmingham, AL 35294-0019

Phone: (205) 934-2655

FAX: (205) 934-6201

E-Mail: [eacosta@uab.edu](mailto:eacosta@uab.edu)

Investigators

Eric Daar, M.D.

Harbor-UCLA Medical Center

Los Angeles Biomedical Research Institute

1124 W. Carson St., Building N-24

Torrance, CA 90502

Phone: (310) 222-2467

FAX: (310) 533-0447

E-Mail: [edaar@labiomed.org](../../../../../jtouw/Local%20Settings/BKallungal/Local%20Settings/Temporary%20Internet%20Files/OLK6F/edaar@labiomed.org)

Judith Feinberg, M.D.

University of Cincinnati Medical Center

Holmes Hospital

Eden Avenue and Sabin Way

Room 3112

Cincinnati, OH 45267-0405

Phone: (513) 584-5897

FAX: (513) 584-6040

E-Mail: [judith.feinberg@uc.edu](mailto:judith.feinberg@uc.edu)

Charles Flexner, M.D.

Johns Hopkins University Hospital

Osler 524
600 North Wolfe Street
Baltimore, MD 21287-5554
Phone: (410) 955-9712
FAX: (410) 955-9708

E-mail: [flex@jhmi.edu](mailto:flex@jhmi.edu)

Joel E. Gallant, M.D., M.P.H.

Division of Infectious Diseases

Johns Hopkins AIDS Service

Johns Hopkins University School of Medicine

1830 E. Monument Street, #443

Baltimore, MD 21205

Phone: (410) 955-7473

FAX: (410) 614-8099

E-Mail: [jgallant@jhmi.edu](../../../../../jtouw/Local%20Settings/BKallungal/Local%20Settings/Temporary%20Internet%20Files/OLK6F/jgallant@jhmi.edu)

Investigators (Cont.)

Jeffrey Jacobson, M.D.

Division of Infectious Diseases and HIV Medicine

Drexel University College of Medicine

245 N. 15th Street, MS461

Philadelphia, PA 19102

Phone: (215) 762-6555

FAX: (215)-762-3031

E-Mail: [jeffrey.jacobson@drexelmed.edu](../../../../../jtouw/Local%20Settings/BKallungal/Local%20Settings/Temporary%20Internet%20Files/OLK6F/jeffrey.jacobson@drexelmed.edu)

Field Representative

Karen Cavanagh, R.N.

New York Univ. Medical Center

ACTU, C&D Building, Old Bellevue

550 1st Avenue

New York, NY 10016

Phone: (212) 263-6565

FAX: (212) 263-8264

E-Mail: [karen.cavanagh@med.nyu.edu](mailto:karen.cavanagh@med.nyu.edu)

Laboratory Technologists

Daniel Eggers

AIDS Clinical Trials Group Lab 1

Massachusetts General Hospital

65 Landsdowne Street

4th Floor, Room 435

Cambridge, MA 02139

Phone: (617) 768-8374

FAX: (617) 768-8299

E-Mail: [deggers@partners.org](mailto:deggers@partners.org)

David Shugarts, M.A.

Infectious Diseases

University of Colorado Health Sciences Center

4200 E. 9th Avenue, B168

Denver, CO 80262

Phone: (303) 315-1827

FAX: (303) 315-1816

E-Mail: [david.shugarts@uchsc.edu](mailto:david.shugarts@uchsc.edu)

NCAB Representative

Patrick Kramme, D.V.M., Ph.D.

1907 W. Alder Grove Drive

Tucson, AZ 85704

Phone: (520) 668-5298

E-Mail: [patkramme@aol.com](mailto:patkramme@aol.com)

Industry Representative

Randi Leavitt, M.D.

Infectious Diseases

Merck and Company

10 Sentry Pkwy. Mail Stop BL 3-4

Blue Bell, PA 19422

Phone: (484) 344-2672

FAX: (484) 344-7325

E-Mail: [randi_leavitt@merck.com](mailto:randi_leavitt@merck.com)

Laboratory Data Coordinators

Travis Behm, B.S.

Frontier Science and Technology Research Foundation

4033 Maple Road

Amherst, NY 14228

Phone: (716) 834-0900x7377

FAX: (716) 833-0655

E-Mail: [tbehm@fstrf.org](mailto:tbehm@fstrf.org)

Ken Braun, B.A.

Frontier Science & Technology Research Foundation

4033 Maple Road

Amherst, NY 14226

Phone: (716) 834-0900x7220

FAX: (716) 833-8432

E-Mail: [braun@fstrf.org](mailto:braun@fstrf.org)

###### STUDY MANAGEMENT

All questions concerning this protocol should be sent to [actg.teamA5248@fstrf.org](mailto:actg.teamA5248@fstrf.org) via e-mail. The appropriate team member will respond with a "cc" to actg.teamA5248@fstrf.org. A response should generally be received within 24 hours (Monday-Friday).

Protocol E-mail Group

Sites registering to this study must contact the Computer Support Group at the Data Management Center (DMC) via e-mail ([actg.user.support@fstrf.org](mailto:actg.user.support@fstrf.org)) (include the protocol number in the subject line) to have the relevant personnel at the site added to the actg.protA5248 e-mail group as soon as possible. Inclusion in the protocol e-mail group will ensure that sites receive important information about the study during its implementation and conduct.

Clinical Management

For questions concerning entry criteria, toxicity management, concomitant medications, and coenrollment, contact the protocol Chair/Vice Chair. Send an e-mail message to actg.teamA5248@fstrf.org (ATTN: Adriana Andrade, M.D., M.P.H. and Daniel Kuritzkes, M.D.). Include the protocol number, patient identification number (PID), and a brief relevant history.

Laboratory

For questions specifically related to immunologic, virologic, or pharmacologic laboratory tests, contact the protocol Immunologist, Virologist, or Pharmacologist. Send an e-mail message to actg.teamA5248@fstrf.org (ATTN: Michael Lederman, M.D./ John Mellors, M.D./ Edward Acosta, Pharm.D.).

Data Management

For nonclinical questions about transfers, inclusion/exclusion criteria, case report forms (CRF), the CRF schedule of events, randomization/registration, delinquencies, and other data management issues, contact the Data Manager.

- For transfers, reference the Patient Transfer from Site to Site Standard Operating Procedure 199, and contact Barbara Schock directly.
- For other questions, send an e-mail message to actg.teamA5248@fstrf.org [ATTN: Barbara Schock).
- Include the protocol number, PID, and a detailed question.

Randomization

For randomization questions or problems and study identification number (SID) lists.

Send an e-mail message to [sdac.random.desk@fstrf.org](../../../../Local%20Settings/Temporary%20Internet%20Files/Content.IE5/YM9Z6DMK/sdac.random.desk@fstrf.org) or call the Statistical and Data Analysis Center (SDAC)/DMC Randomization Desk at (716) 898-7301.

Computer and Screen Problems

Contact the SDAC/DMC programmers.

Send an e-mail message to [actg.user.support@fstrf.org](mailto:actg.user.support@fstrf.org) or call (716) 834-0900 x7302.

Protocol Document Questions

For questions concerning the protocol document, contact the Clinical Trials Specialist. Send an e-mail message to [actg.teamA5248@fstrf.org](mailto:actg.teamA5248@fstrf.org) (ATTN: Joelle Touw and Beatrice Kallungal).

Copies of the Protocol

To request hard copies of the protocol, send a message to [ADULT.OPS@fstrf.org](mailto:ACTG.OPS@fstrf.org) (ATTN: Diane Delgado) via e-mail. Electronic copies can be downloaded from the Members area of the ACTG Web site ([http://aactg.s-3.com](http://aactg.s-3.com/)).

Product Package Inserts or Investigator Brochures

To request copies of product package inserts or investigator brochures, contact the DAIDS Regulatory Compliance Center (RCC) at [RIC@tech-res.com](mailto:RIC@tech-res.com) or call (301) 897-1708.

Protocol Registration

Send an e-mail message to [Protocol@tech-res.com](../../../../../jtouw/Local%20Settings/projects/Aactg/PROTDEV/projects/Aactg/PROTDEV/Protocol-Shells-&-Consent-Shells/Protocol-Shell/Protocol@tech-res.com) or call (301) 897-1707.

Study Drug

For questions or problems regarding study drug, dose, supplies, records, and returns, call Debra Meres, Pharm. D., Protocol Pharmacist, at (301) 496-8213.

IND (Investigational New Drug) Number or Questions

Contact the DAIDS RCC at [Regulatory@tech-res.com](../../../../../jtouw/Local%20Settings/projects/kharper/Local%20Settings/Temporary%20Internet%20Files/OLK22/Protocol@tech-res.com) or call (301) 897-1706.

Study Drug Orders

Call the Clinical Research Products Management Center (CRPMC) at (301) 294-0741.

Expedited Adverse Event (EAE) Reporting/Questions

Contact DAIDS through the RCC Safety Office at [**RCCSafetyOffice**](../../../../../jtouw/Local%20Settings/projects/Aactg/PROJECTS/Aactg/PROTDEV/Protocol-Shells-&-Consent-Shells/Protocol-Shell/ProtShell.doc)[**@tech-res.com**](../../../../../jtouw/Local%20Settings/projects/Aactg/projects/Aactg/PROTDEV/projects/Aactg/PROTDEV/Protocol-Shells-&-Consent-Shells/Protocol-Shell/SafetyOffice@tech-res.com) or call 1-800-537-9979 or 301-897-1709; or fax 1-800-275-7619 or 301-897-1710.

Phone Calls

Any phone calls must be documented by e-mail to [**actg.teamA5248@fstrf.org**](mailto:actg.teamA5248@fstrf.org). This will be the site’s responsibility.

Protocol-Specific Web Page

Additional information concerning study management of ACTG studies can be found on the ACTG Web page.

###### ACRONYMS

3TC lamivudine

ABC abacavir

ACTG AIDS Clinical Trials Group

AE adverse event

AER adverse event report

AIDS acquired immunodeficiency syndrome

ALT alanine aminotransferase (SGPT)

ANC absolute neutrophil count

ART antiretroviral therapy

ARV antiretroviral

AST aspartate aminotransferase (SGOT)

AT as-treated

AUC area under the curve

β-HCG beta-human chorionic gonadotropin

BID twice daily

BUN blood urea nitrogen

CI confidence interval

CK creatine kinase

CLIA Clinical Laboratory Improvement Amendments

Cmax maximum concentration

Cmin minimum concentration

CM central memory

CNS central nervous system

CrCl creatinine clearance

CRF case report form

CRPMC Clinical Research Products Management Center

CRS clinical trial site

CTU clinical trial unit

CV coefficient of variation

DAIDS Division of AIDS

DMC Data Management Center

EAE expedited adverse event

EBE empirical Bayes estimate

EFV efavirenz

ELISA enzyme-linked immunosorbent assay

FDA Food and Drug Administration

FDC fixed dose combination

FTC emtricitabine

GCRC General Clinical Research Center

G-CSF granulocyte colony-stimulating factor

HAART highly active antiretroviral therapy

HBM human biological materials

HBV hepatitis B virus

HIV human immunodeficiency virus

HSR hypersensitivity reaction

IATA International Air Transport Association

ICxx inhibitory concentration (where xx equals the percent inhibition)

IND investigational new drug

IRB institutional review board

ITT intent-to-treat

IUD intrauterine device

LDH lactate dehydrogenase

LDMS laboratory data management system

LFT liver function test

LLD lower limit of detection

LPV lopinavir

LPV/r lopinavir/ritonavir, LPV/RTV

LTR long terminal repeat

NIAID National Institute of Allergy and Infectious Diseases

NNRTI non-nucleoside reverse transcriptase inhibitor

NRTI nucleoside reverse transcriptase inhibitor

OBT optimized background therapy

OHRP Office for Human Research Protections (formerly OPRR)

OI opportunistic infection

PBMC peripheral blood mononuclear cell

PCP *Pneumocystis jiroveci* pneumonia

PCR polymerase chain reaction

Pgp p-glycoprotein

PI protease inhibitor

PID patient identification number

PK pharmacokinetic

PO orally

QD once daily

RCC Regulatory Compliance Center

RTV ritonavir

SD standard deviation

SDAC Statistical and Data Analysis Center

SGOT serum glutamic-oxaloacetic transaminase (AST)

SGPT serum glutamic-pyruvic transaminase (ALT)

SID study identification number

TDF tenofovir disoproxil fumarate

TLR toll-like receptor

TRADD Translational Research and Drug Development Committee

ULN upper limit of normal

VID visit identifier

WBC white blood cell

ZDV zidovudine

###### SCHEMA

A5248

First-Phase Viral Decay Rates in Treatment-Naïve Subjects Initiating Treatment with Raltegravir (MK-0518) and Emtricitabine (FTC)/ Tenofovir Disoproxil Fumarate (TDF): A Pilot Study

DESIGN This is a prospective, open-label, limited-site, 72-week pilot study in HIV-1-infected antiretroviral (ARV)-naïve subjects with any CD4+ T-cell count. The goal of the study is to estimate the first-phase viral-decay rate in treatment-naïve subjects receiving raltegravir and FTC/TDF.

DURATION 72 weeks.

SAMPLE SIZE 34 subjects.

POPULATION HIV-1-infected, ARV-drug naïve men and women ≥ 18 years of age with plasma HIV-1 RNA levels between 10,000 and 300,000 copies/mL and any CD4+ T-cell count. Subjects will be ineligible if the screening genotype shows the existence of major nucleoside reverse transcriptase inhibitor (NRTI), non-nucleoside reverse transcriptase inhibitor (NNRTI), or protease inhibitor (PI) resistance mutations.

REGIMEN Raltegravir (400 mg orally [PO] twice daily [BID])+ FTC/TDF (300 mg/200 mg po once daily [QD]).

Raltegravir will be provided by the study. FTC/TDF will not be provided by the study.

SUBSTUDYA5249s: Intensive Viral Dynamics Substudy of A5248 (refer to Appendix I)

1.0 HYPOTHESIS AND STUDY OBJECTIVES

## 1.1 Hypotheses

1.1.1 In treatment-naïve subjects starting a potent ARV regimen consisting of raltegravir and TDF/ FTC, plasma HIV-1 RNA levels will exhibit steeper declines over the first 14 days of treatment compared to the declines previously seen in subjects receiving the three-drug combinations used in the A5160s and A5166s protocols.

1.1.2 Subjects with more rapid first-phase decay (larger d1) will exhibit poorer CD4+ T-cell restoration, as evidenced by: (a) smaller increases from baseline in CD4+ T-cell counts on day 14, and (b) smaller increases from baseline in CD4+ T-cell counts at week 24.

1.1.3 Subjects with more rapid turnover of central memory T-cells at baseline, will exhibit poorer CD4+ T-cell restoration, as evidenced by: (a) smaller increases from baseline in CD4+ T-cell counts on day 14, and (b) smaller increases from baseline in CD4+ T-cell counts at week 24. Speed of turnover of central memory T-cells will represented by the proportion of CD45RA-/CD27+ cells (CD4+ or CD8+ ) that: (c) are in cell cycle (Ki-67+) and/or (d) express one or both activation markers CD38, HLA-DR, as measured at baseline.

1.1.4 The rate of absolute change (slope) in proportions of activated central memory (CM) (CD45RA-CD27+) CD4+ and CD8+ T-cells between baseline and inflection point will correlate inversely with the magnitude of CM and total CD4+ T-cell restoration at days 14 and week 24.

1.1.5 Subjects with more rapid first-phase decay will exhibit more rapid turnover (as manifested by proportions of Ki-67+ cells) of CM T-cells at baseline.

1.1.6 Baseline levels of bacterial 16sDNAs that are reflective of the damaged gut mucosal barrier to translocation will correlate with the baseline proportions of activated CM T-cells

1.1.7 Baseline plasma HIV-1 RNA levels will correlate with baseline proportions of activated CM T-cells.

1.1.8 A mathematical model that takes into account both plasma levels of HIV-1 RNA and plasma levels of bacterial 16sDNAs will correlate better with baseline levels of immune activation and will predict better the magnitude of CD4 T-cell restoration (inversely) than will either index alone.

## 1.2 Primary Objective

To estimate the first-phase viral-decay rate in treatment-naïve subjects receiving raltegravir and FTC/TDF.

## 1.3 Secondary Objectives

1.3.1 To compare first phase viral-decay rates for raltegravir and FTC/TDF to first-phase viral-decay rates previously estimated for the superior arms of A5160s and A5166s.

1.3.2 To estimate the second and third-phase viral decay rates in treatment-naive

subjects receiving raltegravir and FTC/TDF, and to estimate the times at which subjects' decay rates transition from first to second phase, and from second to third phase.

1.3.3 To determine the proportion of subjects with plasma HIV-1 RNA below the limit of detection, <50 copies/mL, at 24, 48, and 72 weeks after initiation of raltegravir and FTC/TDF.

1.3.4 To evaluate the safety and tolerability of treatment with raltegravir and FTC/TDF.

1.3.5 To evaluate changes in CD4+ T-cell counts 24, 48, and 72 weeks after initiation of raltegravir and FTC/TDF.

1.3.6 To investigate the occurrence of raltegravir and FTC/TDF resistance mutations at virologic failure using very sensitive resistance assay techniques.

1.3.7 To evaluate the relationship of raltegravir and FTC/TDF pharmacokinetic (PK) parameters with virologic and immunologic responses, including day 7 viral load change from baseline and change in CD4+ T-cell counts over the first 14 days of study drugs.

1.3.8 To evaluate adherence by self-report in treatment-naïve subjects receiving raltegravir and FTC/TDF.

1.3.9 To investigate the decay rate in unintegrated proviral DNA in peripheral blood mononuclear cells (PBMCs).

1.3.10 To investigate longer-term viral decay in plasma, from single-copy assay results obtained during weeks 24 to 72.

- - 1. To assess the magnitude and slope of changes in CD4+ T-cell count over the first 14 days of treatment with study drugs.
    2. To examine the relationship between first phase virologic decay, immune activation in defined T-cell maturation subsets and CD4+ T-cell restoration.

# 2.0 INTRODUCTION

## 2.1 Background and Rationale

There are many ARV drugs currently available and in development. Many specific combinations of these agents have been compared in randomized controlled trials. However, given the hundreds of potential drug combinations it is impossible to evaluate or compare all such regimens. Furthermore it is clear that caution must be used with untested combinations based upon unexpectedly high failure rates seen with specific regimens. An example of this was the combination of abacavir (ABC), TDF, and lamivudine (3TC).(1) Other regimens may be active but less active than preferred regimens. Examples include the combination of zidovudine (ZDV), 3TC and ABC compared with efavirenz (EFV)-containing regimens.(2) Since regimens clearly differ in efficacy, and because of the need to continue development of new ARV agents, strategies are needed for the rapid screening of new regimens.

Recent data suggest that early virologic response may in fact be predictive of long-term virologic outcomes. If one assumes that treatment has no direct effect on the death rate of productively infected cells or on the rate at which virus particles are removed from plasma, then differences in first-phase decay rates would reflect differences in the extent to which a treatment deviates from ideal (perfect) efficacy.(3) This realization has led to the generation of viral dynamics models and has provided a theoretical rationale for using first-phase decay rates to compare the short-term activity of various regimens.(4-9) For example, a viral dynamic substudy of A5095, A5166s, showed that consistent with the long-term efficacy data, phase 1 decay was indeed slower with the triple NRTI combination than with the EFV-containing regimens.(10) This was further supported in recent analyses of A5142, which compared three regimens. Each proved to be highly efficacious; however, virologic outcomes were better in those who received EFV, either with 2 NRTIs or lopinavir/ritonavir (LPV/r), compared with those treated with LPV/r plus 2 NRTIs.(11) The viral dynamic substudy, A5160s, demonstrated faster phase 1 viral decay in those receiving EFV. It was further shown that the carefully performed assessment of phase 1 decay using frequent virologic measures correlated with the change in plasma HIV-1 RNA seen from baseline to day 7. This allowed for analyses to be performed on all subjects in the parent study which confirmed the observation that the initial change in plasma HIV-1 RNA during the first week of therapy correlated with virologic outcomes at 24, 48, and 96 weeks.(12) Together these studies suggest that defining phase 1 decay through carefully performed studies of viral dynamics may be a useful tool for assessing the likely outcome of long-term treatment. While this is unlikely to replace formal long-term clinical trials it may prove to be a useful screening tool in order to define which combinations should be studied further.

A novel strand transfer HIV-1 integrase inhibitor under development by Merck (MK-0518, raltegravir) has been shown in several trials to be highly efficacious.(13-15) Given these findings, we now propose a pilot study to estimate the rate of suppression of plasma HIV-1 RNA to <50 copies/mL and the phase 1 viral decay rate in treatment-naïve subjects receiving raltegravir plus FTC/TDF. We hypothesize that treatment with this potent regimen will lead to faster clearance of virus compared with the three-drug combinations used in ACTG studies A5095 and A5142, both of which included viral dynamics substudies in which first-phase viral decay rates were estimated for each arm. Results of the present study will help generate hypotheses for future testing regarding possible mechanisms to account for any observed differences in viral decay rates.

Subjects with baseline resistance mutations to NNRTIs or PIs will be excluded because the resistance mutations may be markers of prior exposure or resistance to the NRTIs (FTC/TDF) that are being used in A5248. Such NRTI resistance could have been transmitted or selected by prior therapy and be missed by standard genotyping in patients off therapy.

Evaluating the Effects of Raltegravir on the Latent Reservoir

In conjunction with the study of plasma viral RNA decay, a secondary aim of this study is to determine the effect of raltegravir on the decay of PBMC-associated HIV-1 DNA. The covalent integration of proviral DNA into the host genome is responsible for the long-term persistence of HIV-1 infection despite potent ARV therapy (ART) through the establishment of treatment-resistant viral reservoir in latent CD4+ cells. As with plasma viral RNA levels, total HIV-1 DNA levels show an early and fast decay after initiation of effective therapy, subsequently followed by a slower decay. The rate of decrease in DNA lags behind the decrease in plasma HIV-1 RNA, and HIV-1 DNA persists in PBMCs despite suppression of HIV-1 RNA to undetectable levels.

After reverse transcription HIV-1 DNA can be integrated in the nucleus, but the majority remains in the cytoplasm as linear forms and are not integrated. The estimated half-life of unintegrated DNA is on the order of days, while integrated proviral DNA persists for the lifespan of the host cell.(16;17)  In viremic patients the majority of the total HIV-1 DNA detected is in one of the two unintegrated forms, linear or long-terminal repeat (LTR) circles. The latter are circularized DNA with either one or two LTRs from recombination or ligation of the LTRs. LTR circles constitute about 10% of the intracellular HIV-1 DNA, and although they are also unstable, they persist longer than the linear nonintegrated DNA.

Standard polymerase chain reaction (PCR) does not differentiate between integrated and unintegrated forms of HIV-1 DNA. Quantitative methods to differentiate these different forms of cell-associated DNA have been developed and are suitable for the purposes of this study. In addition to the study of viral RNA decay in plasma we propose to measure the shifts in the different forms of cell-associated HIV-1 DNA after initiation of raltegravir. In the presence of raltegravir, unintegrated HIV DNA becomes a substrate for cellular repair mechanisms that form circular byproducts known as LTR circles. Studies have shown that LTR circles are increased in the presence of integrase inhibitors, and their absolute number corresponds to the potency or efficacy of these drugs.(18;19)

We aim to quantify the total cell-associated DNA, LTR circular DNA and lastly, integrated proviral DNA during the viral RNA decline phase and at viral suppression.(16;20;21) We hypothesize that in the presence of raltegravir total cell-associated HIV-1 DNA will decrease rapidly based on the drug’s inhibitory mechanism at the integration step of the viral lifecycle, but there will be a slower decrease in the integrated proviral DNA and LTR circles. The amount of LTR circles will initially increase while the pathway of integrated DNA is being blocked, followed by a decline to very low or undetectable levels.

Assessing the Association Between Viral Dynamics and Cellular Restoration

In earlier work, we found that the first-phase decay in plasma viremia after application of an ART regimen consisting of ritonavir (RTV)/ZDV/3TC predicted inversely the magnitude of CD4+ T-cell restoration.(22) Since one component of the interindividual variability in virologic decay is the turnover of activated virus infected cells, we propose that this magnitude of immune activation (intrinsic activation level) predicts the intensity of cellular activation and death in lymphoid tissues that is central to T-cell turnover and death in chronic HIV infection. In this model, with application of ART, there is rapid redistribution of sequestered T-cells from lymphoid tissue to the periphery, and subjects with greater “intrinsic activation” states have proportionally more activated and dying memory cells that do not survive in circulation.

ACTG 5248, therefore, will also examine the relationship between first-phase virology decay and cellular restoration and will explore the relationships among immune activation, virologic decay rates and cellular response. We propose that the rapid turnover of CM T-cells is a fundamental determinant of CD4+ T-cell losses in chronic HIV infection.(23;24) We will therefore examine the activation state and cycling of different T-cell maturation phenotypes in this study to test the hypothesis that it is the cycling of phenotypically defined CM CD4+ T-cells that predicts inversely the magnitude of cellular restoration in ART-treated persons with chronic HIV infection. Thus, using cryopreserved PBMCs obtained over the course of this study, we will track the proportion of phenotypically defined naïve (CD45RA+/CD27+), CM (CD45RA-/CD27+) and effector memory (CD45RA-/CD27-) CD4+ and CD8+ T-cells that are in cell cycle (Ki-67+) or express the activation markers CD38 and HLA-DR.

What is driving these cells into cycle is not clear. We are now entertaining the hypotheses that they may be driven into cycle by exposure to microbial toll-like receptor (TLR) agonists, both gut derived(25) and HIV-derived(26) or that they may be driven into cycle by the elevated levels of common gamma chain cytokines in the HIV-infected lymph node.(27) We are therefore attempting to identify the “signature” of CM T-cells that have been activated in vitro by these stimuli. When these studies are completed, we will apply the defined signature patterns retrospectively to these samples to ascertain how these signatures decay and to see if these signatures invoke one driver or the other or both.

In recently published work, Brenchley et al. found evidence for translocation of microbial products from the gut in chronic HIV infection.(25) This was manifested by increased levels of bacterial lipopolysaccharide in subject plasmas. More recently, using degenerate primers and probes, PCR-based assays for bacterial DNAs encoding bacterial 16s ribosomal DNAs have been developed by this group and show higher levels of circulating bacterial DNA in HIV-infected patients versus uninfected controls, especially before administration of ART. (*Jiang et al. submitted to CROI 08*)  In additional work Funderburg et al. *(submitted*) found that each of 8 different microbial TLR agonists can induce the entry into cell cycle and death of CD4+ central memory T-cells in vitro. We propose to monitor the plasma levels of bacterial 16sDNAs in subjects initiating ART in ACTG 5248 to define the decay of these elements from plasma, and to determine their relationship with central memory T-cell activation and turnover in HIV infection. We hypothesize that these microbial TLR agonists drive CM T-cell activation and death in vivo and propose to utilize the data from this study to establish a model that defines this.

Relationship Between Pharmacologic Delay and Viral Dynamics

Some of the most interesting data from viral dynamic studies come from examination of the very early decay phase, as defined by Perelson and colleagues.(3) In a viral dynamic study in 5 subjects receiving RTV monotherapy, Perelson’s group measured viral load intensively over the first several days. In addition to days 0, 2, and 7, plasma HIV-1 RNA was measured at hours 2, 4, 6, 12, 18, 24, 30, 36, and 42; and days 3, 4, 5, and 6. Estimates of the pharmacologic delay, the time lag between first dose of study drug and the onset of decline in virus load, were made, and were longer than the estimated PK delay (i.e., the delay due to drug absorption, distribution, and cell/compartment penetration). This additional delay was felt to be a consequence of the mechanism of action of PIs, which render newly produced virus noninfectious, but which inhibit neither the production of virions from already infected cells, nor the infection of new cells by previously produced virus. This last process is the target of the ARV class of integrase inhibitors and, heuristically, the successful inhibition of this upstream step should lead to earlier reduction of plasma HIV-1 RNA. In a small study of subjects initiating RTV monotherapy, Perelson and colleagues estimated this pharmacologic delay to be 1.2 ± 0.1 days.(3) The A5248 protocol will estimate the effects of pharmacologic delay on the onset of decline in virus load, and will investigate whether raltegravir PKs provide a mechanistic explanation for the fast viral decay that has been observed with this integrase inhibitor.

A critical secondary objective of the protocol is to define the 2nd and 3rd (and possibly later) phases of viral decay in plasma as well as PBMC compartments, which have not yet been defined in patients on raltegravir-containing regimens. Because A5248 offers a raltegravir-based regimen from the outset, it provides the best opportunity to address this question. Obtaining samples from later time points is essential to calculate decay rates of the proviral DNA pool, and to determine the long-term effects of a raltegravir-containing regimen on persistent, low-level viremia (quantified using the single copy HIV RNA assay). Single copy assay data obtained at later time points through week 72 would be compared with similar data available from the Kaletra 720 and 863 studies conducted by Abbott and analyzed by collaborators at Pittsburgh and the NCI-Frederick HIV Drug Resistance Program.(28;29)

Raltegravir (MK-0518)

Raltegravir is a potent strand transfer inhibitor of HIV-1 integrase. Raltegravir was evaluated in September 2007 and approval was recommended by the FDA for use with treatment-experienced patients with HIV infection. Raltegravir is currently available in the United States through an expanded access program. This drug has been evaluated in vitro as well as in phase I to III trials. In vitro, it has an inhibitory concentration 95 (IC95) of 33 nM in 50% human serum and is metabolized primarily via glucuronidation (UGT1A1). It is not a potent inhibitor or inducer of CYP3A4, and therefore is expected to have limited interactions with other ARV agents. Raltegravir has now been studied in a trial of treatment-naïve individuals(15;30) and two large studies of treatment-experienced subjects.(13;14)

Protocol 004 combined TDF plus 3TC with one of four doses of raltegravir (100, 200, 400, or 600 mg BID) or EFV.(15) In this trial, 85-95% of subjects who received raltegravir for 24 weeks at any of the doses studied achieved plasma HIV-1 RNA levels of <50 copies/mL and reached viral suppression significantly faster than did those receiving EFV. Overall the regimens were well tolerated with only one subject discontinuing treatment because of adverse events (AEs) and this was in a subject on 600 mg of raltegravir BID who developed elevation in transaminase. Drug-related AEs reported in > 5% of subjects on raltegravir included nausea (11%), headache (9%), diarrhea (7%), insomnia (7%), abnormal dreams (6%), and flatulence (6%), most of which were mild to moderate. There were no serious AEs reported in this study.

Two phase III randomized controlled trials of treatment-experienced subjects have been reported. These studies, BENCHMRK 1 and 2 were conducted in Europe, Asia/Pacific, Peru, and North and South America.(13;14) In these studies triple class resistant subjects were randomized 2:1 to raltegravir 400 mg BID or placebo plus optimized background therapy (OBT). Results showed that 77% and 43% of subjects in the raltegravir and placebo arms, respectively, had plasma HIV-1 RNA < 400 copies/mL, with a 2 log10 copy/mL reduction in the raltegravir-treated subjects. CD4+ T-cell response was also significantly higher in the raltegravir than the control group. Overall there was no difference in the clinical or laboratory toxicity between those receiving OBT with raltegravir than with placebo. Drug-related clinical AEs were reported in >5% of subjects receiving raltegravir in BENCHMRK 1 for diarrhea (6.5%) with this occurring in 11% of controls. In BENCHMRK 2 clinical AEs occurring in >5% of raltegravir-treated subjects included diarrhea (12.2% versus 9.2% of controls), nausea (9.1% versus 8.4% of controls), and headache (7.8% compared to 4.2% of controls). Laboratory abnormalities reported in > 5% of the raltegravir-treated subjects in BENCHMRK 1 included LDL>190 mg/dL (6% versus 2.8% controls), triglycerides >750 mg/dL (5.6% versus 2.5% controls), aspartate aminotransferase (AST) between 2.6 and 5.1 times upper limit of normal (ULN) (9.9% compared to 2.5% of controls), and alanine aminotransferase (ALT) >5.1 times ULN (5.6% compared to 2.5% of controls). In contrast, for BENCHMRK 2 such laboratory abnormalities were only seen at >5% of individuals for AST between 2.6 and 5 times ULN (8.6% compared to 7.6% of controls) and ALT 2.6 to 5.0 times ULN (6.5% versus 9.2% of controls).

Viral decay modeling from the Merck 004 study in ARV-naïve subjects revealed that, contrary to expectation, there was not a significant difference in terms of first-phase viral decay in the raltegravir versus EFV arms, possibly in part because of the sparse sampling schedule obtained on days 1, 14, 29, 57, 85. It was also reported that the raltegravir and EFV arms did not differ in second-phase decay rates. Instead, the striking difference appeared to be a longer duration of first-phase decay in the raltegravir arms vs. the EFV arm. Mathematical models suggest that the continued virus generation of the second-phase is due to either (1) new infection via long-lived chronic producer’s infected cells or (2) activation of previously dormant latently infected cells. Data were inconsistent with the model that viral production in the second-phase is from long-lived infected cells.(31)

A cross-sectional analysis of integrase resistance data from the BENCHMRK-1 and 2 studies have been presented. The initial data showed that 32 of 41 subjects tested that experienced virologic failure had new mutations in integrase. These mutations tended to be at either N155H or Q148K/R/H along with other mutations, with the two primary mutations rarely seen together in a single individual. These mutational patterns were said to be similar to those selected for with *in vitro* passage of wild type virus in the presence of raltegravir and to be associated with phenotypic resistance.(13;14) Additional cross-sectional and longitudinal data have been reported from a phase II dose-ranging study of raltegravir in treatment experienced subjects, Protocol 005. In this study 35 of the 38 subjects with virologic failure were found to have mutations in integrase, primarily at Q148H/R/K or N155H, both of which being associated with 10-25-fold reductions in susceptibility. They further demonstrated that with the emergence of additional mutations there was increasing levels of drug resistance.(32) However, interpretation of these data is limited by the fact that much of the second phase of decay for most subjects occurred at plasma HIV-1 RNA levels below the limit of detection of quantification method being used (RT-PCR).

Malignancy (Protocol 004, 005, 018 and 019)

In the double-blind portions of the studies, there were 19 subjects (2.5%) experiencing malignancies that occurred post-randomization in subjects on raltegravir and 5 subjects (1.5%) with malignancies occurring in subjects on a comparator arm. The double-blind portion of studies provides an opportunity to evaluate the risk in subjects receiving raltegravir as well as non-raltegravir containing regimens. It should be noted that the cumulative time at risk (duration of follow-up) for subjects receiving raltegravir is not balanced compared to comparator arms because of study design (3:1 or 4:1 randomization ratios in phase II studies and 2:1 in phase III studies). The time at risk for the raltegravir arms of Protocols 004, 005, 018, and 019, as of July 9, 2007, was 820 patient-years. For the comparator arms, the time at risk was 261 patient-years. Adjustment using patient-years is necessary to address the imbalance in time at risk. The adjusted rates of malignancies per 100 patient-years in the double-blind portions of the studies are 2.32 in the raltegravir arms and 1.92 in the comparator arms. This constitutes a relative risk of 1.209 (95% confidence interval 0.44, 4.14). Based on these data, no specific cancer risk attributable to raltegravir is apparent.

Table 1 lists the types of malignancies seen in the double-blind phase of the studies as of July 9, 2007, which approximate the kinds of malignancies expected in heavily treatment experienced subjects.

Table 1. Summary of Malignancy – Double-Blind Data from Phase II and III

Studies as of 09-Jul-2007

|  | Raltegravir (MK-0518) | | | Comparator Group | | |
| --- | --- | --- | --- | --- | --- | --- |
|  | N=758; 820 PY | | | N=323; 261 PY | | |
|  | n (%)† | Recurrent | Diagnosis ≤ 3 Months‡ | n (%)† | Recurrent | Diagnosis  ≤ 3  Months‡ |
| Subjects with Malignancy | 19 (2.5) | 8/19 | 11/19 | 5 (1.5) | 2/5 | 0/5 |
|  |  |  |  |  |  |  |
| Kaposi’s Sarcoma | 4 (0.5) | 3 | 1 | 0 (0) | - | - |
| Non-Hodgkin’s Lymphoma§ | 3 (0.4) | 1 | 3 | 1 (0.3) | - | - |
| Squamous Cell Carcinoma – Anogenital║ | 5 (0.7) | 2 | 3 | 2 (0.6) | - | - |
| Squamous Cell Carcinoma - Other | 1 (0.1) | - | 1 | 1 (0.3) | - | - |
| Rectal Cancer | 1 (0.1) | - | 1 | 0 (0) | - | - |
| Metastatic Neoplasm NOS | 0 (0) | - | - | 1 (0.3) | 1 |  |
| Hepatocellular Carcinoma | 1 (0.1) | - | 1 | 0 (0) | - | - |
| Non-Melanoma Skin Cancer¶ | 5 (0.7) | 2 | 1 | 1 (0.3) | 1 | - |
| † Crude incidence (100 x n/N).  ‡ Diagnosis of neoplasm occurred within 3 months of initiating study therapy.  § Includes B-cell lymphoma, T-cell lymphoma, and lymphoma – other.  ║ Includes squamous cell carcinoma – anal and squamous cell carcinoma CIS.  ¶ Includes squamous cell cancer – skin and basal cell carcinoma.  NOS = Not otherwise specified.  PY = Patient years of exposure.  Subjects with multiple events may be counted more than once in different terms, but only once in one term. | | | | | | |

Though calculation of relative risk is only possible in double-blind studies with comparator arms, the open-label arms in the raltegravir clinical development program afforded the opportunity to evaluate the rates of malignancies in a population with a longer total time at risk. The number of patient-years of follow-up for Protocols 004, 005, 018, and 019 including the double-blind portions and the open-label portions was 1118 patient-years for the raltegravir arm, constituting an additional 298 patient-years of follow-up as compared to the double-blind portions only. With this additional follow-up, the case rate per 100 patient-years was stable at 2.32, indicating that the patient-year adjusted rate for raltegravir seen in the earlier portions of the study did not increase with additional follow-up for subjects on raltegravir.

In summary, while there have been malignancies reported for subjects on raltegravir, there does not appear to be any difference in case rate with the comparator groups, and there does not appear to be any direct evidence of drug relationship to these events. The study population in which most of these events occurred has highly advanced immunodeficiency and the rates of malignancies observed are within the expected rates for subjects with advanced infection. Furthermore, a variety of cancers was reported, and the specific types were expected in this population. Additionally, many were identified soon after study entry, and several were recurrent, suggesting these were likely to be present at or before the time of study entry. Finally, the rate of malignancies does not appear to increase with additional follow-up in subjects on raltegravir.

Emtricitabine and Tenofovir Disoproxil Fumarate Fixed Dose Combination Tablet (Truvada®)

Gilead Sciences has developed Truvada®, a product containing FTC 200 mg and TDF 300 mg in a fixed dose combination (FDC) tablet formulation. A New Drug Application for the FDC was filed with the U.S. FDA on March 12, 2004, and was approved on August 2, 2004.

Several studies have assessed the safety and efficacy of FTC with TDF, albeit none using FDC. Study M02-418 was a phase III, randomized, open-label, multicenter study designed to compare LPV 800 mg/RTV 200 mg QD vs. LPV 400 mg/RTV 100 mg BID with the background regimen of FTC 200 mg QD and TDF 300 mg QD in ARV-naïve subjects with plasma HIV-1 RNA >1,000 copies/mL.(33) A total of 190 subjects between the ages of 19-75 years were enrolled; 115 to the QD arm and 75 to the BID arm. At week 48, based on the intent-to-treat (ITT) (NC=F) analysis, 70% of subjects in the QD regimen demonstrated plasma HIV-1 RNA <50 copies/mL, compared to 64% of those in the BID group (95% confidence interval [CI]: -7%; 20%). Gastrointestinal AEs were the most common cause for discontinuation. Overall, the most common AEs (> 3%) reported were diarrhea, nausea, and vomiting, with diarrhea being reported significantly higher in the QD group (16% vs. 5%; p=0.04). The most common grade 3/4 laboratory abnormalities (> 3%) reported were increased ALT (> 5 x ULN), AST (> 5 x ULN), triglyceride (> 750 mg/dL), and amylase (> 2 x ULN) levels; no significant differences between the 2 groups were observed.

Study 934 is a phase III, randomized, open-label, multicenter study designed to compare a regimen of EFV with either TDF 300 mg/FTC 200 mg QD or ZDV 300 mg/3TC 150 mg BID as FDC Combivir®.(34) A planned 48-week interim analysis was presented showing that discontinuation occurred more frequently in the ZDV/3TC group (9%) than TDF/FTC (4%), mostly because of AEs such as anemia and nausea. The 48-week data demonstrated that using the time to loss of virologic failure as the primary analysis in which missing or switch is counted as a failure, the proportion of subjects with plasma HIV-1 RNA levels less than 400 copies/mL in an ITT population (n=487) was 84% in the TDF/FTC group compared to 73% in the ZDV/3TC-treated subjects (p=0.002). The proportion of subjects with plasma HIV-1 RNA levels <50 copies/mL was 80% in the TDF/FTC group versus 70% in the ZDV/3TC group (p=0.021). All subjects (after week 8) with confirmed >400 copies/mL of plasma HIV-1 RNA at week 48 or early discontinuation were analyzed for genotypic resistance. Genotype data were limited to 23 subjects on ZDV/3TC and 12 subjects on TDF/FTC and showed mostly M184V/I (3% in ZDV/3TC subjects vs. 1% in TDF/FTC subjects) and/or EFV resistance mutations (7% of ZDV/3TC vs. 4% in TDF/FTC subjects), with no subjects developing the K65R mutation. These results are supported by 96 week data.(35)

Lactic acidosis and severe hepatomegaly with steatosis, including fatal cases have been reported with the use of nucleoside analogues alone or in combination, including FTC, TDF, and other ARVs.

Both FTC and TDF reduce hepatitis B viral (HBV) DNA levels in coinfected subjects.(36;37) Hepatic function should be monitored closely with both clinical and laboratory follow-up for at least several months in subjects who are coinfected with HBV and HIV who discontinue FTC and/or TDF.

In rare cases, hypophosphatemia, proteinuria, glycosuria, and reduced creatinine clearance (CrCl) have been seen, and several cases of renal tubular injury have been reported.(38;39) In study 903, discontinuation for renal toxicity was equally infrequent in the TDF and stavudine treated subjects; although all subjects had normal baseline renal function.(40)

FTC/TDF is designated as FDA pregnancy Category B. For more information concerning FTC/TDF coformulation, please refer to the Truvada® package insert.

# 3.0 STUDY DESIGN

A5248 is a prospective, open-label, multicenter, 72-week pilot study designed to estimate the first-phase viral-decay rate in subjects receiving raltegravir and FTC/TDF. HIV-infected ARV-naïve subjects (defined as no previous ARV treatment at any time prior to entry) with plasma HIV-1 RNA levels between 10,000 and 300,000 copies/mL obtained within 42 days prior to study entry will be enrolled. Subjects will be ineligible if the screening genotype shows the existence of major NRTI, NNRTI, or PI resistance mutations. There will be no CD4+ T-cell count restriction.

Thirty-four subjects will each be followed for 72 weeks. It is anticipated that the 34 subjects will be accrued within 8 months of the study opening to accrual. Subjects will have plasma HIV-1 RNA and CD4+ T-cell count measurements at pre-entry and entry. The geometric and arithmetic means, respectively, of these measurements will be used to establish their baseline values. Subjects will also have plasma HIV-1 RNA samples drawn as per the Schedule of Events.

Real-time genotyping for drug resistance will be performed at screening, if not already available, and at the time of virologic failure. The study will provide all the resistance assays. Subjects will also undergo frequent routine safety monitoring, including hematology, chemistry evaluations, and urinalyses.

Virologic failure will be defined as a confirmed plasma HIV-1 RNA level ≥ 1000 copies/mL at or after 16 weeks and before 24 weeks, or ≥ 200 copies/mL at or after 24 weeks.

When a subject is suspected to have virologic failure, a confirmatory HIV-1 RNA and a plasma sample for real-time plasma HIV-1 RNA genotype will be collected within 14 days. The specimen collected for genotype at that visit will be sent for resistance testing once virologic failure has been confirmed. It is recommended that subjects continue their regimen while genotypic resistance testing is being performed.

Adherence to all study drugs will be monitored by self-report as per the Schedule of Events. All subjects should be provided adherence reinforcement throughout the study, according to local standard practice. Subjects with poor adherence will be provided counseling by the site. Plasma will be collected from all subjects at Day 7 PK analyses (see section 10.0).

Subjects who modify the study regimen or miss doses are expected to stay on study and will be followed; however, they will be replaced as follows: If the subject changes the regimen or misses ≥1 dose(s) in the first 14 days, an additional subject will be accrued. For every three subjects who change regimen or miss ≥2 doses between days 15 and 56, an additional subject will be accrued. Sites should notify the protocol team if subjects modify the study regimen or miss doses. Such subjects will be excluded from as-treated (AT) analyses and included in intent-to-treat (ITT) analyses (see Section 9.6 for details). Between weeks 12 and 72, subjects altering the treatment regimen or missing doses will not be replaced; those who alter the regimen or miss more than 25% of doses will be excluded from AT and included in ITT analyses. Section 9.6 states how replaced subjects will be handled in each analysis.

# 4.0 SELECTION AND ENROLLMENT OF SUBJECTS

## 4.1 Inclusion Criteria

4.1.1 HIV-1 infection, as documented by any licensed enzyme-linked immunosorbent assay (ELISA) test kit and confirmed by Western blot at any time prior to study entry. HIV-1 culture, HIV-1 antigen, plasma HIV-1 RNA, or a second antibody test by a method other than ELISA is acceptable as an alternative confirmatory test.

4.1.2 ARV-drug naïve (defined as no previous ARV treatment at any time prior to study entry).

4.1.3 Screening plasma HIV-1 RNA level between10,000 and 300,000 copies/mL obtained within 42 days prior to study entry by any FDA-approved test for quantifying HIV-1 RNA.

NOTE: Any plasma HIV-1 RNA value obtained locally in a Clinical Laboratory Improvement Amendments (CLIA)-certified laboratory within 42 days prior to study entry can be used as the screening value.

4.1.4 Laboratory values obtained within 42 days prior to entry:

- Absolute neutrophil count (ANC) ≥750/mm3
- Hemoglobin ≥10 g/dL
- Platelets ≥50,000/mm3
- AST (SGOT), ALT (SGPT), and alkaline phosphatase ≤5 x ULN
- Calculated CrCl ≥ 60 mL/min as estimated by the Cockcroft-Gault equation:

For men, (140 – age in years) x (body weight in kg) ÷ (serum creatinine in mg/dL x 72) = CrCl (mL/min)*

*For women, multiply the result by 0.85 = CrCl (mL/min)

(A program to assist in calculations is available on the DMC web site at: [www.fstrf.org](http://www.fstrf.org/))

4.1.5 Negative serum or urine pregnancy test at screening and within 48 hours prior to entry for women with reproductive potential (defined as girls who have reached menarche or women who have not been postmenopausal for at least 24 consecutive months, i.e. who have had menses within the preceding 24 months, or have not undergone surgical sterilization [e.g. hysterectomy, bilateral oophorectomy, or salpingotomy]). The urine test must have a sensitivity of 25-50 mIU/mL.

4.1.6 If participating in sexual activity that could lead to pregnancy, the study subjects must use two forms of contraception as listed below while receiving protocol-specified medications and for 60 days after stopping the medications. At least two of the following methods MUST be used appropriately:

- Condoms (male or female) with or without a spermicidal agent. Condoms are recommended because their appropriate use is the only contraception method effective for preventing HIV transmission.
- Diaphragm or cervical cap with spermicide
- IUD (intrauterine device)
- Hormone-based contraceptive. Some medications alter the metabolism of hormone-based contraceptives. This interaction may make hormone-based contraceptives less effective. Therefore, an alternative or an additional contraception method may be required.

Subjects who are not of reproductive potential (girls who have not reached menarche, women who have been postmenopausal for at least 24 consecutive months, or women who have undergone surgical sterilization, [e.g. hysterectomy, bilateral oophorectomy, or salpingotomy] or men who have documented azoospermia), are eligible without requiring the use of contraceptives. Subject-reported history is acceptable documentation of sterilization, other contraception methods, menopause, and reproductive potential.

4.1.7 Men and women age ≥ 18years.

4.1.8 Ability and willingness of subject or legal guardian/representative to provide informed consent.

## 4.2 Exclusion Criteria

4.2.1Pregnancy or breast-feeding.

4.2.2 Previous treatment with any approved or investigational ARV agents at any time prior to study entry.

4.2.3 HIV-specific immunizations within 6 months prior to study entry.

4.2.4 Immunizations within 30 days prior to study entry.

4.2.5 Known allergy/sensitivity or any hypersensitivity to components of study drugs or their formulation.

4.2.6 Any subject with an acute AIDS-defining opportunistic infection (OI) who is not clinically stable or who has not been on therapy for the OI for at least 30 days prior to study entry. Subjects who have no evidence of active disease and are receiving maintenance therapy for AIDS-related OIs will be eligible.

4.2.7 Treatment within 30 days prior to study entry with immune modulators such as systemic steroids, interleukins, interferons, granulocyte colony-stimulating factor (G-CSF), erythropoietin, or any investigational therapy.

NOTE: Subjects receiving stable physiologic glucocorticoid doses, defined as prednisone ≤ 10 mg/day (or equivalent) as a stable or tapering dose, will be permitted. Subjects receiving corticosteroids for acute therapy for *Pneumocystis jiroveci* pneumonia (PCP) or asthma exacerbation, or receiving a short course (defined as ≤ 2 weeks of pharmacologic glucocorticoid therapy) will be permitted.

4.2.8 Evidence of HIV seroconversion within 6 months prior to study entry.

4.2.9 Serious illness requiring systemic treatment and/or hospitalization until candidate either completes therapy or is clinically stable on therapy, in the opinion of the site investigator, for at least 7 days prior to study entry.

NOTE: Oral candidiasis, vaginal candidiasis, mucocutaneous herpes simplex, and other minor illnesses (as judged by the site investigator) have no restriction.

4.2.10 Substance abuse that, in the opinion of the site investigator, would interfere with adherence to study requirements.

4.2.11 Requirement for any current medications that are prohibited with any study treatment (See section 5.4).

4.2.12 Evidence of any major resistance-associated mutation on any genotype performed prior to study entry or at the time of screening. Any pending resistance testing ordered prior to entry must be available for review by the site investigator prior to enrollment to ensure that there are no exclusionary resistance mutations. In the case of uncertainty, genotypes must be available for review by the protocol core team ([actg.coreteamA5248@fstrf.org](mailto:actg.coreteamA5248@fstrf.org)).

For the screening genotype, previous results verifiable by reports from a local CLIA-certified laboratory are acceptable any time prior to study entry as long as the results are available at entry. Laboratory results to support the CRF should be part of the source documentation. For subjects with a pre-existent genotype, a plasma sample for genotypic resistance test should still be obtained and stored at entry for subsequent use as a baseline.

NOTE: Subjects will be excluded if genotype shows presence of any of the

following resistance mutations:

NRTI: M41L, K65R, D67N, T69D or inserts, K70R, L74V/I, Y115F, Q151M, M184V/I, L210W, T215any, K219Q/E

NNRTI: L100I, K103N, V106A/M, V108I, Y181C/I, Y188C/L/H, G190anyA/S

PI: L24I, D30N, V32I, M46I/L, I47V/A, G48V, I50L/V, F53L, V54V/L/M/A/T/S, G73C/S/T/A, V82A/T/F/S, I84V, N88D/S, L90M

## 4.3 Study Enrollment Procedures

4.3.1 Prior to implementation of this protocol, sites must have the protocol and protocol consent form(s) approved by their local institutional review board (IRB). Protocol documents must be registered with and approved by the DAIDS RCC Protocol Registration Office. Protocol registration must occur before the site can enroll any subjects into the study.

Once a candidate for study entry has been identified, details will be carefully discussed with the subject. The subject will be asked to read and sign the approved protocol consent form.

For subjects from whom a signed informed consent has been obtained, an ACTG Screening Checklist must be entered through the DMC Subject Enrollment System.

4.3.2 Registration

Subjects will be registered to A5248 (according to standard procedures) with the ACTG DMC. For subjects from whom informed consent has been obtained, but who are deemed ineligible or who do not enroll into the initial protocol step, an ACTG Screening Failure Results form must be completed and keyed into the database.

## 4.4 Coenrollment Guidelines

Sites are strongly encouraged to coenroll subjects in A5128. Coenrollment in A5128, “Plan for Obtaining Informed Consent to Use Stored Human Biological Materials (HBM) for Currently Unspecified Analyses,” does not require permission from the A5248protocol chairs. For specific questions and approval for coenrollment in other studies, sites must contact the protocol chairs via e-mail as described in the Study Management section.

# 5.0 STUDY TREATMENT

Study treatment is defined as raltegravir (MK-0518) and emtricitabine/tenofovir disproxil fumarate (FTC/TDF, Truvada®).

## 5.1 Regimens, Administration, and Duration

- - 1. Regimen

For this single-arm study, subjects will not be randomized.

The pharmacist will dispense one bottle of study product at the entry visit (day 0) and weeks 4, 8, 12, 16 and 20. At each of these visits, any remaining tablets will be collected from the subjects before a new bottle of study product is dispensed.

The pharmacist will dispense three bottles of study product at the weeks 24, 36, 48 and 60 visits. The pharmacist must receive a new prescription to ensure that the regimen switch occurs. At each of these visits, any remaining tablets will be collected from the subjects before new bottles of study product are dispensed.

At the week 72 visit, the subject must return any remaining study product.

5.1.2 Administration

Raltegravir

Raltegravir 400 mg tablets will be administered one 400 mg tablet PO BID (800 mg per day), with or without food.

Emtricitabine/tenofovir disoproxil fumarate

Emtricitabine/ tenofovir disoproxil fumarate fixed-dose tablet containing 300 mg of tenofovir disoproxil fumarate and 200 mg of emtricitabine will be administered PO as one tablet QD with or without food.

- - 1. Duration

Subjects will take their assigned study treatment for 72 weeks.

## 5.2 Study Product Formulation and Preparation

5.2.1 Raltegravir (MK-0518) 400 mg tablets must be dispensed in the original bottle with the desiccant provided. Store at room temperature (25°C/77°F). Do not store above 30ºC/86°F and protect from moisture.

5.2.2 Emtricitabine /tenofovir disoproxil fumarate (FTC/ TDF, Truvada®) 200 mg/300 mg co-formulated tablet. Store at room temperature (25°C/77°F); excursions permitted to 15º-30°C (59°-86°F).

## 5.3 Pharmacy: Product Supply, Distribution, and Accountability

5.3.1 Study Product Supply/Distribution

Raltegravir is supplied by Merck and Company will be available through the NIAID CRPMC. The site pharmacist can obtain the study product(s) for this protocol by following the instructions in the manual *Pharmacy Guidelines and Instructions for DAIDS Clinical Trials Networks* in the section Study Product Control.

Emtricitabine/tenofovir disoproxil fumarate required for use on study must be obtained by prescription from subjects’ own providers.

5.3.2 Study Product Accountability

The site pharmacist is required to maintain complete records of all study products received from the NIAID CRPMC and subsequently dispensed. All unused study products must be returned to the NIAID CRPMC (or as otherwise directed by the sponsor) after the study is completed or terminated. The procedures to be followed are provided in the manual *Pharmacy Guidelines and Instructions for DAIDS Clinical Trials Networks* in the section Study Product Control.

## 5.4 Concomitant Medications

Below is a list of selected concomitant medications. This list is only current as of the date of this protocol. Therefore, whenever a concomitant medication or study agent is initiated or a dose changed, investigators must review the concomitant medications’ and study agents' most recent package inserts, Investigator’s Brochures, or updated information from DAIDS to obtain the most current information on drug interactions, contraindications, and precautions.

5.4.1 Required Medications

PCP prophylaxis for CD4+ T-cells <200 cells/mm3 is strongly recommended.

5.4.2 Prohibited Medications

Table 2. Prohibited Medications with Raltegravir (MK-0518)

| Medication Class | Prohibited Medication |
| --- | --- |
| Anticonvulsants | Phenobarbital |
|  | Phenytoin |
| Anti-infectives | Rifampin |

5.4.3 Precautionary Medications

Coadministration of agents with nephrotoxic potential (e.g., amphotericin B, aminoglycosides, cidofovir, acyclovir, ganciclovir, vancomycin) or that are renally excreted with TDF may increase serum drug concentrations of TDF and/or increase the concentrations of the other renally excreted agents. TDF (or FTC/TDF fixed-dose combination) should be avoided in subjects with concurrent or recent use of a nephrotoxic agent, in accordance with the Viread® package insert.

Agents, such as probenecid, that inhibit or compete for elimination via renal tubular secretion may reduce the renal elimination rate of TDF.

Package inserts of ARV drugs and concomitant agents should be referred to whenever a concomitant medication is initiated or dose changed, to avoid drug interaction AEs.

## 5.5 Adherence Assessment

Adherence to all study medications will be assessed by self-report at the intervals indicated in section 6.1, Schedule of Events.

6.0 CLINICAL AND LABORATORY EVALUATIONS

## 6.1 Schedule of Events

Table 3.

| Evaluation | Pre-Study Evaluations | | On-Study Evaluations | | | | | | | | | | | | | | | |
| --- | --- | --- | --- | --- | --- | --- | --- | --- | --- | --- | --- | --- | --- | --- | --- | --- | --- | --- |
| Week | Screening | Pre-Entry  (at least 48 hrs post screening) | Entry  (at least 48 hrs post pre-entry) | 1 | 1 | 2 | 2 | 3 | 4 | 8 | 12 | 16 | 20 | 24 | 36 | 48 | 60 | 72 |
| Day |  |  | 0 | 2 | 7 | 10 | 14 | 21 | 28 | 56 |  |  |  |  |  |  |  | Final Visit |
| Window |  |  |  | +/- 1 Day | | +/- 2 Days | | | +/- 3 Days | | +/- 7 Days | | | | | | | |
| Documentation of HIV | X |  |  |  |  |  |  |  |  |  |  |  |  |  |  |  |  |  |
| Medical History/  Medication History | X |  |  |  |  |  |  |  |  |  |  |  |  |  |  |  |  |  |
| Concomitant Medications |  |  | X |  |  |  |  |  | X | X | X | X | X | X | X | X | X | X |
| Complete Physical Exam |  | X |  |  |  |  |  |  |  |  |  |  |  |  |  |  |  |  |
| Targeted Physical Exam |  |  | X |  |  |  | X | X | X | X | X | X | X | X | X | X | X | X |
| Hematology | X | X | X | X | X | X | X | X | X | X | X | X | X | X | X | X | X | X |
| Chemistry | X |  | X |  |  |  |  |  | X | X | X | X | X | X | X | X | X | X |
| Calculated Creatinine Clearance | X |  | X |  |  |  |  |  | X | X | X | X | X | X | X | X | X | X |
| Liver Function Tests | X |  | X |  |  |  |  |  | X |  | X |  |  | X | X | X | X | X |
| Urinalysis | X |  |  |  |  |  |  |  |  |  |  |  |  | X |  | X |  | X |
| Pregnancy Testing | X |  | X | Whenever pregnancy suspected | | | | | | | | | | | | | | |
| CD4+/CD8+ |  | X | X | X | X | X | X | X | X | X | X | X | X | X | X | X | X | X |
| Advanced Flow Assay |  | X | X | X | X |  | X |  | X | X |  |  |  | X |  | X |  |  |
| **Plasma Bacterial 16s DNA** |  | **X** | **X** | **X** | **X** | **X** | **X** | **X** | **X** | **X** | **X** | **X** | **X** | **X** | **X** | **X** | **X** | **X** |
| Plasma HIV-1 RNA (Real Time) | X | X |  |  |  |  |  |  | X | X | X | X | X | X | X | X | X | X |
| Plasma HIV-1 RNA (Batched) |  |  | X | X | X | X | X | X |  |  |  |  |  |  |  |  |  |  |
| HIV-1 RNA Single Copy Assay  (Batched) |  |  | X | X | X | X | X | X | X | X | X | X | X | X | X | X | X | X |
| Proviral DNA, LTR Circular DNA, Integrated Proviral DNA |  |  | X | X | X | X | X | X | X | X |  |  |  |  |  |  |  | X |
| Genotype | X |  |  |  |  |  |  |  |  |  |  |  |  |  |  |  |  |  |
| Stored Plasma |  |  |  |  |  |  |  |  |  |  |  |  |  | X | X | X | X | X |
| Stored PBMC |  |  | X | X | X | X | X | X | X | X |  |  |  | X | X | X | X | X |
| PK Studies |  |  | X | X | X | X | X | X | X | X | X | X | X | X | X | X | X | X |
| Dispense Study Drug |  |  | X |  |  |  |  |  | X | X | X | X | X | X | X | X | X |  |
| Self-Reported Adherence |  |  |  | X | X | X | X | X | X | X | X | X | X | X | X | X | X | X |

Schedule of Events (Cont.)

Table 4.

| Evaluation | Virologic Failure Confirmation | | Premature Discontinuation of  Study Medications |
| --- | --- | --- | --- |
| Concomitant Medications | X | | X |
| Targeted Physical Exam | X | | X |
| Hematology | X | | X |
| Chemistry | X | | X |
| Calculated Creatinine Clearance | X | | X |
| Liver Function Tests |  | | X |
| Urinalysis |  | | X |
| CD4+/CD8+ | X | | X |
| **Advanced Flow Assay** | **X** | |  |
| **Plasma Bacterial 16s DNA** | **X** | |  |
| Plasma HIV-1 RNA (Real Time) | X | | X |
| Genotype | X | |  |
| Stored Plasma | X | |  |
| Stored PBMC | X | |  |
| Self-reported Adherence | X | X | |

## 6.2 Timing of Evaluations

6.2.1 Screening and Pre-Entry Evaluations

Screening and pre-entry evaluations must occur prior to the subject’s starting any study medications, treatments, or interventions.

Screening

Screening evaluations to determine eligibility must be completed within 42 days prior to study entry unless otherwise specified.

In addition to data being collected on subjects who enroll into the study, demographic, clinical, and laboratory data on screening failures will be captured in a screening log and entered into the ACTG database.

In the event a subject presents with pre-existing documentation of a plasma HIV-1 RNA level between 10,000 and 300,000 copies/mL performed locally in a CLIA-certified laboratory within 42 days prior to study entry, the screening and pre-entry visits may be combined. This value will be the plasma HIV-1 RNA entered into the registration screens. The pre-entry plasma HIV-1 RNA drawn at the combined visit will be recorded on the CRF.

Pre-Entry

Pre-entry evaluations must be completed at least 48 hours after screening evaluations and within 14 days prior to study entry.

6.2.2 On-Study Evaluations

Entry (Week 0)

Entry evaluations must occur at least 48 hours after pre-entry evaluations and completed prior to the initiation of study medications. Subjects must begin treatment on day 0 after study enrollment and after the entry evaluations have been completed.

Post-Entry

After entry, study visits up to day 7 must be scheduled within 1 day of the visit indicated in the schedule of events. Visits scheduled for days 10 and 21 must be scheduled within 2 days, and for days 28 and 56 within 3 days, of the visit indicated in the schedules of events. All visits thereafter must be scheduled within 7 days of the visit indicated in the schedule of events.

Visit to Confirm a Suspected Virologic Failure

Subjects with a plasma HIV-1 RNA level ≥ 1000 copies/mL at or after 16 weeks and before 24 weeks, or ≥ 200 copies/mL at or after 24 weeks, will have a confirmatory plasma HIV-1 RNA test within 14 days after initial plasma HIV-1 RNA sample indicating suspected virologic failure was obtained. Subjects who have their first detectable plasma HIV-1 RNA level at week 72 will not return for a confirmatory visit and will be considered treatment failures.

Subjects who have a confirmed increase in plasma HIV-1 RNA level will report to the clinic to have the final visit evaluations performed and will be removed from the study.

6.2.3 Discontinuation Evaluations

Evaluations for Registered Subjects Who Do Not Start Study Treatment

Subjects who do not begin study treatment should have screening, pre-entry, and entry forms completed and keyed. Beyond the entry visit, no further evaluations are required.

Premature Discontinuation of Study Medications Evaluations

Subjects who discontinue all study medications before the end of the study will come in for a premature discontinuation of study medications visit within 14 days after stopping all drugs. They will be encouraged to continue to attend all study visits for the duration of the study but do not require blood draws for PK studies and stored plasma/PBMCs or completion of self-reported adherence assessment.

Final Study Evaluations/Premature Study Discontinuation Evaluations

All subjects who complete the study (at week 72) or prematurely discontinue participation in the study should report to the clinic to have the final visit evaluations performed (section 6.1).

Pregnancy

Subjects who become pregnant after study entry must discontinue study treatment immediately. Subjects will come in for a premature discontinuation of study medications visit within 14 days after stopping all drugs and will continue to be followed on study/off study treatment as per the section 6.1 but do not require blood draws for PK studies and stored plasma/PBMCs or completion of self-reported adherence assessment. The core team must be notified of any pregnancies that occur for subjects on study ([actg.corea5248@fstrf.org](mailto:actg.corea5248@fstrf.org)).

Pregnancies that occur on study should be reported to The Antiretroviral Pregnancy Registry. More information is available at [www.apregistry.com](http://www.apregistry.com/). Phone: 800-258-4263; Fax: 800-800-1052.

Intrapartum complications and/or pregnancy outcome will be recorded on the CRFs up to week 72 and also be reported to The Antiretroviral Pregnancy Registry. After week 72, intrapartum complications and/or pregnancy outcome will be reported only to The Antiretroviral Pregnancy Registry.

## 6.3 Instructions for Evaluations

All clinical and laboratory information required by this protocol is to be present in the source documents. Sites must refer to the Source Document Guidelines on the ACTG Web site for information about what must be included in the source document: <http://aactg.s-3.com/members/download/final/DSOP/sourcedocv2-801.doc>

All stated evaluations are to be recorded on the CRF and keyed into the database unless otherwise specified.

6.3.1 Documentation of HIV-1

HIV-1 infection, as documented by any licensed ELISA test kit, and confirmed by Western blot at any time prior to study entry. HIV-1 culture, HIV-1 antigen, plasma HIV-1 RNA, or a second antibody test by a method other than ELISA is acceptable as an alternative confirmatory test. HIV-1 documentation is not recorded on a CRF.

6.3.2 Medical History

A medical history will be obtained and must be recorded in the source documents. Report diagnoses identified by the ACTG criteria for clinical events and other diseases on the CRF.

Allergies to any medications and their formulations must be recorded in the source documents and on the CRFs.

6.3.3 Medication History

A medication history must be present in the history and recorded in the source documents:

Table 5. Medication History Table

| Medication Category | Complete History or Timeframe | Record in CRFs |
| --- | --- | --- |
| Immune-based therapy | Complete History | No |
| HIV-1-related vaccines | Within 6 months prior to entry | No |
| Prescription drugs for treatment of OIs | Within 30 days prior to entry | Yes |
| Prescription drugs for prophylaxis of OIs | Within 30 days prior to entry | Yes |
| Other Prescription drugs | Within 30 days prior to entry | Yes |
| Non-prescription drugs | Within 30 days prior to entry | No |
| Complementary and alternative medicines | Within 30 days prior to entry | No |

6.3.4 Concomitant Medications

Record on the CRFs treatment with systemic steroids, interleukins, interferons, G-CSF, erythropoietin, investigational drugs, and study-prohibited medications taken since the last study visit, including actual or estimated start dates and stop dates.

6.3.5 ARV Treatment Modifications

During the study, all modifications to ARV drugs including subject-initiated and/or protocol-mandated interruptions, modifications, and permanent discontinuation of treatment will be recorded on the CRFs at each visit. Subject-initiated and protocol-mandated interruptions of study medications include both inadvertent and deliberate interruptions of ≥1 dose(s), from entry to day 14, ≥2 doses from days 15 to 56, and >7 cumulative days thereafter.

6.3.6 Clinical Assessments

Complete Physical Exam

A complete physical examination is required at pre-entry and includes an examination of the skin, head, mouth, and neck; auscultation of the chest; cardiac exam; abdominal exam; and examination of the lower extremities for edema*.* The complete physical exam will also include signs and symptoms, diagnoses, vital signs (temperature, pulse, respiration rate*,* and blood pressure), height, and weight.

Targeted Physical Exam

A targeted physical examination is to be driven by any previously identified or new signs or symptoms that the subject has experienced since the last visit. This exam also includes weight, vital signs (temperature, pulse, respiration rate, and blood pressure), and diagnoses at all visits.

Signs and Symptoms

All signs, symptoms, deaths, and toxicities must be documented in the subject’s record. At entry,record on the CRFs all signs/symptoms occurring within 42 days prior to entry. After entry record Grade >3 signs and symptoms on the CRFs.

Any signs or symptoms that lead to a change in study treatment, regardless of grade, must be recorded on the CRFs.

Sites must refer to the Division of AIDS Table of Grading the severity of Adult and Pediatric Adverse Events (DAIDS AE Grading Table). Version 1.0, December 2004, which can be found on the DAIDS RCC Web site: <http://rcc.tech-res.com/tox_tables.htm>.

Diagnoses

Report diagnoses identified by the ACTG criteria for clinical events and other diseases. Refer to the study CRFs for the appropriate appendix used for current ACTG criteria.

6.3.7 Laboratory Evaluations

Record all protocol-required laboratory values, regardless of grade, obtained at screening, pre-entry and entry on the CRFs. After entry, record all laboratory values Grade >3 on the CRFs. Any laboratory values that lead to a change in study treatment, regardless of grade, will be recorded on the CRFs.

Sites must refer to the Division of AIDS Table of Grading the severity of Adult and Pediatric Adverse Events (DAIDS AE Grading Table). Version 1.0, December 2004, which can be found on the DAIDS RCC Web site: <http://rcc.tech-res.com/tox_tables.htm>.

Hematology

As per the Schedule of Events, the following tests are required and performed in real time at the local laboratory: hemoglobin, hematocrit, white blood cell count (WBC), differential WBC, ANC, and platelet count.

NOTE: A hematology panel is not required on days 2, 7, 10, and 14 at sites using a single-platform assay for CD4+/CD8+ T-cell counts.

Blood Chemistries

As per the Schedule of Events, the following tests are required and performed in real time at the local laboratory: electrolytes (Na+, K+, Cl-, HCO3), glucose, creatinine, and blood urea nitrogen (BUN).

Calculated Creatinine Clearance

As per the Schedule of Events, calculated CrCl as estimated by the Cockcroft-Gault equation (Refer to section 4.1.4). This requires the recording of all values of serum creatinine regardless of grade.

Liver Function Tests

As per the Schedule of Events, the following tests are required and performed in real time at the local laboratory: AST (SGOT), lactate dehydrogenase (LDH), ALT (SGPT), and alkaline phosphatase.

Urinalysis

The urinalysis should be completed as per the Schedule of Events and if clinically indicated. Dipstick or microscopic exam may be done. If dipstick results are abnormal, microscopic exam is required.

Pregnancy Test

All women of reproductive potential (who have not reached menopause, undergone hysterectomy, bilateral oophorectomy, or tubal ligation) must have a negative serum or urine beta-human chorionic gonadotropin (β-HCG) pregnancy test result at screening and within 48 hours prior to initiating protocol-specified medications and any time thereafter that pregnancy is suspected. (The urine test must have a sensitivity of 25-50 mIU/mL).

6.3.8 Immunologic Studies

CD4+/CD8+

CD4+/CD8+ T-cell count will be obtained as per the Schedule of Events. Pre-entry and entry measurements must be obtained at two separate time points at least 48 hours apart and no more than 14 days apart. The mean of the pre-entry and entry measurements will be used as the baseline value.

Evaluations for CD4+ and CD8+ T-cell counts should be performed at the same laboratory, if possible, for baseline calculation and throughout the course of the study. The laboratory performing the CD4+ and CD8+ T-cell evaluations must have CLIA certification, or its equivalent. The laboratory also must participate in and be certified for protocol testing by the DAIDS Immunology Quality Assurance (IQA) Program.

Because of the diurnal variation in CD4+ and CD8+ T-cell counts, determinations for individual subjects should be obtained consistently in either the morning or the afternoon throughout the study.

NOTE: For **all** laboratories, a separate WBC with differential is required if a complete blood count is not performed on the same day. This will require an additional 5mL EDTA tube to be collected on the same day as the CD4+ and CD8+ T-cells sample and sent to the local clinical lab.

CD4+ and CD8+ T-cell counts (absolute and percentage) must be recorded in the source document and on the CRFs.

Advanced Flow Assay

Blood will be collected and PBMCs cryopreserved for advanced flow testing. Refer to Appendix II for details concerning processing and shipping of the specimens.

NOTE: The advanced flow assay also requires CD4+/CD8+ and WBC with differential from a sample obtained at the same time.

**Plasma Bacterial 16s DNA**

**Bacterial 16s DNA levels will be obtained as per the Schedule of Events. Refer to Appendix II for details concerning processing and shipping of the specimens.**

6.3.9 Virologic Studies

Plasma HIV-1 RNA (real-time and batched)

Screening plasma HIV-1 RNA level should be obtained within 42 days prior to study entry by any FDA-approved test for quantifying HIV-1 RNA. Screening plasma HIV-1 RNA may be obtained locally using any standard assay. The testing laboratory should have a CLIA certification or its equivalent. Any plasma HIV-1 RNA value performed locally in a CLIA-certified laboratory within 42 days prior to study entry can be used as the screening value.

Plasma HIV-1 RNA samples obtained at entry through day 21 should be batched and shipped according to Appendix II. Plasma HIV-1 RNA for all other study time points will be tested in real time and shipped according to Appendix II.

Eligibility will be determined based on the screening HIV-1 RNA value. The baseline value will be the geometric mean of the pre-entry and entry determinations, which must have been obtained at least 48 hours but no more than 14 days apart. Plasma HIV-1 RNA quantitation after screening will be measured by Johns Hopkins University Virology Specialty Laboratory using the current, approved UltraSensitive Roche Amplicor HIV-1 Monitor assay.

HIV-1 RNA Single Copy Assay (batched)

Plasma HIV-1 RNA will also be measured using the single-copy assay for HIV-1 RNA samples with <50 copies/mL.  Samples will be collected as per the Schedule of Events and processed and shipped according to Appendix II. The single-copy assays will be done as batched tests, not in real time.

Proviral DNA, LTR Circular DNA, Integrated Proviral DNA

Total cell-associated proviral DNA, LTR circular DNA, and integrated proviral DNA will be measured from PBMCs. Samples will be collected as per the Schedule of Events and processed and shipped according to Appendix II.

NOTE: Proviral DNA, LTR circular DNA, and integrated proviral DNA

samples should only be collected at the week 72/final study visit if virologic failure has been confirmed.

Genotype

Genotypic resistance testing is required prior to study entry. For the screening genotype, previous results verifiable by reports from a local CLIA-certified laboratory are acceptable any time prior to study entry as long as the results are available at entry. Genotype results should be part of the source documentation.

NOTE: For subjects with a pre-existent genotype, a plasma sample for genotypic

resistance testing should be obtained and stored at entry for subsequent use as a baseline.

At or after Week 16 when a subject is suspected to have virologic failure, a confirmatory plasma HIV-1 RNA and a plasma sample for real-time genotyping will be collected. The specimen collected for genotype at that visit will be sent according to the instructions in Appendix II for resistance testing once virologic failure has been confirmed. Real-time results will be shared with sites.

Stored Plasma and Stored PBMCs

Plasma and PBMCs will be stored at the ACTG repository for future virologic assays. Refer to Appendix II regarding shipping instructions for the stored plasma and stored PBMCs.

NOTE: Stored plasma and stored PBMC samples should not be obtained if a subject is off study treatment, i.e., subject has permanently discontinued all study medications.

6.3.10 PK Studies

Refer to section 10.2 for information pertaining to the PK studies.

6.3.11 Self-Reported Adherence

Adherence to all study medications will be assessed by self-report as outlined in the Schedule of Events.

# 7.0 CLINICAL MANAGEMENT ISSUES

Criteria for subject management, dose interruptions, modifications, and discontinuation or changes in drug treatment will be mandated only for toxicities attributable to raltegravir. Toxicities due to FTC/TDF should be managed according to standard clinical practice, with the exception of sections 7.9 and 7.10, with the goal of maintaining continuous therapy, if possible.

The grading system for drug toxicities is located in the Division of AIDS Table for Grading the Severity of Adult and Pediatric Adverse Events, Version 1.0, December 2004, located at the DAIDS RCC Web Site: <http://rcc.tech-res.com/tox_tables.htm>.

NOTE: The core team must be notified by e-mail regarding toxicities that result in a change in regimen or discontinuation ([actg.coreteama5248@fstrf.org](../../../../Local%20Settings/Temporary%20Internet%20Files/Content.IE5/YM9Z6DMK/actg.coreteama5248@fstrf.org)). Team notification regarding toxicity is especially important during the intensive viral sampling phase in the first 2 weeks of treatment. The protocol does not allow drug substitution for toxicity management.

## 7.1 Grade 1 or 2 Toxicity

Subjects who develop a Grade 1 or 2 AE or toxicity may continue raltegravir. If subjects choose to discontinue raltegravir, the site should complete the premature discontinuation of study medications evaluations, notify the A5248 core team, and encourage the subject to complete safety visits for the study until the toxicity resolves.

## 7.2 Grade 3 Toxicity

If the investigator has compelling evidence that the AE has NOT been caused by raltegravir, dosing may continue. Subjects who develop possible raltegravir-related Grade 3 AE or toxicity, except as stated in the following sections, should have their regimen stopped and the A5248 core team must be consulted. The subject should be reevaluated closely until the AE returns to Grade ≤2, at which time their regimen may be reintroduced at the discretion of the investigator or according to standard practice.

If the same Grade 3 AE recurs within 4 weeks, the regimen must be permanently discontinued. If the same Grade 3 AE recurs after 4 weeks but is not believed to be related to raltegravir, the management scheme outlined above may be repeated.

Subjects experiencing Grade 3 AEs requiring permanent discontinuation of the regimen should be followed closely for resolution of the AE to grade ≤2 and the core team must be consulted.

## 7.3 Grade 4 Toxicity

Subjects who develop a Grade 4 symptomatic AE or toxicity thought to be related to raltegravir will have their regimen discontinued. If the investigator has compelling evidence that the AE has not been caused by raltegravir, dosing may resume when the AE has resolved. Subjects experiencing Grade 4 AEs requiring permanent discontinuation of raltegravir should be followed closely until resolution of the AE to Grade ≤2 and the core team must be consulted.

Subjects with Grade 4 asymptomatic laboratory abnormalities in lipids or creatine kinase (CK) may continue raltegravir.

## 7.4 Rash

7.4.1 Grade 2

Antihistamines, topical corticosteroids, or a brief course of systemic corticosteroids, at the discretion of the site investigator, may be prescribed and subjects may continue all raltegravir. The subject should be advised to contact the physician immediately if there is any worsening of the rash, or if systemic signs or symptoms develop that could be compatible with a hypersensitivity reaction (HSR).

If the rash is considered to be most likely due to concomitant illness or drug, standard management, including discontinuation of the likely causative agent, should be undertaken. If no other causative factor is found after clinical evaluation, the subject should be treated symptomatically until the rash resolves.

7.4.2 Grade 3

If the rash is thought to be related to raltegravir, subjects must discontinue their ARV regimen and the core team must be consulted.

7.4.3 Grade 4

Subjects must discontinue their ARV regimen. The core team should be consulted.

In the event that Grade 2, 3, or 4 rash fails to resolve, increases in severity, is associated with systemic (fever, malaise, nausea) or allergic (e.g., urticaria) symptoms or Grade 3 or 4 liver function test (LFT) elevations, or is associated with exfoliative dermatitis or mucous membrane involvement or erythema, multiforme or suspected Stevens-Johnson syndrome or necrosis requiring surgery, their regimen should be discontinued until symptom resolution. The core team must be consulted.

## 7.5 Nausea/Vomiting

Nausea/vomiting may be treated symptomatically with oral antiemetics or antiemetic suppositories. For Grade ≥ 3 nausea and vomiting thought secondary to raltegravir that fails to improve on antiemetics to Grade ≤ 2, the regimen may be held until Grade ≤ 2. If Grade ≥ 3 nausea and vomiting recurs with reinstitution of raltegravir or persists beyond 14 days despite symptomatic management, then their regimen may be discontinued at the discretion of the investigator in consultation with the core team.

## 7.6 Diarrhea

Symptomatic treatment of any Grade diarrhea with oral antidiarrheal drugs is permitted once infectious causes of diarrhea have been ruled out by the site investigator. If Grade ≥ 3 diarrhea thought to be related to raltegravir persists despite symptomatic management, then the regimen should be held until Grade ≤ 2. If Grade ≥3 diarrhea recurs with reinstitution of raltegravir or persists beyond 14 days despite symptomatic management, then the regimen should be discontinued and the core team consulted.

## 7.7 AST/ALT Elevations

7.7.1 Grade ≤ 3

The regimen may be continued for asymptomatic, isolated Grade ≤ 3 AST or ALT elevations at the discretion of the site investigator. Careful assessments should be done to rule out the use of alcohol, non-raltegravir-related drug toxicity, or viral hepatitis as the cause of the Grade 3 elevation.

For symptomatic Grade 3 elevations of AST or ALT, the ARV regimen should be held until toxicity returns to Grade ≤ 2. If symptomatic Grade 3 elevation recurs on rechallenge, the regimen should be discontinued and the core team must be consulted.

7.7.2 Grade 4

The regimen should be held for confirmed AST or ALT Grade 4 elevations until the toxicity returns to Grade ≤ 2. If Grade 3 elevation in AST or ALT recurs on rechallenge, the ARV regimen should be permanently discontinued and the core team must be consulted.

Any symptomatic Grade 4 ALT or AST elevation should lead to permanent discontinuation of the ARV regimen if it is suspected to be due to the ARVs. The core team must be consulted.

## 7.8 Lactic Acidosis/Symptomatic Hyperlactatemia

Symptomatic hyperlactatemia will be defined as new, otherwise unexplained, and persistent occurrence for >2 weeks of one or more of the following:

- nausea and vomiting
- abdominal pain or gastric discomfort
- abdominal distention
- increased LFTs
- unexplained fatigue
- dyspnea
- motor weakness

plus a confirmed lactate level >2 x ULN.

If the lactate value is >2 x ULN, obtain a confirmatory lactate value as soon as possible, preferably within 1 week and consult the core team. Study treatment should be discontinued immediately if the confirmatory value remains >2 x ULN or if the site is unable to obtain a confirmatory value with 1 week. Determine lactate levels every 4 weeks until the lactate value returns to normal.

Management of symptomatic lactate value <2 x ULN will proceed at the discretion of the local investigator. However, any modification of a subject's ARV regimen should be made in consultation with the protocol team. Since some of the symptoms of hyperlactatemia are vague and may be present in many subjects (e.g., fatigue), repeated lactate determinations are advised.

NOTE: See the ACTG Web site for guidelines for the collection of lactate specimens: **http://aactg:cure@aactg.s-3.com/members/psmet.htm**.

## 7.9 Decreased Creatinine Clearance

If the calculated CrCl is <50mL/min*, it should be confirmed within 1 week. If the calculated CrCl remains <50ml/min, the FTC/TDF must be permanently discontinued and the core team notified.

*Calculate a CrCl from the serum creatinine concentration in mg/dL using the Cockcroft and Gault equation. (Refer to section 4.1.4)

## 7.10 Skin Hyperpigmentation

The development of skin hyperpigmentation, if assessed as being related to FTC, can be followed without modification of therapy, at the discretion of the site investigator.

## 7.11 Headache

Analgesics, antimigraine drugs, muscle relaxants, and other symptomatic treatment should be used at the discretion of the investigator and the subjects may continue on raltegravir. The subject should be advised to contact the physician immediately if there is any worsening of the headache.

If the headache is considered to be most likely due to concomitant illness or drug, standard management, including discontinuation of the likely causative agent, should be undertaken. If no other causative factor is found after clinical evaluation, the subject should be treated symptomatically.

If the headache is thought to be related to raltegravir, the subject should be managed at the discretion of the investigator. If the ARV regimen is discontinued, the core team must be consulted.

# 8.0 CRITERIA FOR DISCONTINUATION

## 8.1 Permanent Treatment Discontinuation

- Drug-related toxicity (see section 7.1 Toxicity).
- Requirement for prohibited concomitant medications (refer to section 5.4).
- Completion of treatment as defined in the protocol.
- Request by subject to terminate treatment.
- Clinical reasons believed life threatening by the physician, even if not addressed in the toxicity section of the protocol.
- Pregnancy or breast-feeding.

## 8.2 Premature Study Discontinuation

- The subject misses 3 consecutive clinic visits.
- Between weeks 12 and 72, subject misses more than 25% of study medications (since the last visit, self-report).
- Request by the subject to withdraw.
- Request of the primary care provider if s/he thinks the study is no longer in the best interest of the subject.
- Subject judged by the investigator to be at significant risk of failing to comply with the provisions of the protocol as to cause harm to self or seriously interfere with the validity of the study results.
- A defined study endpoint reached (virologic failure as defined in section 3.0)*.*
- At the discretion of the ACTG, IRB, Food and Drug Administration (FDA), Office for Human Research Protections (OHRP), NIAID, investigator, or pharmaceutical supporter.

# 9.0 STATISTICAL CONSIDERATIONS

## 9.1 General Design Issues

This is a prospective, single-arm, 72-week pilot study to estimate the first-phase viral decay constant (d1) in treatment-naïve subjects initiating a three-drug regimen consisting of the investigational HIV-1 integrase inhibitor raltegravir and the nucleotide/nucleoside analogue reverse transcriptase inhibitor combination FTC/TDF.

In general, analyses that address mechanisms of pathobiology will be conducted on subjects AT, and analyses of markers for clinical outcome will be ITT. The accrual target of 34 subjects is expected to yield plasma HIV-1 RNA data from 30 subjects for model-fitting, and thus provide a 95% confidence interval around the estimated d1 of no wider than ±0.07, assuming the population mean and standard deviation of d1 for the study regimen are 0.80 and 0.20, respectively. Under these assumptions, with usable data from 30 subjects, there is 98% probability that the CI around the estimated d1 will exclude the value 0.65.

Subjects who modify the study regimen or miss doses are expected to stay on study and be followed; however, they will be replaced as follows: If the subject changes the regimen or misses any dose in the first 14 days, an additional subject will be accrued. For every three subjects who change regimen or miss 2 or more doses between days 15 and 56, an additional subject will be accrued. **(Although subjects dropping out before day 56 will be replaced in this way, the buffer of 4 additional subjects [Section 9.4] is retained to allow for missing results on obtained samples.)** Such subjects will be excluded from AT analyses and included in ITT analyses (see Section 9.6 for details). Between weeks 12 and 72, subjects altering the treatment regimen or missing doses will not be replaced; those who alter the regimen or miss more than 7 cumulative days of doses will be excluded from AT and included in ITT analyses.

The primary objective will be analyzed when plasma HIV-1 RNA results through day 28 are available (as quantified using the standard assay). Specifically, when the last subject to enroll has reached day 21, sites will be asked to send those batched plasma HIV-1 RNA samples that are intended for standard assay, to the assay lab (Johns Hopkins University). When results for these samples and when the real-time results for the day 28 and 56 samples are available, analysis to estimate first and second phase viral decay rates will begin.

## 9.2 Endpoints

9.2.1 Primary Endpoint

Population- and subject-specific first-phase plasma HIV-1 RNA level decay rates d1 (as estimated from biexponential decay models fit to plasma HIV-1 RNA level data through day 56).

9.2.2 Secondary Endpoints

9.2.2.1 Population and subject-specific first-phase plasma HIV-1 RNA level decay rates d1 (as estimated from biexponential decay models fit to plasma HIV-1 RNA level data through day 56).

9.2.2.2 Population and subject-specific second-phase viral load decay rates d2 (as estimated from biexponential decay models fit to viral load data through day 56); population and subject-specific third-phase decay rate d3 as estimated from plasma HIV-1 RNA levels obtained weeks 24 to 72.

9.2.2.3 Proportion of subjects with viral load <50 copies/mL 24, 48, and 72 weeks after initiation of raltegravir and FTC/TDF. If the first detectable viral load corresponds to the sample collected at the last visit, the subject will be counted as detectable (at this visit) without confirmation.

9.2.2.4 Safety and Tolerability

- Incidence of Grade 3 and 4 symptoms and laboratory results (using the DAIDS grading scale) by body system and by type, excluding asymptomatic elevations in lipids or CK.
- Incidence of permanent discontinuation of study treatment for any reason.
- Incidence of permanent discontinuation of study treatment for reasons other than virologic failure.
  - - 1. Change in CD4+ T-cell count from baseline to weeks 24, 48, and 72. The baseline CD4+T-cell count will be the arithmetic mean of the two measurements obtained on different days closest before or on the day of study treatment dispensation (e.g., pre-entry and entry determinations). If only one such measurement is available, then this measurement will be the baseline value.
      2. Raltegravir-, TDF- and FTC-resistance mutations using very sensitive resistance assay measures in subjects with detectable viremia.
      3. Subject-specific estimates of: (a) the PK parameters area under the curve (AUC), minimum concentration (Cmin), and maximum concentration (Cmax) for raltegravir, TDF and FTC; (b) viral load changes on day 7; (c) CD4+ T-cell counts changes over days 0-14 (magnitude of changes and estimated slope). The baseline HIV-1 RNA will be the geometric mean of the two measurements obtained on different days closest before or on the day of study treatment dispensation (e.g., pre-entry and entry determinations). If only one such measurement is available, then this measurement will be the baseline value.
      4. Adherence by self-report on days 2, 7, 10, 14, 21, 28, and 56, and weeks 24, 48, and 72.
      5. Total cell-associated proviral DNA, LTR circular DNA and integrated proviral DNA as measured by an assay currently undergoing validation; and unintegrated linear proviral DNA calculated by subtraction, measured on days 0, 2, 7, 10, 14, 21, 28, and 56 and at the time of virologic failure.
      6. Plasma HIV-1 RNA levels collected on day 2 through week 72, as quantified by the single-copy assay.
      7. CD4+ T-cell counts measured on days 0, 2, 7, 10, and 24.
      8. Subject-specific estimates of first-phase viral decay rate; CD4+ T-cell restoration (changes from baseline in CD4+ T cell count) as measured at days 7, 14, 28, 56, weeks 24, 48, and final visit; counts of CD4+ and CD8+ T cell subsets that are (a) naïve (CD45RA+/CD27+), (b) CM (CD45RA-/CD27+), and (c) effector memory (CD45RA-/CD27-); and of each of these, counts that are in cell cycle (Ki-67+) and/or activated (express one or both of the activation markers CD38 and HLA-DR).

## 9.3 Randomization

For this single-arm study, randomization is not necessary.

## 9.4 Sample Size and Accrual

A total of 34 subjects will be accrued. Assuming subject enrollment from 7 sites, and a rate of 2 subjects every 3 months at each site, accrual is expected to be completed within 7.5 months.

The primary objective of the study is to estimate the first-phase viral-decay rate in treatment-naïve subjects receiving raltegravir and FTC/TDF. As was done in ACTG viral dynamics substudies A5160s (parent study A5142) and A5166s (A5095), plasma HIV-1 RNA levels will be measured at pre-entry and entry (geometric mean of these to be used as baseline) and as per the Schedule of Events. Plasma HIV-1 RNA measurements through day 56 will be used in estimating the first and second phase viral decay constants d1 and d2. Measurements obtained after treatment interruptions and/or rebound will be excluded. A parametric nonlinear mixed effects model for bi-exponential decay will be fit to the remaining on-treatment data, yielding subject-specific empirical Bayes estimates (EBEs) of d1 and d2. Ninety-five percent CIs around the subject-specific estimates will be reported.

Estimates of d1 obtained for subjects on ACTG A5166s and A5160 are shown in Table 6. For both studies, the difference in d1 between the arms with the best and poorest outcomes (highest and lowest d1; also lowest and highest rates of virologic failure in the parent study) was 0.11. For the three treatment groups in A5166s, population mean estimates (± standard deviations), derived from the estimated covariance matrix on the parameter estimates arising from the MCMC approach, ranged from 0.58±0.06/day to 0.70±0.18/day. An alternate derivation, from the 25th and 75th percentiles around the EBEs reported in Table 2 of the manuscript, and using the rule of thumb that the interquartile range is approximately equal to 1.35 times the standard deviation(41), yields somewhat smaller estimates of 0.10 and 0.12, respectively.[[1]](#footnote-2) When the same back-calculations are applied to the EBEs for d1 reported in the final analysis report for A5160s, SDs of d1 are estimated as 0.09, 0.18 and 0.09 for arms A, B and C, respectively. Móltó et al. reported d1 estimates of 0.80 and 0.62 for their 5- and 4-drug regimens, respectively (a difference of 0.18 between arms); SDs around these estimates were given as 0.13 and 0.18, respectively.(42)

For various combinations of sample size and SD of d1 estimates, Table 7 presents the resulting “plus-minus” amounts (half-widths) of 95% CIs. For example, if the population SD of d1 is 0.20, a sample size of 30 subjects (with usable data) will provide CI lower and upper bounds ± 0.07 around the mean. If the true SD(d1) is smaller than 0.20, CIs will be narrower. Assuming the population mean d1 for the study regimen is 0.80, Table 8 presents the probability that 95% CIs will lie entirely above the value 0.65. For example, if the population mean and SD of d1 are 0.80 and 0.20, respectively, and the sample size is 30 subjects with usable data, there is 98% probability that the 95% CI around the sample mean will exclude (and fall above) the value d1=0.65.

Approximately 14% of subjects who enrolled to A5166s had to be excluded from analysis due to drug interruptions. (HIV-1 RNA levels excluded for non-monotonicity were a subset of these.) To allow for such losses in the current study, a buffer of 4 additional subjects is added to the goal of 30 analysis-eligible subjects, bringing the accrual target to 34.

Table 6. Estimates of d1 and its standard deviation from similarly designed and analyzed viral dynamics studies.

| Study | Arm | Estimated  d1 | Estimated  SD(d1) |
| --- | --- | --- | --- |
| A5166s (A5095) | C: EFV + 3TC/ZDV (superior arm) | *0.67  **0.70 | *0.10  **0.18 |
|  | B: ABC + 3TC/ZDV | *0.56  **0.58 | *0.12  **0.06 |
|  | A: EFV + ABC + 3TC/ZDV | 0.59 | 0.08, -- |
| A5160s (A5142) | C: EFV + 2 NRTIs (superior arm) | 0.64 | 0.09 |
|  | A: EFV + LPV/r | 0.61 | 0.09 |
|  | B: LPV/r + 2 NRTIs | 0.53 | 0.18 |
| Móltó | ENF + EFV + LPV/r + 3TC + TDF | 0.80 | 0.13 |
|  | EFV + LPV/r + 3TC + TDF | 0.62 | 0.18 |

* From subject-specific estimates. ** From MCMC-based population estimates.

Table 7. Expected precision for d1 estimates for various combinations of sample size and underlying population standard deviations around true d1. Precision expressed as ± amounts (half-widths) for 95% CIs (assuming normality of d1).

| Sample size | Population standard deviation around d1 | | | | | |
| --- | --- | --- | --- | --- | --- | --- |
|  | 0.12 | 0.15 | 0.18 | 0.20 | 0.23 | 0.25 |
| 10 | 0.0**9** | 0.**11** | 0.13 | 0.14 | 0.16 | 0.17 |
| 15 | 0.07 | 0.08 | 0.10 | 0.11 | 0.13 | 0.14 |
| 20 | 0.06 | 0.07 | 0.08 | 0.09 | 0.11 | 0.12 |
| 25 | 0.05 | 0.06 | 0.07 | 0.08 | 0.09 | 0.10 |
| 30 | 0.04 | 0.06 | 0.07 | 0.07 | 0.0**9** | 0.09 |

Table 8. Probability that the 95% CI around the estimated d1 will lie entirely above the value 0.65, assuming the population mean d1 and its standard deviation are 0.80 and 0.2, respectively.

| Sample size | Population standard deviation around d1 | | | | | |
| --- | --- | --- | --- | --- | --- | --- |
|  | 0.12 | 0.15 | 0.18 | 0.20 | 0.23 | 0.25 |
| 10 | **94**% | 80% | 65% | 56% | 45% | **40**% |
| 15 | **99**% | **95**% | **85**% | **77**% | 65% | 58% |
| 20 | 100% | 99% | 94% | **89**% | **79**% | 72% |
| 25 | 100% | 100% | 98% | **95**% | 88% | 82% |
| 30 | 100% | 100% | 99% | 98% | **93**% | **89**% |

In a viral dynamic study in 5 subjects receiving PI (RTV) monotherapy, Perelson et al. measured plasma HIV-1 RNA intensively over the first several days.(3) In addition to days 0, 2, and 7, plasma HIV-1 RNA levels were obtained at hours 2, 4, 6, 12, 18, 24, 30, 36, and 42; and days 3, 4, 5 and 6. Estimates of the pharmacologic delay – the time lag between first dose of study drug and the onset of decline in virus load – were made, and were longer than the estimated PK delay (during which drug absorption, distribution and penetration occur). This additional delay was considered a consequence of the mechanism of action of PIs, which (a) render newly produced virus noninfectious, but which inhibit neither (b) the production of virions from already infected cells, nor (c) the infection of new cells by previously produced virus. This last process is the target of integrase inhibitors and, heuristically, the successful inhibition of this upstream step should lead to earlier reduction of plasma HIV-1 RNA. Perelson et al. defined S, the duration of the HIV life cycle, as the time from release of 1 virion from an infected T-cell to the time of release of its first progeny from a newly infected T-cell. Using the time to the onset of plasma HIV-1 RNA decline minus the pharmacologic delay to estimate S, they obtained the very precise estimate of 1.2 ± 0.1 days.(3)

## 9.5 Monitoring

As phase I/II and pilot studies are not routinely reviewed by the Data and Safety Monitoring Board, it is the responsibility of the team safety monitoring committee (TSMC) to interpret toxicity data and make any decisions needed to protect subjects from undue risk. The TSMC will convene once a month to review monitoring reports. At a minimum the TSMC will consist of the protocol chair, the protocol vice chair, the DAIDS medical officer, the biostatisticians, the data manager, and the National Community Advisory Board representative to the team. One or more clinical representatives from participating sites may also participate.

In addition, toxicity summaries will be reviewed annually by the Translational Research and Drug Development (TRADD) Study Monitoring Committee.

The safety and tolerability of the study medication will be monitored by means of adverse event reports (AER) and toxicity summaries presenting counts of subjects by Grade ≥ 3 laboratory and clinical events. It is required that these data be entered into the database within 3 business days of the time at which the results of the laboratory tests or clinical examinations become available. Toxicity summaries will be reviewed monthly via conference calls or by e-mail exchange. Subjects will be managed according to Section 7.0.

The data manager and statistician will also provide accrual reports (number of subjects accrued, number and percent discontinuing for any reason) and reports that identify subjects who meet the criterion for virologic failure. To facilitate sites in providing clarifications, SIDs will not be masked.

## 9.6 Analyses

9.6.1 Primary Objective

Primary objective: To estimate the first-phase viral-decay rate in treatment-naïve subjects receiving raltegravir and FTC/TDF.

The primary analysis will be an AT analysis. Plasma HIV-1 RNA levels collected on days 0 to 56 will be used in parameter estimation except for (1) those collected after discontinuation, interruption, dose modification, or non-adherence to either raltegravir or FTC/TDF during days 0-14 (as determined from treatment records and adherence questionnaires), and (2) those at or after the first sign of rebound (defined as a rise of >0.30 log10 above the previous plasma HIV-1 RNA level). (For a given subject, HIV-1 RNA levels collected prior to such an event will be included in the analysis.)

Population and subject-specific decay parameters d1 and d2 from the bi-exponential model will be estimated by fitting a parametric nonlinear mixed-effects model to log10 plasma HIV-1 RNA levels.(4;5;10;43) Model-based 95% CIs around population estimates for d1 and d2 will be reported, and the distribution of subject-specific estimates will be summarized.

For any plasma specimen for which the HIV-1 RNA level is below the lower limit of detection (LLD) of the Ultrasensitive Roche Amplicor HIV-1 Monitor assay (50 copies/mL), a separate stored sample will be assayed for HIV-1 quantification using the single copy assay. In the event that any such single copy assay results are below the single copy assay LLD (1 copy/mL), to achieve unbiased estimates of d1 and d2 in the presence of censoring imposed by the assay lower LLD, an iterative multiple imputation procedure will be used (5;43); this procedure is described in detail in the analysis plan.

9.6.2 Secondary Objectives

9.6.2.1 To compare first phase viral-decay rates for raltegravir and FTC/TDF to first-phase viral decay rates estimated for the superior arms of A5160s and A5166s.

Primary analysis: The Wilcoxon signed rank test will be used to compare subject-specific d1 estimates (obtained as above) to the value 0.67, which was the point estimate from d1 obtained on the (EFV-based) superior arm of ACTG viral dynamics substudy A5166s.

Secondary analyses:

1. In an AT analysis (i.e., restricting the data as described in 9.6.1), plasma HIV-1 RNA levels from A5248 will be combined with data from A5166s and A5160s.(10;43;44) The resulting subject-specific EBEs of d1 will be compared using the permutation-based Kruskal-Wallis test. (In a 2001 simulation study, compared to parametric tests, Ding and Wu conclude that permutation-based tests outperform likelihood-based tests [maintain type I error rate, and are robust to deviations from normality and small amounts of missing data].(9) In the majority of published viral-dynamics applications, the nonparametric Wilcoxon test is used for inference to compare d1’s among 2 groups.)
2. This nonparametric comparison of subject-specific EBEs will be repeated, with additional plasma HIV-1 RNA levels from A5248 subjects: plasma HIV-1 RNA levels obtained after discontinuation or modification of FTC/TDF, and/or after no more than 7 cumulative days of prescribed raltegravir doses are missed.

9.6.2.2 To estimate the second and third-phase viral decay rates in treatment-naive subjects receiving raltegravir and FTC/TDF, and to estimate the times at which subjects' decay rates transition from first to second phase and from second to third phase.

Subject-specific second-phase decay rates will be estimated along with first-phase decay rates as described in 9.6.1 above. Similar estimation and inference procedures will be used to estimate third-phase decay rates, based on single-copy assay results for plasma HIV-1 RNA samples obtained during weeks 24 to 72.(28;45) The primary analysis will be AT; a secondary analysis may be conducted wherein subjects who discontinued or interrupted the regimen are retained.

Times of transition from first to second phase and from second to third phase will be estimated using the methods of Bosch and colleagues(46) applied to subject-specific predicted log10 HIV-1 RNA from days 0-56 (first to second phase) and applied to log10 HIV-1 RNA.

9.6.2.3 To determine the proportion of subjects with plasma HIV-1 RNA below the limit of detection, <50 copies/mL, 24, 48, and 72 weeks after initiation of raltegravir and FTC/TDF.

The primary analysis will be an ITT analysis, wherein: (1) if the study regimen (raltegravir and/or FTC/TDF) is modified or discontinued the subject is retained in analyses, and (2) if a subject is lost to follow-up, the last observation is carried forward for subsequent time points. The proportion of subjects with plasma HIV-1 RNA <50 copies/mL at weeks 24, 48, and 72 will be reported along with the exact binomial 95% CI.

A secondary AT analysis will include only those subjects on the study regimen at the time of the evaluation.

9.6.2.4 To evaluate the safety and tolerability of the treatment with raltegravir and FTC/TDF.

Among subjects taking one or more dose of study drug, the proportion exhibiting each category of events will be reported, along with exact binomial 95% CI. Incidence rates, i.e., numbers of events per person-months of follow-up, will also be reported.

9.6.2.5 To evaluate changes in CD4+ T-cell counts 24, 48, and 72 weeks after initiation of raltegravir and FTC/TDF.

For the primary analysis, using the ITT approach described in 9.6.2.3, changes in CD4+ T-cell counts at weeks 24, 48, and 72 will be summarized descriptively via mean, SD, median, and quartiles. A secondary AT analysis may also be performed.

9.6.2.6 To investigate the occurrence of raltegravir and FTC/TDF-resistance mutations at virologic failure using very sensitive resistance assay techniques

Among subjects who took any dose of study drug, for each failing subject, the list of resistance mutations present at failure, if any, will be displayed. Of failing subjects, the proportion with specific mutations (ones occurring frequently among A5248 failures, ones known to be associated with raltegravir resistance) will be reported along with exact binomial 95% CIs.

9.6.2.7 To evaluate the relationship of raltegravir and FTC/TDF PK parameters with virologic and immunologic responses, including day 7 plasma HIV-1 RNA change from baseline and the magnitude and slope of changes in CD4+ T-cell counts over the first 14 days of study drug.

Raltegravir, TDF and FTC PK parameters will be estimated via population PK modeling conducted at the University of Alabama Division of Clinical Pharmacology. The following treatment-response markers will be plotted against PK parameters: (1) subject-specific estimates of d1, d2, d3, and d4; (2) day 7 plasma HIV-1 RNA change from baseline; (3) magnitude and slope of CD4+ T-cell count changes over days 0-14; and (4) plasma HIV-1 RNA and CD4+ T-cell count changes from baseline at weeks 24 and 72. Both AT and ITT approaches will be considered.

9.6.2.8 To evaluate adherence by self-report in treatment-naïve subjects receiving raltegravir and FTC/TDF.

For each drug, visit and subject, the numbers and percent of missed doses will be calculated. Results will be summarized descriptively. Relationships between adherence to virologic response and to PK parameters may be investigated.

9.6.2.9 To investigate the decay rate in unintegrated proviral DNA in PBMCs.

The primary analysis will be of subjects as-treated, and will be descriptive. Subject-specific trajectories of absolute quantities vs. time will be plotted (total cell-associated DNA and 3 subsets overlaid; subsets are LTR circular DNA and integrated proviral DNA as measured by an assay currently undergoing validation, and unintegrated linear proviral DNA calculated by subtraction). Medians (across subjects) at each time point will be plotted with corresponding 95% CIs. If subject-specific trajectories show similar shapes, specific features (e.g., slope for each subset, time at number of which LTR circles changes from increasing to decreasing) will be estimated and summarized.

9.6.2.10 To investigate longer-term viral decay in plasma, from single-copy assay results obtained during weeks 24 to 72.

Primary analysis: For weeks 24 to 72, using the ITT approach described in 9.6.2.3, the proportion of subjects with plasma HIV-1 RNA below the limit of detection of the single-copy assay will be reported, along with the exact 95% binomial CI.

9.6.2.11 To examine the relationship between first phase virologic decay, immune activation in defined T-cell maturation subsets and CD4+ T-cell restoration.

The primary AT analysis will exclude endpoints corresponding to samples collected after an interruption or modification in the study regimen. The primary analysis will also include only those subjects for whom d1 could be estimated. Secondary analyses in which d1 is not a variable may include subjects without d1 estimates. Associations between the following pairs of variables will be examined, as described in the analysis plan: (1) baseline plasma HIV-1 RNA levels and baseline proportion of activated CM T-cells, (2) subject-specific d1 estimates and CD4+ T-cell increases (day 14, week 24; separate outcomes), (3) baseline CM T-cell turnover and CD4+ T-cell increases (day 14, week 24; separate outcomes), and (4) subject-specific d1 estimates and baseline CM T-cell turnover.

# 10.0 PHARMACOLOGY PLAN

## 10.1 Pharmacology Objectives

A5248 is a pilot study designed to examine viral decay rates in treatment naïve subjects receiving raltegravir plus FTC/TDF. The primary pharmacology objective is to examine relationships between initial viral decay rates and raltegravir trough concentrations. Secondary objectives include examining relationships between raltegravir trough concentrations and later viral decay rates and the decay rate in unintegrated proviral DNA in PBMCs.

## 10.2 Study Design

Recent experience, additional PK data on raltegravir systemic exposure, and study design limitations limit the types of PK analyses that can be completed in this study. Due to the amount of samples being collected and the current inability to model raltegravir concentration-time data, we will only be collecting trough concentrations on all subjects in the study. Each subject will have a single trough sample collected between 10 and 14 hours post dose at hour 12 (following first-dose), and at each subsequent morning clinic visit. TDF and FTC concentrations will also be measured. A total of 3 mL whole blood should be collected for each trough determination. Dose amounts, dose timing, and samples collection times will be recorded.

## 10.3 Primary and Secondary Data, Modeling, and Data Analysis

The pharmacology design will capture raltegravir trough concentrations from the first dose and out to steady-state. The data for each subject will summarized statistically and used to determine if relationships exist between viral decay and drug exposure. These data may also be used additional subsequent virologic or immunologic analyses completed by the study team.

## 10.4 Anticipated Outcomes

Raltegravir has been shown to be exceptionally potent at lowering the initial viral load. This study design will allow us to collect the data needed to begin establishing relationships between raltegravir exposure and pharmacodynamic markers such as viral decay slope. Raltegravir has considerable PK variability (~7-fold following multiple dosing), especially at the end of the dosing interval. Regardless, at doses of 100 to 600 mg twice daily, most subjects still achieved short-term viral load suppression. The primary explanation for this observation is that even at the lower dose, most subjects maintain trough concentrations at or above the IC95. Raltegravir has minimal drug-drug interactions relative to PIs, but other drugs can still affect its concentration-time profile which will increase the PK variability. The drug is also a substrate for p-glycoprotein (P-gp), which could potentially be another source of increased variability when administered with P-gp inducers or inhibitors.

Given the potency of raltegravir at the doses studied, there is a possibility that no exposure-response relationships will be found (fixed effect). However, given the variability in exposure, one might be established. The goal of this study and subsequent analyses is NOT to develop guidelines for the use of therapeutic drug monitoring for raltegravir, but rather to establish a baseline drug exposure to which ensuing studies (especially drug interaction studies) can be compared to lessen the probability of treatment failure and development of resistance.

# 11.0 DATA COLLECTION AND MONITORING AND ADVERSE EVENT REPORTING

## 11.1 Records to Be Kept

CRFs will be provided for each subject. Subjects must not be identified by name on any CRFs. Subjects will be identified by the PID and SID provided by the ACTG DMC upon registration.

## 11.2 Role of Data Management

11.2.1 Instructions concerning the recording of study data on CRFs will be provided by the ACTG DMC. Each clinical research site is responsible for keying the data in a timely fashion.

11.2.2 It is the responsibility of the ACTG DMC to assure the quality of computerized data for each ACTG study. This role extends from protocol development to generation of the final study databases.

## 11.3 Clinical Site Monitoring and Record Availability

11.3.1 Site monitors under contract to the NIAID will visit participating clinical research sites to review the individual subject records, including consent forms, CRFs, supporting data, laboratory specimen records, and medical records (physicians’ progress notes, nurses’ notes, individuals’ hospital charts), to ensure protection of study subjects, compliance with the protocol, and accuracy and completeness of records. The monitors also will inspect sites’ regulatory files to ensure that regulatory requirements are being followed and sites’ pharmacies to review product storage and management.

- - 1. The site investigator will make study documents (e.g., consent forms, drug distribution forms, CRFs) and pertinent hospital or clinic records readily available for inspection by the local IRB, site monitors, FDA, NIAID, OHRP, and pharmaceutical supporter(s) or designee for confirmation of the study data.

## 11.4 Expedited Adverse Event Reporting to DAIDS

The EAE reporting requirements and definitions for this study and the methods for expedited reporting of AEs to the DAIDS RCC Safety Office are defined in the “Manual for Expedited Reporting of Adverse Events to DAIDS” (DAIDS EAE Manual), dated May 6, 2004. The DAIDS EAE Manual is available on the RCC Web site: [http://rcc.tech-res-intl.com/eae](http://rcc.tech-res-intl.com/).

AEs reported on an expedited basis must be documented on the DAIDS Expedited Adverse Event Reporting form (EAE Reporting Form) available on the RCC Web site: [http://rcc.tech-res-intl.com/eae](http://rcc.tech-res-intl.com/).

EAE Reporting Requirements for this Study

This study uses the standard level of expedited AE reporting as defined in the DAIDS EAE Manual.

The study agents that must be considered in determining relationships of AEs requiring expedited reporting to DAIDS are raltegravir (MK-0518) and emtricitabine/tenofovir disoproxil fumarate (TDF/FTC, Truvada).

The Division of AIDS Table for Grading the Severity of Adult and Pediatric Adverse Events (DAIDS AE Grading Table), Version 1.0, December 2004, must be used and is available on the DAIDS RCC Web site: [http://rcc.tech-res-intl.com/eae/htm](http://rcc.tech-res-intl.com/)/.

Adverse events must be reported on an expedited basis at the standardlevel during the protocol-defined EAE Reporting Period, which is the entire study duration for an individual subject (from study enrollment until study completion or discontinuation of the subject from study participation for any reason).

After the end of the protocol-defined EAE Reporting Period stated above, sites must report serious, unexpected, clinical suspected adverse drug reactions if the study site staff becomes aware of the event on a passive basis, i.e., from publicly available information.

# 12.0 HUMAN SUBJECTS

## 12.1 IRB Review and Informed Consent

This protocol and the informed consent document (Appendix IV) and any subsequent modifications will be reviewed and approved by the IRB or ethics committee responsible for oversight of the study. A signed consent form will be obtained from the subject (or parent, legal guardian, or person with power of attorney for subjects who cannot consent for themselves, such as those below the legal age of consent). The consent form will describe the purpose of the study, the procedures to be followed, and the risks and benefits of participation. A copy of the consent form will be given to the subject, parent, or legal guardian, and this fact will be documented in the subject’s record.

## 12.2 Subject Confidentiality

All laboratory specimens, evaluation forms, reports, and other records that leave the site will be identified by coded number only to maintain subject confidentiality. All records will be kept locked. All computer entry and networking programs will be done with coded numbers only. Clinical information will not be released without written permission of the subject, except as necessary for monitoring by the IRB, ACTG, FDA, NIAID, OHRP, or pharmaceutical supporter(s) or designee.

## 12.3 Study Discontinuation

The study may be discontinued at any time by the ACTG, IRB, NIAID, pharmaceutical supporter(s), FDA, OHRP, or other government agencies as part of their duties to ensure that research subjects are protected.

# 13.0 PUBLICATION OF RESEARCH FINDINGS

Publication of the results of this trial will be governed by ACTG policies. Any presentation, abstract, or manuscript will be made available for review by the pharmaceutical supporter prior to submission.

# 14.0 BIOHAZARD CONTAINMENT

As the transmission of HIV and other blood-borne pathogens can occur through contact with contaminated needles, blood, and blood products, appropriate blood and secretion precautions will be employed by all personnel in the drawing of blood and shipping and handling of all specimens for this study, as currently recommended by the Centers for Disease Control and Prevention and the National Institutes of Health.

All dangerous goods materials, including diagnostic specimens and infectious substances, must be transported according to the instructions detailed in the International Air Transport Association (IATA) Dangerous Goods Regulations.

15.0 REFERENCES

(1) Gallant JE, Rodriguez AE, Weinberg WG et al. Early virologic nonresponse to tenofovir, abacavir, and lamivudine in HIV-infected antiretroviral-naive subjects. J Infect Dis 2005; 192(11):1921-1930.

(2) Gulick RM, Ribaudo HJ, Shikuma CM et al. Triple-nucleoside regimens versus efavirenz-containing regimens for the initial treatment of HIV-1 infection. N Engl J Med 2004; 350(18):1850-1861.

(3) Perelson AS, Neumann AU, Markowitz M, Leonard JM, Ho DD. HIV-1 dynamics in vivo: virion clearance rate, infected cell life-span, and viral generation time. SCIENCE 1996; 271(5255):1582-1586.

(4) Wu H, Ding AA, De G, V. Estimation of HIV dynamic parameters. Stat Med 1998; 17(21):2463-2485.

(5) Wu H, Ding AA. Population HIV-1 dynamics in vivo: applicable models and inferential tools for virological data from AIDS clinical trials. Biometrics 1999; 55(2):410-418.

(6) Wu H, Zhang JT. The study of long-term HIV dynamics using semi-parametric non-linear mixed-effects models. Stat Med 2002; 21(23):3655-3675.

(7) Ding AA, Wu H. Relationships between antiviral treatment effects and biphasic viral decay rates in modeling HIV dynamics. Math Biosci 1999; 160(1):63-82.

(8) Notermans DW, Goudsmit J, Danner SA, de WF, Perelson AS, Mittler J. Rate of HIV-1 decline following antiretroviral therapy is related to viral load at baseline and drug regimen. AIDS 1998; 12(12):1483-1490.

(9) Ding AA, Wu H. Assessing antiviral potency of anti-HIV therapies in vivo by comparing viral decay rates in viral dynamic models. Biostatistics 2001; 2(1):13-29.

(10) Kuritzkes DR, Ribaudo HJ, Squires KE et al. Plasma HIV-1 RNA dynamics in antiretroviral-naive subjects receiving either triple-nucleoside or efavirenz-containing regimens: ACTG A5166s. J Infect Dis 2007; 195(8):1169-1176.

(11) Riddler SA, Haubrich R, DiRienzo G et al. A prospective, randomized, phase III trial of NRTI-, PI-, and NNRTI-sparing regimens for initial treatment of HIV-1 infection- ACTG 5142. XVI International AIDS Conference, Toronto Canada, Abstract THLB0204, August 13-16, 2006.

(12) Haubrich R, Ribaudo HJ, DiRienzo G et al. Initial viral decay to assess the relative antiretroviral potency of PI-, NNRTI-, and NRTI-sparing regimens for first line therapy of HIV-1 infection: ACTG 5160s. 14th Conference on Retroviruses and Opportunistic Infections, Los Angeles, CA, Abstract #137, August 25-28, 2007.

(13) Cooper D, Gatell J, Rockstroh J et al. Results of BENCHMRK-1, a phase III study evaluating the efficacy and safety of MK-0518, a novel HIV-1 integrase inhibitor, in patients with triple-class resistant virus. 14th Conference on Retroviruses and Opportunistic Infections, Los Angeles, CA, Abstract #105aLB, February 25-28, 2007.

(14) Steigbigel R, Kumar P, Eron J et al. Results of BENCHMRK-2, a phase III study evaluating the efficacy and safety of MK-0518, a novel HIV-1 integrase inhibitor, in patients with triple-class resistant virus. 14th Conference on Retroviruses and Opportunistic Infections, Los Angeles, CA, Abstract #105bLB, February 25-28, 2007.

(15) Markowitz M, Morales-Ramirez JO, Nguyen BY et al. Antiretroviral activity, pharmacokinetics, and tolerability of MK-0518, a novel inhibitor of HIV-1 integrase, dosed as monotherapy for 10 days in treatment-naive HIV-1-infected individuals. J Acquir Immune Defic Syndr 2006; 43(5):509-515.

(16) Han Y, Wind-Rotolo M, Yang HC, Siliciano JD, Siliciano RF. Experimental approaches to the study of HIV-1 latency. Nat Rev Microbiol 2007; 5(2):95-106.

(17) Butler SL, Hansen MS, Bushman FD. A quantitative assay for HIV DNA integration in vivo. Nat Med 2001; 7(5):631-634.

(18) Hindmarsh P, Leis J. Retroviral DNA integration. Microbiol Mol Biol Rev 1999; 63(4):836-43, table.

(19) Lataillade M, Kozal MJ. The hunt for HIV-1 integrase inhibitors. AIDS Patient Care STDS 2006; 20(7):489-501.

(20) Schroder AR, Shinn P, Chen H, Berry C, Ecker JR, Bushman F. HIV-1 integration in the human genome favors active genes and local hotspots. Cell 2002; 110(4):521-529.

(21) Brussel A, Sonigo P. Analysis of early human immunodeficiency virus type 1 DNA synthesis by use of a new sensitive assay for quantifying integrated provirus. J Virol 2003; 77(18):10119-10124.

(22) Wu H, Kuritzkes DR, McClernon DR et al. Characterization of viral dynamics in human immunodeficiency virus type 1-infected patients treated with combination antiretroviral therapy: relationships to host factors, cellular restoration, and virologic end points. J Infect Dis 1999; 179(4):799-807.

(23) Picker LJ, Hagen SI, Lum R et al. Insufficient production and tissue delivery of CD4+ memory T cells in rapidly progressive simian immunodeficiency virus infection. J Exp Med 2004; 200(10):1299-1314.

(24) Sieg SF, Rodriguez B, Asaad R, Jiang W, Bazdar DA, Lederman MM. Peripheral S-phase T cells in HIV disease have a central memory phenotype and rarely have evidence of recent T cell receptor engagement. J Infect Dis 2005; 192(1):62-70.

(25) Brenchley JM, Price DA, Schacker TW et al. Microbial translocation is a cause of systemic immune activation in chronic HIV infection. Nat Med 2006; 12(12):1365-1371.

(26) Meier A, Alter G, Frahm N et al. MyD88-dependent immune activation mediated by human immunodeficiency virus type 1-encoded toll-like receptor ligands. J Virol 2007; 81(15):8180-8191.

(27) Biancotto A, Grivel JC, Iglehart SJ et al. Abnormal activation and cytokine spectra in lymph nodes of people chronically infected with HIV-1. Blood 2007; 109(10):4272-4279.

(28) Maldarelli F, Palmer S, King MS et al. ART suppresses plasma HIV-1 RNA to a stable set point predicted by pretherapy viremia. PLoS Pathog 2007; 3(4):e46.

(29) King MS, Palmer S, Wiegand A et al. Low-Level Viremia Decays Over 7+ Years to a Residual Level Correlated with Baseline HIV-1 RNA. CROI 2007.

(30) Markowitz M, Nguyen BY, Gotuzzo E et al. Rapid and Durable Antiretroviral Effect of the HIV-1 Integrase Inhibitor Raltegravir as Part of Combination Therapy in Treatment-Naive Patients With HIV-1 Infection: Results of a 48-Week Controlled Study. J Acquir Immune Defic Syndr 2007.

(31) Murray JM, Emery S, Kelleher A et al. The integrase inhibitor raltegravir alters viral decay kinetics of HIV, significantly reducing the second phase and challenging current hypotheses of viral replication. 4th International AIDS Society Conference, Sydney, Australia, Abstract TUAB103, July 22-25, 2007.

(32) Hazuda DJ, Miller MD, Zhao J. Resistance to the HIV integrase inhibitor raltegravir: Analysis of Protocol 005, a phase 2 study in patients with triple-class resistant HIV-1 infection. 16th International HIV Drug Resistance Workshop, Barbados, West Indies, Abstract #8, June 12-16, 2007.

(33) Johnson MA, Gathe JC, Jr., Podzamczer D et al. A once-daily lopinavir/ritonavir-based regimen provides noninferior antiviral activity compared with a twice-daily regimen. J Acquir Immune Defic Syndr 2006; 43(2):153-160.

(34) Gallant JE, DeJesus E, Arribas JR et al. Tenofovir DF, emtricitabine, and efavirenz vs. zidovudine, lamivudine, and efavirenz for HIV. N Engl J Med 2006; 354(3):251-260.

(35) Pozniak AL, Gallant JE, DeJesus E et al. Tenofovir disoproxil fumarate, emtricitabine, and efavirenz versus fixed-dose zidovudine/lamivudine and efavirenz in antiretroviral-naive patients: virologic, immunologic, and morphologic changes--a 96-week analysis. J Acquir Immune Defic Syndr 2006; 43(5):535-540.

(36) Shiffman M, Ng TM, Krastev Z, et al. A double-blind, placebo-controlled trial of emtricitabine (FTC, Emtriva) administered once-daily for treatment of chronic hepatitis B virus (HBV) infection (oral #22). 55th Annual Meeting of the American Association for the Study of Liver Diseases, Boston, MA, October 29-November 2, 2004.

(37) Nunez M, Perez-Olmeda M, Diaz B, Rios P, Gonzalez-Lahoz J, Soriano V. Activity of tenofovir on hepatitis B virus replication in HIV-co-infected patients failing or partially responding to lamivudine. AIDS 2002; 16(17):2352-2354.

(38) Reynes J, Peyriere H, Merle de Boever C, Le Moing V. Renal tubular injury and severe hypophosphotemia (Fanconi syndrome) associated with tenofovir therapy (abstract 717). 10th Conference on Retroviruses and Opportunistic Infections, Boston, MA, February 10-14, 2003.

(39) Coca S, Perazella MA. Rapid communication: acute renal failure associated with tenofovir: evidence of drug-induced nephrotoxicity. Am J Med Sci 2002; 324(6):342-344.

(40) Gallant JE, Staszewski S, Pozniak AL et al. Efficacy and safety of tenofovir DF vs stavudine in combination therapy in antiretroviral-naive patients: a 3-year randomized trial. JAMA 2004; 292(2):191-201.

(41) Metzler CM. Statistical Criteria. In: Welling PG, Tse FLS, Dighe SV, editors. Pharmaceutical Bioequivalence. New York: Dekker, 1991: 35-67.

(42) Molto J, Ruiz L, Valle M et al. Increased antiretroviral potency by the addition of enfuvirtide to a four-drug regimen in antiretroviral-naive, HIV-infected patients. Antivir Ther 2006; 11(1):47-51.

(43) Ribaudo HJ. A5166s: A Viral Dynamics Substudy of A5095. Technical Report (ACTG Final Analysis Report). 12-21-2004.
Ref Type: Report

(44) Ribaudo R, Bettendorf D. A5160s: A viral Dynamics Substudy of A5142. Technical Report (ACTG Final Analysis Report). 5-17-2006.
Ref Type: Report

(45) King MS, Palmer S, Wiegand A et al. Low-Level Viremia Decays Over 7+ Years to a Residual Level Correlated with Baseline HIV-1 RNA. 14th Conference on Retroviruses and Opportunistic Infections, Los Angeles, CA, Poster 290 2007.

(46) Bosch RJ, Wang R, Vaida F, Lederman MM, Albrecht MA. Changes in the slope of the CD4 cell count increase after initiation of potent antiretroviral treatment. J Acquir Immune Defic Syndr 2006; 43(4):433-435.

# APPENDIX I: Substudy A5249s: INTENSIVE VIRAL DYNAMICS SUBSTUDY OF A5248

The ACTG Translational Research and Drug

Development (TRADD) Committee: Cara Wilson, M.D., Chair

Substudy Protocol Chair: Adriana Andrade, M.D., M.P.H.

Substudy Protocol Vice Chair: Daniel Kuritzkes, M.D.

Substudy DAIDS Medical Officer: Sarah Read, M.D.

Substudy Clinical Trials Specialist: Joelle Touw, B.S.

A5249s SUBSTUDY TEAM ROSTER

Chair

Adriana Andrade, M.D., M.P.H.

Division of Infectious Diseases

Johns Hopkins University

1830 East Monument Street, Suite 8074

Baltimore, MD 21205

Phone: (410) 614-4036

FAX: (410) 614-9978

E-Mail: [aandrade@jhmi.edu](../aandrade@jhmi.edu)

Vice Chair

Daniel Kuritzkes, M.D.

Brigham and Women’s Hospital

Harvard Medical School

65 Landsdowne Street, Room 447

Cambridge, MA 02139

Phone: (617) 768-8371

FAX: (617) 768-8738

E-Mail: [dkuritzkes@partners.org](mailto:dkuritzkes@partners.org)

DAIDS Medical Officer

Sarah Read, M.D.

HIV Research Branch

TRP, DAIDS, NIAID, NIH

Room 5218

6700-B Rockledge Drive

Bethesda, MD 20892-7624

Phone: (301) 451-2757

FAX: (301) 435-9282

E-Mail: readsa@niaid.nih.gov

Clinical Trials Specialists

Joelle Touw, B.S.

ACTG Operations Center

8757 Georgia Avenue, 12th Floor

Silver Spring, MD 20910-3714

Phone: (301) 628-3000

FAX: (301) 628-3302

E-Mail: [jtouw@s-3.com](../jtouw@s-3.com)

Clinical Trials Specialists (cont.)

Beatrice Kallungal, B.S.

ACTG Operations Center

8757 Georgia Avenue, 12th Floor

Silver Spring, MD 20910-3714

Phone: (301) 628-3377

FAX: (301) 628-3302

E-mail: [bkallungal@s-3.com](mailto:bkallungal@s-3.com)

Statisticians

Susan Rosenkranz, Ph.D.

Statistical and Data Analysis Center

Harvard School of Public Health

900 Commonwealth Avenue, 2nd Floor

Boston, MA 02215

Phone: (617) 632-5915

FAX: (617) 632-2001

E-Mail: [sue@sdac.harvard.edu](mailto:sue@sdac.harvard.edu)

Song Yu, M.S.

Statistical and Data Analysis Center

Harvard School of Public Health

900 Commonwealth Avenue, 2nd Floor

Boston, MA 02215

Phone: (617) 632-2010

FAX: (617) 632-2001

E-Mail: [syu@sdac.harvard.edu](../syu@sdac.harvard.edu)

Data Manager

Barbara Schock, M.S.

Frontier Science & Technology Research Foundation, Inc.

4033 Maple Road

Amherst, NY 14226-1056

Phone: (716) 834-0900x7270

FAX: (716) 834-8432

E-Mail: [schock.barbara@fstrf.org](mailto:schock.barbara@fstrf.org)

Immunologist

Michael Lederman, M.D.

Case Western Reserve University

University Hospitals of Cleveland

The Foley Building, Room 401-A

2061 Cornell Road

Cleveland, OH 44106-5083

Phone: (216) 844-8786

FAX: (216) 844-5523

E-Mail: [MXL6@case.edu](../../../../Local%20Settings/Temporary%20Internet%20Files/Content.IE5/YM9Z6DMK/MXL6@case.edu)

Virologist

John Mellors, M.D.

Division of Infectious Diseases

University of Pittsburgh Medical Center

Scaife Hall, Suite 818

3550 Terrace Street

Pittsburgh, PA 15261

Phone: (412) 624-8512

FAX: (412) 383-7982

E-Mail: [mellors@dom.pitt.edu](mailto:mellors@dom.pitt.edu)

Pharmacologist

Edward Acosta, Pharm.D.

Department of Pharmacology and Toxicology

Division of Clinical Pharmacology

University of Alabama at Birmingham

1530 3rd Avenue South, VH 116

Birmingham, AL 35294-0019

Phone: (205) 934-2655

FAX: (205) 934-6201

E-Mail: [eacosta@uab.edu](mailto:eacosta@uab.edu)

Investigators

Eric Daar, M.D.

Harbor-UCLA Medical Center

Los Angeles Biomedical Research Institute

1124 W. Carson St., Building N-24

Torrance, CA 90502

Phone: (310) 222-2467

FAX: (310) 533-0447

E-Mail: [edaar@labiomed.org](../edaar@labiomed.org)

Investigators (cont.)

Judith Feinberg, M.D.

University of Cincinnati Medical Center

Holmes Hospital

Eden Avenue and Sabin Way

Room 3112

Cincinnati, OH 45267-0405

Phone: (513) 584-5897

Fax: (513) 584-6040

E-Mail: [judith.feinberg@uc.edu](mailto:judith.feinberg@uc.edu)

Charles Flexner, M.D.

Johns Hopkins University Hospital

Osler 524
600 North Wolfe Street
Baltimore, MD 21287-5554
Phone: (410) 955-9712
FAX: (410) 955-9708

E-Mail: [flex@jhmi.edu](mailto:flex@jhmi.edu)

Joel Gallant, M.D., M.P.H.

Johns Hopkins AIDS Service

Division of Infectious Diseases

Johns Hopkins University School of Medicine

1830 E. Monument Street, #443

Baltimore, MD 21287

Phone: (410) 955-7473

FAX: (410) 614-8099

E-Mail: [jgallant@jhmi.edu](../jgallant@jhmi.edu)

Jeffrey Jacobson, M.D.

Division of Infectious Diseases and HIV Medicine

Drexel University College of Medicine

245 N. 15th Street, MS461

Philadelphia, PA 19102

Phone: (215) 762-6555

E-Mail: [jeffrey.jacobson@drexelmed.edu](../jeffrey.jacobson@drexelmed.edu)

Field Representative

Karen Cavanagh, R.N.

New York Univ. Medical Center

ACTU, C&D Building, Old Bellevue

550 1st Avenue

New York, NY 10016

Phone: (212) 263-6565

FAX: (212) 263-8264

E-Mail: [karen.cavanagh@med.nyu.edu](mailto:karen.cavanagh@med.nyu.edu)

NCAB Representative

Patrick Kramme

1907 W. Alder Grove Drive

Tucson, AZ 85704

Phone: (520) 668-5298

E-Mail: [patkramme@aol.com](mailto:patkramme@aol.com)

Industry Representative

Randi Leavitt, M.D.

Infectious Diseases

Merck and Company

10 Sentry Pkwy. Mail Stop BL 3-4

Blue Bell, PA 19422

Phone: (484) 344-2672

FAX: (484) 344-7325

E-Mail: [randi_leavitt@merck.com](mailto:randi_leavitt@merck.com)

Laboratory Data Coordinators

Travis Behm, B.S.

Frontier Science and Technology Research Foundation

4033 Maple Road

Amherst, NY 14228

Phone: (716) 834-0900x7377

FAX: (716) 833-0655

E-Mail: [tbehm@fstrf.org](mailto:tbehm@fstrf.org)

Ken Braun, B.A.

Frontier Science & Technology Research Foundation

4033 Maple Road

Amherst, NY 14226

Phone: (716) 834-0900x7220

FAX: (716) 833-8432

E-Mail: [braun@fstrf.org](mailto:braun@fstrf.org)

SITES PARTICIPATING IN THE SUBSTUDY

A5249s is a limited-site substudy. Sites participating in A5249s are posted to the protocol-specific Web page at <http://aactg.s-3.com/members/ps/5248/ps5248.htm>.

SCHEMA

A5249s: INTENSIVE VIRAL DYNAMICS SUBSTUDY OF A5248

DESIGN This optional viral dynamics substudy of A5248 will use intensive HIV-1 RNA sampling to estimate the time at which first phase viral decay begins following initiation of raltegravir and FTC/TDF in ARV-naïve subjects. Subjects will undergo intensive HIV-1 RNA measurements over the first several days of early first decay phase to fully characterize the effects of raltegravir and FTC/TDF on the kinetics of HIV.

DURATION 7 days (2 nights will be spent in the hospital/ general clinical research center [GCRC])

SAMPLE SIZE 10 subjects

POPULATION 10 ARV-naïve subjects from A5248

1. HYPOTHESIS AND STUDY OBJECTIVE
   1. Hypothesis

In treatment-naïve subjects starting a potent ARV regimen consisting of raltegravir and FTC/TDF, plasma HIV-1 RNA levels will show measurable decline within the first 12-18 hours after treatment initiation, and possibly earlier.

1.2 Objective

To estimate the pharmacologic delay, i.e., the time at which first phase viral decay begins, immediately following initiation of raltegravir and FTC/TDF in ARV-naïve subjects.

1. INTRODUCTION

2.1 Background and Rationale

The use of viral dynamic models has shown HIV-1 viral decay rates to be correlated with early virologic response to potent therapy.(1) Recent data suggest that early virologic response may be predictive of long-term virologic outcome.(2-4) This observation has lead to the generation of viral dynamics models and has provided a theoretical rationale for using first-phase decay rates to compare the short term activity of various regimens.(5-10)

Some of the most interesting data from viral dynamic studies come from examination of the very early decay phase, as defined by Perelson and colleagues.(8) In a viral dynamic study in 5 subjects receiving RTV monotherapy, Perelson et al. measured viral load intensively over the first several days. In addition to days 0, 2, and 7, plasma HIV-1 RNA was measured at hours 2, 4, 6, 12, 18, 24, 30, 36, and 42; and days 3, 4, 5, and 6. Estimates of the pharmacologic delay, the time lag between first dose of study drug and the onset of decline in virus load, were made, and were longer than the estimated PK delay (i.e., the delay due to drug absorption, distribution, and cell/compartment penetration). This additional delay was felt to be a consequence of the mechanism of action of PIs, which render newly produced virus noninfectious, but which inhibit neither the production of virions from already infected cells nor the infection of new cells by previously produced virus. This last process is the target of the ARV class of integrase inhibitors and, heuristically, the successful inhibition of this upstream step should lead to earlier reduction of plasma HIV-1 RNA. In a small study of subjects initiating RTV monotherapy, Perelson and colleagues estimated this pharmacologic delay to be 1.2 ± 0.1 days.

Despite the demonstrated potency of raltegravir in treatment-naïve and ARV-experienced subject trials, no formal viral dynamics studies have been carried out as of yet. The much faster viral decay observed with raltegravir-based regimens compared to other ARV combinations, could be explained by more effective suppression of virus replication (closer to ideal activity), shorter pharmacological lag, or a combination of these two factors. The best way to distinguish between these possibilities is to examine viral decay rates in the first 48 hours of raltegravir treatment.

We will conduct a substudy of A5248 to estimate the time to start of viral-load decline, immediately following initiation of raltegravir and FTC/TDF in ARV-naïve subjects. Although viral decay data will be obtained from A5248 main study, this substudy will generate intensive virologic sampling data in the first few days following treatment initiation, and will provide an estimate of the time of onset of first-phase decay in treatment-naive subjects treated with raltegravir and FTC/TDF.

3.0 STUDY DESIGN

A5249s, the optional viral dynamics substudy of A5248, will be conducted in 10 men and women registered to A5248. It will use intensive HIV-1 RNA sampling to estimate the time of onset of first phase viral decline and its relationship with long-term virologic response, immediately following initiation of raltegravir and FTC/TDF in ARV-naïve subjects. Subjects will undergo intensive HIV-1 RNA sampling during the first decay phase to fully characterize the effects of raltegravir and FTC/TDF on the very early kinetics of HIV.

For accurate estimation of viral decay rates, it is important that subjects are taking their medications as prescribed during the intensive sampling phase (i.e., from entry to day 7). Subjects who permanently discontinue treatment during this period will be discontinued from the study and will be replaced. Sites must notify the protocol team if subjects miss doses or prematurely discontinue A5248 study medication while on A5249s. Subjects will also be questioned about drug holds and adherence. Only data collected prior to the drug discontinuation, drug hold or non-adherence will be used in the primary analysis.

4.0 SELECTION AND ENROLLMENT OF SUBJECTS

4.1 Inclusion Criteria

4.1.1 Enrollment in A5248

4.1.2 Signed A5249s informed consent

4.2 Enrollment Procedures

This substudy will be limited to sites participating in A5248. Prior to implementation, sites must have this substudy and consent form approved by their local IRB. Sites must be registered with and approved by the DAIDS RCC Protocol Registration Office. Substudy registration must occur before any subjects can be enrolled in this substudy.

Once a candidate for entry has been identified, details will be carefully discussed with the subject. The subject will be asked to read and sign the approved substudy informed consent.

NOTE: Subjects must enroll into this substudy at the time of enrollment into the A5248 main study. The team encourages sites to offer compensation to subjects for participation in the substudy.

4.3 ACTG DMC Registration

All eligible subjects will be registered to A5249s by the ACTG DMC (FSTRF) according to standard procedures.

5.0 STUDY TREATMENT

5.1 Regimens, Administration, and Duration

There will be no medications provided by this substudy. Subjects will continue to take their A5248 study ARV regimen. See section 5.0 of A5248 for study treatment requirements and concomitant medications.

6.0 EVALUATIONS

6.1 Schedule of Events

| Evaluation | Screen-ing | Pre-entry | Entry Evaluations | | | | | | Post-Entry Evaluations | | | | | | | | | |
| --- | --- | --- | --- | --- | --- | --- | --- | --- | --- | --- | --- | --- | --- | --- | --- | --- | --- | --- |
| Day |  |  | 0 | | | | | | 1 | | | | | 2 | | 3 | 4 | 7 |
| Week |  |  | 1 | | | | | | 1 | | | | | 1 | | 1 | 1 | 1 |
|  |  |  | Sample time, relative to first dose of study drug and allowed window | | | | | | | | | | | | | | | |
|  |  |  | 0h (pre-dose) | 2h (±30 min) | 4h | 6h | | 12h | | 18h | 24h | 30h | 36h | 42h | Day2 | Day3 | Day4 | Day7 |
| HIV-1 RNA Plasma Sample | X | X | X | X | X | X | | X | | X | X | X | X | X | X | X | X | X |
| Dose Administered in Clinic/GCRC |  |  | X |  |  |  | | X | |  | X |  |  |  | X | X | X | X |
| Medication Diary1 |  |  |  |  |  |  | |  | |  |  |  |  |  | D | C,D | C,D | C |
| Example, first dose of study drug given Monday at 9:00 | | | Monday | | | | | | | Tuesday | | | | Wednesday | | Thursday | Friday | Monday |
|  |  |  | 8:55 | 11:00 | 13:00 | | 15:00 | 21:00 | | 3:00 | 9:00 | 15:00 | 21:00 | 3:00 | 9:00 | 9:00 | 9:00 | 9:00 |

1D=distributed, C=collected.

6.2 Timing of the Evaluations

6.2.1 Screening

6.2.1.1 Subjects must sign the A5249s informed consent.

6.2.1.2 HIV-1 RNA results from the sample drawn for the A5248 screening visit will be used for the substudy.

6.2.2 Pre-entry, Entry, and Post-Entry

- - - 1. HIV-1 RNA results from samples drawn for the A5248 study visits at pre-entry, entry (day 0), day 2, and day 7 will be used for this substudy.

NOTE: A separate tracking form will be used for HIV-1 RNA samples

taken from substudy subjects at entry (day 0), day 2, and day

7.

- - - 1. After starting study medications, samples will be obtained as part of this substudy as per the Schedule of Events. Subjects will be required to stay overnight in the hospital/GCRC from Day 0 to Day 2. On days 2 through 7, the morning study medication dose will be given in the clinic.
      2. Medication diaries will be given to subjects at the entry visit with instructions to record the times they take their study medications for the first 7 days of the study. Subjects will bring their medication diaries to their study visits on days 2 through 7.

6.2.3 Discontinuation Evaluations

Premature Discontinuation of A5248 Study Drugs

Subjects who prematurely discontinue from the A5248 Study Drugs will be discontinued from the A5249s substudy.

Premature Discontinuation of Substudy A5249s

Subjects who discontinue participation in this substudy may continue participation in the main A5248 study.

Premature Discontinuation of the A5248 Study

Subjects who prematurely discontinue from the A5248 study will be discontinued from the A5249s substudy.

6.3 Definitions and Special Instructions

6.3.1 Consent Form

A signed and dated, IRB- and DAIDS RCC Protocol Registration Office-approved consent form is required prior to participation in this study.

6.3.2 HIV-1 RNA Samples

Sample Collection, Processing, and Shipping.

Samples will be collected at the time points specified in section 6.1. Refer to Appendix III for processing and shipping instructions.

Storage of additional aliquots of plasma is allowed and strongly encouraged.

6.3.3 Medication Diary

Subjects will be asked to keep a record of the times they took their study medications in a diary supplied to them by the site study personnel at the entry visit. They will be asked to keep the medication diary for the first week of the study.

Subjects will be asked to bring the diary to the clinic on days 3, 4, and 7 to be reviewed with the study staff prior to blood collection. If any study medications have been missed since the last visit, then no further blood samples for A5249s should be collected, and the subjects will be discontinued from the substudy. On day 7, study personnel will retain the diary. Whether or not a subject missed doses and times of missed doses should be keyed in the Antiretroviral Regimen record for A5248 and diaries should be retained in the source documentation.

7.0 TOXICITY MANAGEMENT

See section 7.0 in protocol A5248.

8.0 CRITERIA FOR SUBSTUDY DISCONTINUATION

- Subjects who miss doses of their A5248 study medications between entry and day 7 may continue in the substudy (but will be replaced in accrual). Please notify the protocol team.
- Subjects who discontinue participation in A5249s may still be followed in A5248.
- Subjects who discontinue participation in A5248 must discontinue participation in this A5249s Substudy.

1. STATISTICAL CONSIDERATIONS

In a subset of A5248 subjects, intensive measurement of plasma HIV-1 RNA over the first few days following treatment initiation will be conducted. In addition to sample times of the parent study, plasma samples for quantification of HIV-1 RNA will be collected as per the Schedule of Events. Intensive sampling will allow precise estimation of the time at plasma HIV-1 RNA begins to decline following initiation of the integrase-inhibitor-based regimen. The hypothesis is that this “shoulder” will be of shorter duration than that of regimens without an integrase inhibitor. The primary analysis will be AT, retaining only those subjects who continue to take all drugs in the regimen as prescribed through day 7. Subjects who discontinue or modify the raltegravir and FTC/TDF regimen prior to day 7 will be replaced in accrual to the substudy. Data from these subjects may be retained for secondary ITT analyses.

9.1 Sample Size

The substudy will accrue 10 subjects from A5248. Subjects who discontinue any of the drugs in the regimen before day 8 will be replaced in accrual to the substudy (but will continue to be followed in the substudy).

In a study of 5 HIV-1 infected subjects initiating RTV monotherapy, Perelson et al. performed intensive sampling of plasma HIV-1 RNA over the first few days of treatment.(8) In each subject, they observed an initial lag followed by approximately exponential decline in plasma HIV-1 RNA. A PK brief delay was expected, corresponding to absorption and distribution of drug. Actual delays were longer than expected: 1.2 ± 0.1 days (mean ± SD; range 1.0 to 1.3).

The coefficient of variation (CV = SD/mean) in pharmacologic delay reported by Perelson et al. was 8%. Table 1 gives expected precisions of 95% CIs around mean delay for various sample sizes and CVs (precisions are expressed as a percent of the mean). If the proposed study yields a CV for pharmacologic delay of 24%, three times that seen by Perelson, a sample size of 10 is still expected to provide a fairly precise 95% CI, ranging from -17% to +17% of the mean.(8) For example, if the mean length of the pharmacologic delay from the proposed study is 0.5 days (12 hours), the 95% CI is expected to have a half-width of 0.09 days (2 hours).

Table 1. For several sample sizes and CVs, expected precision (in half-widths, as

percent of mean) of a 95% confidence interval around pharmacologic delay

(in days).

| Sample  size | CV | | |
| --- | --- | --- | --- |
|  | 8% | 16% | 24% |
| 5 | 10% | 20% | 30% |
| 10 | 6% | 11% | 17% |
| 15 | 4% | 9% | 13% |

9.2 Analysis

Primary Objective: To estimate the pharmacologic delay, i.e., the time at which first phase viral decay begins, immediately following initiation of raltegravir and FTC/TDF in ARV-naïve subjects.

In Perelson, delays were estimated by visual inspection of graphs of HIV-1 RNA vs. time, as the length of the shoulder (time before exponential decay began, during which plasma HIV-1 RNA was relatively stable).(8) Results from this approach will be compared to those obtained by more a formal statistical method used to characterize the leveling off of CD4+ T-cell counts in subjects initiating a new ARV regimen (ACTG 364).(1;2) In the latter method, a linear mixed-effects model with three fixed-effects parameters is fit to plasma HIV-1 RNA. The three parameters correspond to an intercept (here, HIV-1 RNA at hour zero), an early slope (here, expected to be zero) and a later slope. A specific transition time (at which slope changes) is posited. Using the method of profile likelihoods: (1) the model is fit repeatedly with a different posited transition time each time, (2) for each fit, the log-likelihood of the data given the model is retained, and (3) the log-likelihoods are plotted against the posited transition times. The transition time at which the log-likelihood is greatest corresponds to the maximum likelihood estimate for transition time. Ghosh and Vaida have used nonlinear mixed-effects modeling to allow transition time to vary across subjects; however this approach may be difficult to implement with a small number of subjects.(2)

10.0 DATA COLLECTION AND MONITORING AND ADVERSE EXPERIENCE REPORTING

Data collection, monitoring, and AE reporting will be according to A5248.

11.0 HUMAN SUBJECTS

IRB review, subject confidentiality, and study discontinuation procedures will be the same as A5248. Subjects will need to sign a separate informed consent for the substudy.

12.0 PUBLICATION OF RESEARCH FINDINGS

Publication of the results of this trial will be the same as for A5248. The intention is to publish the substudy results together with results of the main study.

13.0 BIOHAZARD CONTAINMENT

Precautions and procedures will be as in A5248.

14.0 REFERENCES

(1) Bosch RJ, Wang R, Vaida F, Lederman MM, Albrecht MA. Changes in the slope of the CD4 cell count increase after initiation of potent ARV treatment. J Acquir Immune Defic Syndr 2006; 43(4):433-435.

(2) Ghosh P, Vaida F. Random changepoint modelling of HIV immunologic responses. Stat Med 2007; 26(9):2074-2087.

(3) Hall CB, Lipton RB, Sliwinski M, Stewart WF. A change point model for estimating the onset of cognitive decline in preclinical Alzheimer's disease. Stat Med 2000; 19(11-12):1555-1566.

(4) Notermans DW, Goudsmit J, Danner SA, de WF, Perelson AS, Mittler J. Rate of HIV-1 decline following ARV therapy is related to viral load at baseline and drug regimen. AIDS 1998; 12(12):1483-1490.

(5) Kuritzkes DR, Ribaudo HJ, Squires KE et al. Plasma HIV-1 RNA dynamics in ARV-naive subjects receiving either triple-nucleoside or efavirenz-containing regimens: ACTG A5166s. J Infect Dis 2007; 195(8):1169-1176.

(6) Riddler SA, Haubrich R, DiRienzo G et al. A prospective, randomized, phase III trial of NRTI-, PI-, and NNRTI-sparing regimens for initial treatment of HIV-1 infection- ACTG 5142. XVI International AIDS Conference, Toronto Canada, Abstract THLB0204, August 13-16, 2006.

(7) Haubrich R, Ribaudo HJ, DiRienzo G et al. Initial viral decay to assess the relative ARV potency of PI-, NNRTI-, and NRTI-sparing regimens for first line therapy of HIV-1 infection: ACTG 5160s. 14th Conference on Retroviruses and Opportunistic Infections, Los Angeles, CA, Abstract #137, August 25-28, 2007.

(8) Perelson AS, Neumann AU, Markowitz M, Leonard JM, Ho DD. HIV-1 dynamics in vivo: virion clearance rate, infected cell life-span, and viral generation time. SCIENCE 1996; 271(5255):1582-1586.

(9) Wu H, Ding AA, De G, V. Estimation of HIV dynamic parameters. Stat Med 1998; 17(21):2463-2485.

(10) Wu H, Ding AA. Population HIV-1 dynamics in vivo: applicable models and inferential tools for virological data from AIDS clinical trials. Biometrics 1999; 55(2):410-418.

APPENDIX II: A5248 SPECIMEN COLLECTION, PROCESSING, AND SHIPMENT

1.0 OVERVIEW

All specimens will be collected per Section 6.0, Clinical and Laboratory Evaluations. Collection, processing, and shipment details for select specimens are below. For instructions regarding specimens not listed on this table, please refer to the Laboratory Processing Chart (LPC). All specimens generated from this protocol must be labeled, stored, and shipped according to the LDMS guidelines unless otherwise specified.

1. TABLE

| Assay/Procedure | Tube Type or Specimen Type and Quantity | Derivative | Processing Instructions | Aliquots | Shipping Instructions |
| --- | --- | --- | --- | --- | --- |
| VIROLOGY |  |  |  |  |  |
| Plasma HIV-1 RNA (batched and real time) | 1 x 6-mL whole blood EDTA (purple top) tube, according to the Schedule of Events in section 6.0 | Plasma | Follow ACTG guidelines for processing. | 2 x 1-mL plasma  LDMS code:  BLD/EDT/PL2 | **Batch and ship samples from entry through day 21 to Johns Hopkins University when instructed by the team.**  **Ship 2 x 1-mL aliquots at all other timepoints** real time to Johns Hopkins University. |
| HIV-1 RNA Single Copy Assay (batched) | **2** x 10-mL whole blood EDTA (purple top ) tube at Entry, **Days 2 and 7**  **3** x 10-mL whole blood EDTA (purple top) tube at Days **10** and 14  3 x 10-mL and 1 x 5-mL (35 mL total) whole blood EDTA (purple top) tube at Days 21, 28, and 56  5 x 10-mL whole blood EDTA (purple top) tube at Weeks 12, 16, 20, 24, 36, 48, 60, and 72/Final Visit | Plasma | **Process within 4 hours. Spin blood at 400 x g for 10 minutes in a table top centrifuge. Remove plasma very carefully without disturbing the buffy coat and/or mixing cells into the plasma.** **To prevent contamination of the plasma with cells, leave about 0.5 mL of plasma behind on top of cells. This is very important. Re spin plasma at 1350 x g for 15 min. Again leave about 0.5mL of plasma behind to prevent cell contamination. Save this last 0.5mL in a separate cryovial for 16s DNA testing.** If cells are inadvertently mixed with the plasma, re-spin the sample and remove the plasma without disturbing the cells  NOTE: When a sample is processed for the single copy assay, a sufficient layer of plasma must be left behind to ensure that absolutely no cells in the buffy coat or red blood cell layers are removed with the plasma. | As many 1.5-mL plasma aliquots as possible  LDMS code:  BLD/EDT/PL1 | Ship according to current BRI guidelines. |
| Proviral DNA, LTR Circular DNA, Integrated Proviral DNA | No additional blood. PBMCs obtained through HIV-1 RNA single copy assay blood, according to the Schedule of Events in section 6.0 | PBMC | Follow ACTG guidelines for processing and cryopreservation. | As many 5 x 10^6^ cell aliquots of viable PBMCs as possible  LDMS code:  BLD/EDT/CEL/DMS | Ship according to current BRI guidelines. |
| Genotype | 1x10-mL whole blood EDTA (purple top) tube, according to the Schedule of Events in section 6.0 | Plasma | Follow ACTG guidelines for processing. | 4x1 mL plasma  LDMS code:  BLD/EDT/PL2 | Ship 4x1 mL plasma real time to Harvard University. |
| Stored plasma | 2 x 10-mL whole blood EDTA (purple top) tube, **according to the Schedule of Events in section 6.0** | Plasma | Follow ACTG guidelines for processing. | 6 x 1 mL plasma at all other timepoints  LDMS code:  BLD/EDT/PL2 | Ship according  to current BRI guidelines. |
| Stored PBMC | No additional blood. PBMCs obtained through stored plasma at weeks 24, 36, 48, 60, 72/final visit, **and virologic failure confirmation** and through HIV-1 RNA single copy assay blood **at entry and on** days 2, 7, 10, 14, 21, 28, 56 | PBMC | Follow ACTG guidelines for processing and cryopreservation. | 6 (5 x 10^6^) PBMC  LDMS code : BLD/EDT/CEL/DMS | Ship according  to current BRI guidelines. |
| IMMUNOLOGY |  |  |  |  |  |
| CD4+/CD8+ | 1 x 4-mL whole blood EDTA (purple top) tube, **c** | N/A | Send ambient to local processing lab. | N/A | N/A |
| Advanced Flow Assay | **2 x 10-mL whole blood EDTA (purple top) tube at Pre-Entry**  No additional blood **at other time points**. PBMCs obtained through HIV-1 RNA single copy assay blood, according to the Schedule of Events in section 6.0 | PBMC | Follow ACTG guidelines for processing. | As many 5 x 10^6^ cell aliquots of viable PBMCs as possible  LDMS code:  BLD/EDT/CEL/DMS | Ship according to current BRI guidelines. |
| Plasma Bacterial 16s DNA | **No additional blood. Plasma will be obtained from advanced flow assay blood at Pre-Entry and from stored plasma at Virologic Failure Confirmation**.  **At all other time points plasma will be obtained from 0.5 mL remainder of HIV-1 RNA single copy assay plasma** | **Plasma** | **Please follow HIV-1 RNA Single Copy Assay processing instructions. The 0.5 mL of plasma must be taken from the remaindered plasma after the second spin.** | **1 X 0.5 mL LDMS code:**  **BLD/EDT/PL2** | **Ship according to current BRI guidelines.** |
| PHARMACOLOGY |  |  |  |  |  |
| Pharmacokinetic Studies | 1 x **3**-mL whole blood EDTA (purple top) tube **according to the Schedule of Events in section 6.0** | Plasma | Follow ACTG guidelines for processing. See clinical considerations (section 7.0) below. | **2 x 1 mL**  LDMS code:  BLD/EDT/PL1 | **Ship according to current BRI guidelines.** |

#### 3.0 VIROLOGY CONSENSUS METHODOLOGY LINKS

- ACTG Laboratory Manual:

[http://aactg.s-3.com/LabManual.htm](http://aactg.s-3.com/LabManual.htm )

- ACTG Processing Guide Index:

<http://aactg.s-3.com/pub/download/labmanual/12-ALM-Specimen-Processing.pdf>

- PBMC Cryopreservation Consensus Method:

[http://aactg.s-3.com/pub/download/labmanual/13-ALM-PACTG-PBMC- Processing-Cryopreservation-Tha.pdf](http://aactg.s-3.com/pub/download/labmanual/13-ALM-PACTG-PBMC-%09Processing-Cryopreservation-Tha.pdf)

4.0 IMMUNOLOGY CONSENSUS METHODOLOGY LINKS

- 3-Color Flow Cytometry Consensus Method:

<http://aactg.s-3.com/pub/download/labmanual/24-ALM-Consensus-Method-for-3-color-Advanced-Flow.pdf>

5.0 PHARMACOLOGY CONSENSUS ASSAY METHOD LINKS

- Pharmacology Tutorial “ACTG Pharmacology Study Conduct: Impact on PK-PD Analysis”:

<http://aactg.s-3.com/members/inaphqac.htm>

- Pharmacology Sample Processing Guidelines:

<http://aactg.s-3.com/members/inaphqac.htm>

6.0 SHIPPING CONSENSUS METHODOLOGY LINKS

- Guidelines for Shipping Diagnostic Specimens:

<http://aactg.s-3.com/pub/download/labmanual/22-ALM-Diagnostic-Shipping-Guidelines.pdf>

- ACTG Specimen Repository (BRI) Shipping Guidelines:

<http://aactg.s-3.com/specrepos.htm>

- Outsourcing Laboratories (JHU) Shipping Instructions:

<http://aactg.s-3.com/laboutsourcing.htm>

- PSL Shipping Guidelines:

<http://aactg.s-3.com/members/inaphqac.htm>

1. CLINICAL CONSIDERATIONS
   1. Pharmacokinetic Studies

Blood samples should be kept on ice until processed. Each blood sample should be processed, and the plasma separated by centrifugation, within 90 minutes of the time of collection. Centrifuge the blood sample at 800g for 10 minutes to obtain the plasma. Transfer the plasma to a labeled polypropylene cryovial that has a screw top. The plasma should be kept in an ice bath until it is frozen. Freeze the cryovials in the upright position and maintain frozen at -20˚C to -70˚C until shipment.

# APPENDIX III: A5249s SPECIMEN COLLECTION, PROCESSING, AND SHIPMENT

1.0 OVERVIEW

All specimens will be collected per Section 6.0, Clinical and Laboratory Evaluations. Collection, processing, and shipment details for select specimens are below. For instructions regarding specimens not listed on this table, please refer to the LPC. All specimens generated from this protocol must be labeled, stored, and shipped according to the LDMS guidelines unless otherwise specified.

1. TABLE

| Assay/Procedure | Tube Type or Specimen Type and Quantity | Derivative | Processing Instructions | Aliquots | Shipping Instructions |
| --- | --- | --- | --- | --- | --- |
| VIROLOGY |  |  |  |  |  |
| Plasma HIV-1 RNA | 1 x 6-ml whole blood EDTA (purple top) tube, according to the schedule of events in section 6.0 | Plasma | Follow ACTG guidelines for processing | Prepare at least 2 x 1-ml  Preparation of additional aliquots is strongly encouraged.  Store on site at -70°C  LDMS code: BLD/EDT/PL2 | Ship two (2) 1-ml aliquots from visit days 1 through 7 to the Johns Hopkins University for viral load analysis. Ship remaining aliquots according to current BRI guidelines. |

#### VIROLOGY CONSENSUS METHODOLOGY LINKS

- ACTG Laboratory Manual:

[http://aactg.s-3.com/LabManual.htm](http://aactg.s-3.com/LabManual.htm )

- ACTG Processing Guide Index:

<http://aactg.s-3.com/pub/download/labmanual/12-ALM-Specimen-Processing.pdf>

1. SHIPPING CONSENSUS METHODOLOGY LINKS

- Guidelines for Shipping Diagnostic Specimens:

<http://aactg.s-3.com/pub/download/labmanual/22-ALM-Diagnostic-Shipping-Guidelines.pdf>

- Guidelines for Shipping Infectious Substances:

<http://aactg.s-3.com/pub/download/labmanual/20-ALM-Infectious-Shipping-Guidelines.pdf>

- ACTG Specimen Repository (BRI) Shipping Guidelines:

<http://aactg.s-3.com/specrepos.htm>

- Outsourcing Laboratories (JHU/Quest) Shipping Instructions:

<http://aactg.s-3.com/laboutsourcing.htm>

- PSL Shipping Guidelines:

<http://aactg.s-3.com/members/inaphqac.htm>

APPENDIX IV

DIVISION OF AIDS

AIDS CLINICAL TRIALS GROUP (ACTG)

### SAMPLE INFORMED CONSENT

For protocol:

A5248, DRAFT Version 0.13, 11/30/07: First-Phase Viral Decay Rates in Treatment-Naïve Subjects Initiating Treatment with Raltegravir (MK-0518) and Emtricitabine (FTC)/Tenofovir

Disoproxil Fumarate (TDF): A Pilot Study

SHORT TITLE FOR THE STUDY: First-Phase Viral Decay Rates with Raltegravir and FTC/TDF

# INTRODUCTION

You are being asked to take part in this research study because you are infected with HIV, the virus that causes AIDS, and have never received treatment for your HIV. This study is sponsored by the National Institutes of Health (NIH). The doctor in charge of this study at this site is: (insert name of Principal Investigator). Before you decide if you want to be a part of this study, we want you to know about the study.

This is a consent form. It gives you information about this study. The study staff will talk with you about this information. You are free to ask questions about this study at any time. If you agree to take part in this study, you will be asked to sign this consent form. You will get a copy to keep.

# WHY IS THIS STUDY BEING DONE?

The purpose of this study is to learn:

- How well this combination of drugs lowers the amount of HIV in your blood (your viral load),
- How well you tolerate this drug combination,
- How safe this combination of drugs is.

The drugs used in this study are raltegravir and tenofovir disoproxil fumarate (TDF)/emtricitabine (FTC). Raltegravir is an investigational drug and is not approved by the Food and Drug Administration (FDA). In studies so far, raltegravir has been shown to block HIV growth and lower the amount of virus in the blood in the majority of infected people studied. FTC/TDF is approved by the FDA for treating HIV.

WHAT DO I HAVE TO DO IF I AM IN THIS STUDY?

If you agree to join this study, you will be asked to sign this consent form. After you have signed the form, you will be asked some questions and will undergo some tests at the screening visit to see if it is safe for you to join the study. The screening visit will take about 30-60 minutes.

At Screening

- You will be asked about your health and any medicines you have taken in the last 30 days.
- If you are a woman able to become pregnant, you will have a pregnancy test. Pregnant women cannot enter the study. If you become pregnant, you will have to stop participating in the study.
- You will have about **1-2** tablespoons of blood drawn for routine blood tests, HIV viral load, and resistance testing.
- You will be asked to give a urine specimen.

If You Do Not Enroll Into the Study

If you decide not to take part in this study or if you do not meet the eligibility requirements, we will still use some of your information. As part of this screening visit, some demographic (e.g., age, gender, race), clinical (e.g., disease condition, diagnosis), and laboratory (e.g., CD4 cell count, viral load) information is being collected from you so that ACTG researchers may help determine whether there are patterns or common reasons why people do not join a study.

Pre-Entry

If you have met all the requirements to enter the study, you will come to the clinic at least 48 hours after the screening visit for pre-entry evaluations. This visit will last about 30-60 minutes.

- You will have a physical exam. The clinic staff will check your weight, height, and vital signs such as temperature, blood pressure, breathing, and pulse.
- You will have about 2 tablespoons of blood drawn for routine blood tests, HIV viral load, and CD4+ and CD8+ cell count (the number of white blood cells that fight infection.
- **Extra blood will be stored for future immunology tests.**

Entry

At least 48 hours after, but within 14 days of your pre-entry visit, you will come to the clinic for entry evaluations. This visit will last about 30-60 minutes.

- You will be asked about any medicine changes you have had since screening.
- You will have a physical exam. The clinic staff will check your weight and vital signs such as temperature, blood pressure, and pulse.
- If you are a woman able to become pregnant, you will have a pregnancy test. Pregnant women cannot enter the study. If you become pregnant, you will have to stop participating in the study.
- You will have about 3 tablespoons of blood drawn for routine blood tests, HIV viral load, and CD4+ and CD8+ cell count.
- Extra blood will be stored for future immunology and virology tests.

**The study drugs** raltegravir **and FTC/TDF will be given to you at this visit. Raltegravir is provided by the study, however, FTC/TDF is not provided by the study.** You will take raltegravir twice a day by mouth. You will take FTC/TDF once a day by mouth.

Post-Entry

**After you start taking the study drugs, you will be asked to come to the clinic on Days 2, 7, 10, 14, 21, 28, and 56. You will also return to the clinic at Weeks 12, 16, 20, 24, 36, 48, 60, and 72. At every visit your blood will be collected for routine blood tests,** HIV viral load, CD4+ and CD8+ cell count, **and to measure the amount of raltegravir and FTC/TDF in your blood**. **At some visits you will have a physical exam and extra blood will be stored for future immunology and virology tests.** You will be asked if you missed any doses of study drugs. Most visits will last about 30-60 minutes.

**At Weeks 24, 48, and 72 you will be asked to give a urine specimen. At all visits from Day 28 until the end of the study, you will be asked about changes to any other medicines that you may be taking.**

If you are a woman able to become pregnant, a urine or serum pregnancy test will be done at any visit if pregnancy is suspected to make sure that you are not pregnant.

**You will be given the results of the pregnancy (if applicable), CD4+ and CD8+ cell counts, viral load, and routine blood tests from all visits as soon as they become available.**

If at any point during the study you are found to have a viral load value that is detectable you will be asked to come back in for some additional tests along with another viral load test to make sure the first one was correct. If your viral load has not decreased enough or has increased in the second viral load test, your doctor will take you off study. This visit will last about 30-60 minutes.

- You will be asked about any medicine changes you have had since your last visit.
- You will have a physical exam. The clinic staff will check your weight and vital signs such as temperature, blood pressure, and pulse.
- You will have about 2 tablespoons of blood drawn for routine blood tests, HIV viral load, CD4+ and CD8+ cell count, and resistance testing.
- Extra blood will be stored for future immunology and virology tests.

If you stop taking the study drugs before the end of the study, you will be asked to continue to be part of the study and return to the clinic for additional evaluations.

- You will be asked about any medicine changes you have had since your last visit.
- You will have a physical exam. The clinic staff will check your weight and vital signs such as temperature, blood pressure, and pulse.
- You will have about 2 tablespoons of blood drawn for routine blood tests, HIV viral load, and CD4+ and CD8+ cell count.
- You will be asked to give a urine specimen.

If you are taken off study early or decide to leave the study early, you will be asked to return to the clinic for a final visit. This visit will last about 30-60 minutes.

- You will be asked about any medicine changes you have had since your last visit.
- You will have a physical exam. The clinic staff will check your weight and vital signs such as temperature, blood pressure, and pulse.
- You will have about **6** tablespoons of blood drawn for routine blood tests, HIV viral load, and CD4+ and CD8+ cell count.
- You will be asked to give a urine specimen.
- Extra blood will be stored for future immunology and virology tests.

Other

Some of your blood will be stored (with usual protectors of identity) and used for immunology, virology, and metabolic testing that is required for this study.

Some of your blood that is leftover after all required study testing is done may be stored (with usual protectors of identity) and used for ACTG-approved HIV-related research. Storage of leftover blood is not a requirement to participate in the study and you may withdraw your approval for the storage of your leftover blood, at anytime. These samples may be held for an indefinite length of time. We cannot ensure that you will be told of the results of the research done on these samples. Please indicate below whether you approve the use of your leftover blood.

________ YES ________ NO

HOW MANY PEOPLE WILL TAKE PART IN THIS STUDY?

About 34 people will take part in this study

HOW LONG WILL I BE IN THIS STUDY?

You will be in this study for about 72 weeks.

WHY WOULD THE DOCTOR TAKE ME OFF THIS STUDY EARLY?

The study doctor may need to take you off the study early without your permission if:

- the study is cancelled by the ACTG, U.S. Food and Drug Administration (FDA), National Institutes of Health (NIH), the Office for Human Research Protections, the drug company supporting this study, or the site’s Institutional Review Board (IRB). (An IRB is a committee that watches over the safety and rights of research subjects.)
- a safety monitoring committee recommends that the study be stopped early.
- you are not able to attend the study visits as required by the study.
- you are not taking the study drugs as required by the study.
- your viral load is confirmed to be detectable on more than one measurement.

The study doctor may also need to take you off the study drug(s) without your permission if:

- continuing the study drugs may be harmful to you.
- you need a treatment that you may not take while on the study.
- you become pregnant or are breast-feeding.

If I have to permanently stop taking study-provided drug, or once I leave the study, how would the drug be provided?

During the study:

If you must permanently stop taking study-provided drug before your study participation is over, the study staff will discuss other options that may be of benefit to you.

After the study:

After you have completed your study participation, the study will not be able to continue to provide you with drug you received on the study. If continuing to take these or similar drugs/agents would be of benefit to you, the study staff will discuss how you may be able to obtain them.

WHAT ARE THE RISKS OF THE STUDY?

The drugs used in this study may have side effects, some of which are listed below. Please note that these lists do not include all the side effects seen with these drugs. These lists include the more serious or common side effects with a known or possible relationship. If you have questions concerning the additional study drug side effects please ask the medical staff at your site.

There is a risk of serious and/or life-threatening side effects when non-study medications are taken with the study drugs. For your safety, you must tell the study doctor or nurse about all medications you are taking (including nutritional supplements and herbal medications) before you start the study and also before starting any new medications while on the study. Also, you must tell the study doctor or nurse before enrolling in any other clinical trials while on this study.

Risks of Drawing Blood

Taking blood may cause discomfort, bleeding, and bruising where the blood is drawn. Occasionally, there is swelling in the area where the needle enters the body and there is a small risk of infection. There is also a risk of lightheadedness, fainting, and blood clots.

Risks of Combination Antiretroviral Therapy

Immune Reconstitution Inflammatory Syndrome (IRIS): In some people with advanced HIV infection, signs and symptoms of inflammation from other infections may occur soon after anti-HIV treatment is started.

The use of potent ARV drug combinations may be associated with an abnormal placement of body fat and wasting. Some of the body changes include:

- Increase in fat around the waist and stomach area
- Increase in fat on the back of the neck
- Thinning of the face, legs, and arms
- Breast enlargement

Risks with Use of Raltegravir

The side effects listed below may be incomplete and our understanding of the safety of the drug may change during the course of this study. Several hundred people have received raltegravir in research studies.

The following side effects have been associated with the use of raltegravir in combination with other HIV drugs:

- Upset stomach (nausea, diarrhea, vomiting)
- Unusual tiredness
- Headache
- Dizziness
- Trouble sleeping, abnormal dreams
- Gas
- Itching
- Sleepiness
- Altered taste
- Loss/lack of appetite
- Constipation
- Abdominal pain
- Abnormal placement of body fat
- Muscle spasms
- Rash
- Stomach sticking out (abdominal distension)

Abnormal blood tests which have been seen in studies of raltegravir in combination with other HIV drugs include:

- Elevated liver enzymes, and bilirubin
- Increased levels of muscle enzymes
- Increased pancreas enzymes
- Low white blood cell counts or red blood cell counts
- Increases in cholesterol or triglycerides
- Worsening in a kidney function test

Some rare serious side effects have been seen in studies of raltegravir in combination with other HIV drugs, and have each been reported in one or more subjects.

- Pancreatitis (swelling of the pancreas)
- Excessive acid in the blood and body
- Kidney failure
- Stomach irritation
- Allergic reaction
- Heart attack
- Hepatitis (liver problems)

Cancers have been seen both in people who took raltegravir with other HIV drugs and in people receiving placebo plus other HIV drugs. Some were cancers that the people had before. Several cancers were seen in the first 3 months of treatment. The specific cancers were cancers seen previously in people with very sick immune systems. It is unknown if the cancers were related to raltegravir use.

Risks with Use of Nucleoside Analogues

Lactic acidosis and severe hepatomegaly (enlarged liver) with steatosis (fatty liver) that may result in liver failure, other complications and death have been reported with the use of ARV nucleoside analogues alone or in combination. The liver complications and death have been seen more often in women on these drug regimens. Some nonspecific symptoms that might indicate lactic acidosis include: unexplained weight loss, stomach discomfort, nausea, vomiting, fatigue, weakness, and shortness of breath.

Risks with Use of Tenofovir Disoproxil Fumarate (TDF)

The following side effects have been associated with the use of tenofovir:

- Upset stomach, vomiting, gas, loose or watery stools
- Dizziness
- Abdominal pain
- Lack of energy
- Kidney damage or failure
- Inflammation or swelling and possible damage to the pancreas
- Shortness of breath
- Rash
- Low phosphate, a chemical in the blood
- Increase of liver functions tests in children
- Allergic reaction, which may include fever, rash, upset stomach, vomiting, loose or watery stools, abdominal pain, achiness, shortness of breath or a general feeling of illness
- Changes in bone growth and strength were seen in study animals given tenofovir. Bone thinning has been seen in adults and children taking tenofovir.

NOTE: If you are infected with both Hepatitis B and HIV, you should be aware that your liver function tests may increase, and symptoms associated with hepatitis (an acute inflammation of the liver) may worsen if tenofovir is stopped.

NOTE: Because there is only a small amount of information on tenofovir in pregnant women, tenofovir should be used during pregnancy only if clearly needed.

Risks with Use of Emtricitabine (FTC)

The following side effects have been associated with the use of emtricitabine:

- Headache
- Dizziness
- Tiredness
- Inability to sleep, unusual dreams

# Loose or watery stools

- Upset stomach (nausea) or vomiting
- Abdominal pain
- Rash, itching, which sometimes can be a sign of an allergic reaction
- Skin darkening of the palms and/or soles
- Increased cough
- Runny nose

# Abnormal liver function tests, which could mean liver damage

- Increases in pancreatic enzyme (substances in the blood), which could mean a problem with the pancreas
- Increased triglycerides
- Increased creatine phosphokinase (CPK), which could mean muscle damage

NOTE: If you are infected with both Hepatitis B and HIV, you should be aware that your liver function tests may increase, and symptoms associated with hepatitis (an acute inflammation of the liver) may worsen if emtricitabine is stopped.

Risks with Use of Emtricitabine/Tenofovir Disoproxil Fumarate (FTC/TDF)

No new or unexpected side effects are observed with the TDF 300/ FTC 200 mg combination tablet than those observed when each drug is given separately.

ARE THERE RISKS RELATED TO PREGNANCY?

The drugs used in this study may be unsafe for unborn babies. If you are having sex that could lead to pregnancy, you must agree not to become pregnant or make someone else pregnant. Because of the risk involved, you and your partner must use at least two methods of birth control. You must continue to use birth control until 60 days after stopping your medicines. You must choose two of the birth control methods listed below:

- Condoms (male or female) with or without a spermicidal agent
- Diaphragm or cervical cap with spermicide
- IUD
- Hormone-based contraception

If you can become pregnant, you must have a pregnancy test before you enter this study. The test must show that you are not pregnant. If you think you may be pregnant at any time during the study, tell your study staff right away. If you become pregnant while on study, you must stop study treatment, but will be asked to continue to come in for study visits.

Breastfeeding

It is unknown whether the study drug passes through the breast-milk and may cause harm to your infant. You must not breast-feed if you are in this study.

WHAT IF I BECOME PREGNANT DURING THIS STUDY?

If you become pregnant during the study, you must stop the study treatment but you will be asked to continue to come in for study visits. At these visits, you will have safety evaluations including routine safety tests, CD4 counts, viral loads and physical exams on the same schedule as described earlier in this consent. You will have about 2-3 teaspoons of blood drawn at each study visit for these tests.

You and your physician will decide what anti-HIV drug combination would be best for you to continue. This study will not provide care related to your pregnancy, the delivery of your baby, or the care of your baby. You must arrange for your care and your baby’s care outside of this study. This study will not provide your baby any anti-HIV drugs. Long-term follow-up is recommended for a baby whose mother takes anti-HIV drugs during pregnancy. The study staff will talk to you about your choices for long-term follow up.

ARE THERE BENEFITS TO TAKING PART IN THIS STUDY?

If you take part in this study, there may be a direct benefit to you, but no guarantee can be made. It is also possible that you may receive no benefit from being in this study. Information learned from this study may help others who have HIV.

WHAT OTHER CHOICES DO I HAVE BESIDES THIS STUDY?

Instead of being in this study you have the choice of:

- treatment with prescription drugs available to you
- treatment with experimental drugs, if you qualify
- no treatment

Please talk to your doctor about these and other choices available to you. Your doctor will explain the risks and benefits of these choices.

WHAT ABOUT CONFIDENTIALITY?

We will do everything we can to protect your privacy. In addition to the efforts of the study staff to help keep your personal information private, we have gotten a Certificate of Confidentiality from the U.S. Federal Government. This certificate means that researchers cannot be forced to tell people who are not connected with this study, such as the court system, about your participation. Also, any publication of this study will not use your name or identify you personally.

People who may review your records include the ACTG, U.S. Food and Drug Administration (FDA), (insert name of site) IRB, National Institutes of Health (NIH), study staff, study monitors, drug companies supporting this study, and their designees. Having a Certificate of Confidentiality does not prevent you from releasing information about yourself and your participation in the study.

Even with the Certificate of Confidentiality, if the study staff learns of possible child abuse and/or neglect or a risk of harm to yourself or others, we will be required to tell the proper authorities.

WHAT ARE THE COSTS TO ME?

Taking part in this study may lead to added costs to you and your insurance company. In some cases it is possible that your insurance company will not pay for these costs because you are taking part in a research study.

WHAT HAPPENS IF I AM INJURED?

If you are injured as a result of being in this study, you will be given immediate treatment for your injuries. The cost for this treatment will be charged to you or your insurance company. There is no program for compensation either through this institution or the National Institutes of Health. You will not be giving up any of your legal rights by signing this consent form.

WHAT ARE MY RIGHTS AS A RESEARCH SUBJECT?

Taking part in this study is completely voluntary. You may choose not to take part in this study or leave this study at any time. You will be treated the same no matter what you decide.

We will tell you about new information from this or other studies that may affect your health, welfare, or willingness to stay in this study. If you want the results of the study, let the study staff know.

WHAT DO I DO IF I HAVE QUESTIONS OR PROBLEMS?

For questions about this study or a research-related injury, contact:

- name of the investigator or other study staff
- telephone number of above

For questions about your rights as a research subject, contact:

- name or title of person on the Institutional Review Board (IRB) or other organization appropriate for the site
- telephone number of above

SIGNATURE PAGE

If you have read this consent form (or had it explained to you), all your questions have been answered and you agree to take part in this study, please sign your name below.

____________________________ _________________________________________

Participant’s Name (print) Participant’s Signature and Date

____________________________ _________________________________________

Participant’s Legal Guardian (print) Legal Guardian’s Signature and Date

(As appropriate)

____________________________ _________________________________________

Study Staff Conducting Study Staff’s Signature and Date

Consent Discussion (print)

____________________________ _________________________________________

Witness’s Name (print) Witness’s Signature and Date

(As appropriate)

APPENDIX V

DIVISION OF AIDS

AIDS CLINICAL TRIALS GROUP (ACTG)

### SAMPLE INFORMED CONSENT

For protocol:

A5249s, DRAFT Version 0.13, 11/30/07: A5249s: Intensive Viral Dynamics Substudy of A5248

SHORT TITLE FOR THE STUDY: Viral Dynamics Subsudy

# INTRODUCTION

You are being asked to take part in this research substudy because you will be taking raltegravir and tenofovir DF (TDF)/emtricitabine (FTC) for your HIV infection on the main study A5248. This study is sponsored by the National Institutes of Health (NIH). The doctor in charge of this substudy at this site is: (insert name of Principal Investigator). Before you decide if you want to be a part of this substudy, we want you to know about the substudy.

This is a consent form. It gives you information about this substudy. The substudy staff will talk with you about this information. You are free to ask questions about this substudy at any time. If you agree to take part in this substudy, you will be asked to sign this consent form. You will get a copy to keep.

# WHY IS THIS SUBSTUDY BEING DONE?

The purpose of this substudy is to learn when the amount HIV virus in your blood (viral load) begins to decrease once you start raltegravir and FTC/TDF.

WHAT DO I HAVE TO DO IF I AM IN THIS SUBSTUDY?

If you agree to join this substudy, you will be asked to sign this consent form.

Day 0 to Day 2

On Day 0, the day you begin taking your study drugs on the main study (A5248), you will be asked to come into the clinic in the morning and stay for the next 2 days and nights (Day 0 to Day 2). You will have your blood drawn 11 times to measure your viral load while you are in the hospital. You will have about 15 tablespoons of blood drawn.

On Day 2 when you leave the hospital, you will be given a diary card to write down when you take your study drugs on Days 2 to 7.

Days 3, 4, and 7

You will return to the clinic on Days 3, 4, and 7 to have your blood drawn to measure your viral load. You will have 1 tablespoon of blood drawn each day.

You will be asked to bring your diary card to the clinic for your visits on Days 3, 4, and 7.

Premature Discontinuation of Substudy A5249s

If you decide to discontinue your participation in this substudy, you may continue in the main study A5248. If you discontinue your participation in the main study, then you will be removed from this substudy as well.

Other

Some of your blood will be stored (with usual protectors of identity) and used for virologic testing that is required for this study.

Some of your blood that is leftover after all required study testing is done may be stored (with usual protectors of identity) and used for ACTG-approved HIV-related research. Storage of leftover blood is not a requirement to participate in the study and you may withdraw your approval for the storage of your leftover blood, at anytime. These samples may be held for an indefinite length of time. We cannot ensure that you will be told of the results of the research done on these samples. Please indicate below whether you approve the use of your leftover blood.

________ YES ________ NO

HOW MANY PEOPLE WILL TAKE PART IN THIS SUBSTUDY?

About 10 people will take part in this study.

HOW LONG WILL I BE IN THIS SUBSTUDY?

You will be in this study for about 10 days.

WHAT ARE THE RISKS OF THE SUBSTUDY?

Risks of Drawing Blood

Taking blood may cause discomfort, bleeding, and bruising where the blood is drawn. Occasionally, there is swelling in the area where the needle enters the body and there is a small risk of infection. There is also a risk of lightheadedness, fainting, and blood clots.

ARE THERE BENEFITS TO TAKING PART IN THIS SUBSTUDY?

There is no direct benefit to you from being in this substudy. Knowledge from this substudy may help others with HIV infection in the future.

WHAT ARE THE COSTS TO ME?

Taking part in this substudy may lead to added costs to you and your insurance company. In some cases it is possible that your insurance company will not pay for these costs because you are taking part in a research study.

WILL I RECEIVE ANY PAYMENT?

You will be paid ________ for participation in the substudy. *(The team recommends compensation to subjects of $200/night in the hospital. Sites will be reimbursed for the expense.)*

All other information that is contained in the main study (A5248) consent you signed, applies to this substudy consent as well.

SIGNATURE PAGE

If you have read this consent form (or had it explained to you), all your questions have been answered and you agree to take part in this study, please sign your name below.

____________________________ _________________________________________

Participant’s Name (print) Participant’s Signature and Date

____________________________ _________________________________________

Participant’s Legal Guardian (print) Legal Guardian’s Signature and Date

(As appropriate)

____________________________ _________________________________________

Study Staff Conducting Study Staff’s Signature and Date

Consent Discussion (print)

____________________________ _________________________________________

Witness’s Name (print) Witness’s Signature and Date

(As appropriate)

1. For these back-calculations, see ~sue/VDynamics/programs/ From.A5166sMs.sas. [↑](#footnote-ref-2)
